# Scandinavian pattern and temperature changes shape European summer droughts over the past millennium

**DOI:** 10.1038/s41467-026-72385-w

**Published:** 2026-04-27

**Authors:** Huihong Xue, Hugues Goosse, Quentin Dalaiden, Kristina Seftigen, Fabio Gennaretti, Feng Shi

**Affiliations:** 1 Earth and Life Institute, Université catholique de Louvain, Louvain-la-Neuve, Belgium; 2 State Key Laboratory of Lithospheric and Environmental Coevolution, Institute of Geology and Geophysics, Chinese Academy of Sciences, Beijing, China; 3 Bjerknes Center for Climate Research, Bergen, Norway; 4 Nansen Environmental and Remote Sensing Center, Bergen, Norway; 5 Gothenburg University Laboratory for Dendrochronology, Department of Earth Sciences, University of Gothenburg, Gothenburg, Sweden; 6 Department of Agricultural, Food and Environmental Sciences, Università Politecnica delle Marche, Ancona, Italy; 7 Institut de Recherche sur les Forêts, Groupe de Recherche en Écologie de la MRC-Abitibi, Université du Québec en Abitibi-Témiscamingue, Amos, Canada

**Keywords:** Environmental impact, Palaeoclimate

## Abstract

Recent decades have seen pronounced changes in European hydroclimate, including widespread summer drying, yet its spatiotemporal variability and underlying drivers remain uncertain. Here we present the European Last Millennial Data Assimilation (EULMDA), a new reconstruction of European hydroclimate and its main drivers covering the past millennium. EULMDA integrates five Earth System Model simulations with over one hundred moisture and temperature sensitive tree-ring records. It demonstrates high skill in reproducing instrumental variability across climate variables, including large-scale atmospheric circulation changes. We show that European warm-season drought variability is primarily governed by circulation changes associated with the Scandinavian pattern (SCAND) and long-term summer temperature changes, together explaining over half of the spatiotemporal drought variance. SCAND drives a pronounced north–south dipole in summer hydroclimate, explaining a larger fraction of Mediterranean drought variability than other major circulation modes, contributing to recent multidecadal drying in the Mediterranean alongside wetting in northern Europe. Meanwhile, summer warming intensifies drying across much of Europe. These results provide critical context for interpreting recent drought trends and insight into mechanisms shaping future hydroclimate risks.

## Abstract (web summary)

Two main drivers shaped European summer drought over the past millennium: atmospheric circulation created contrasts between Scandinavia and Southern Europe, while temperature changes drove homogenous drying and wetting at European scale.

## Introduction

Drought is one of the most significant climate-related hazards in Europe, with profound socio-economic and environmental consequences^1–5^. In recent decades, northern Europe has become wetter, while southern Europe has experienced increasing dryness, forming a distinct north–south dipole pattern^6–11^. Drought can be understood as a prolonged imbalance between water supply and demand, occurring when precipitation (water supply) is insufficient to meet evaporative needs (water demand)^12^. Both thermodynamic and dynamic mechanisms contribute to this imbalance^13,14^. The local hydroclimate consequences of warming and the associated thermodynamic adjustments of the hydrological cycle are relatively well understood. The first effect is a greater atmospheric evaporative demand that induces drying. Positive feedback between a decline in soil moisture, low precipitation, and surface temperature can then be activated^2,15–18^. The warming over the past century in southern Europe and the Mediterranean region could have thus contributed to increasingly arid conditions during the warm season there^19,20^. However, such an increase in evaporative demand cannot explain the wetting tendency observed during the last 150 years in northern Europe^21,22^.

On the other hand, moisture transport and precipitation are controlled by dynamical factors, i.e., the atmospheric circulation^14,23^. During the cold season, the North Atlantic Oscillation (NAO) is considered the main driver of surface climate variability in Europe, with a dominant influence on precipitation and drought patterns over most of the continent^24–27^. The link between circulation and drought variability and trends appears to be more complex and elusive in summer. The summer NAO is weaker and confined to northern latitudes compared to its winter counterpart, with a high-pressure center typically located over the British Isles and the North Sea region^28^. Although it has a broad impact on precipitation across some regions of the European continent, only a weak effect on drought in southern Europe/the Mediterranean is observed^29^. Additionally, the jet stream over the North Atlantic-European sector is considered an important dynamical driver due to its influence on storm tracks or its role in the development of atmospheric blocking^30^. However, various studies have reached divergent conclusions regarding its influence on past climate variability, possibly due to differences in how the jet stream is defined or characterized^31–33^. Another circulation pattern, the Scandinavian pattern (SCAND), is also associated with high-latitude blocking over Europe, characterized by a persistent high-pressure center around the Fennoscandia region^34^. Some studies have suggested that SCAND may explain a fraction of the temperature and precipitation variability not accounted for by the NAO in parts of Europe^35,36^.

Thermodynamic and dynamic processes frequently operate in concert. The resulting complex physical mechanisms explaining summer drought variability and trends across the whole of Europe remain uncertain, deserving further study. A more complete understanding of natural drought variability and anthropogenically forced moisture trends requires investing the long-term variability, which is not available from relatively short instrumental records. Therefore, in this study, we present the European Last Millennial Data Assimilation (EULMDA), a physically coherent, annually resolved hydroclimate reconstruction generated using offline Data Assimilation (DA) based on a particle filter algorithm and covering the past millennium (1000–2000 CE; “Methods”). EULMDA assimilates more than one hundred tree-ring-based records across Europe, including indicators such as ring width, oxygen isotopes, and wood density, which are sensitive to temperature, moisture conditions, or both (Supplementary Fig. 1). The largest subset of records (*n* = 42) is sensitive to June–August (JJA) temperature or precipitation, followed by records sensitive to March–September climate. Five Earth System Model (ESM) simulations are employed as benchmarks for plausible climate conditions, thereby mitigating biases stemming from any individual model. We focus on summer (JJA) drought and associated circulation variability, motivated both by the importance of warm-season hydroclimate for European drought impacts and by the fact that many tree-ring proxies most directly record warm-season conditions. EULMDA enables investigation of the spatiotemporal characteristics of the European climate variations over the past millennium and provides insights into the physical mechanisms driving hydroclimate variability. We hypothesize that employing this approach enables a robust assessment of the relative influence of atmospheric teleconnections on European summer droughts.

## Results and discussion

### Validation of EULMDA climate reconstruction

**Fig. 1:**
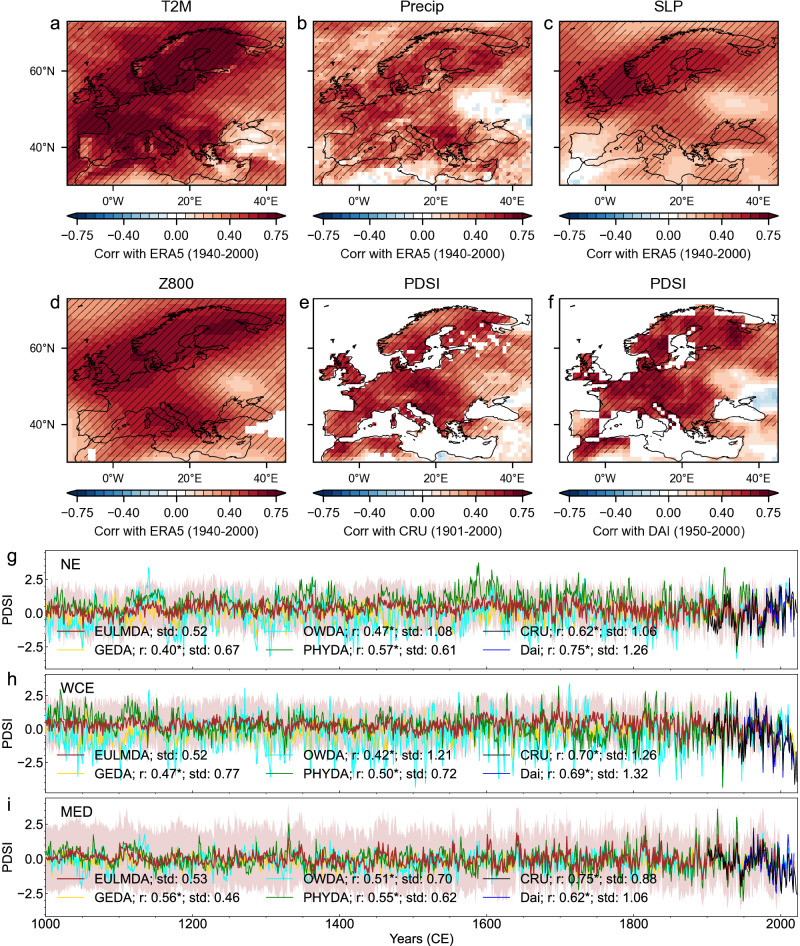
Skill evaluation of EULMDA. EULMDA is annually resolved, and targets June–August (JJA) means; all correlations use JJA-mean values. **a**–**d** Grid-point correlations between EULMDA and ERA5 for JJA near-surface air temperature (T2M; a), precipitation (Precip; b), sea level pressure (SLP; c) and 800-hPa geopotential height (Z800; d) anomalies during 1940–2000 CE. **e**,** f** Grid-point correlations of JJA PDSI anomaly between EULMDA and CRU (1901–2000 CE;** e**) and between EULMDA and Dai PDSI (1950–2000 CE; **f**). Shading denotes the correlation coefficient; hatched areas indicate statistical significance (*p* < 0.05). **g**–**i** Regional mean JJA PDSI anomaly time series for northern Europe (NE; **g**), west-central Europe (WCE; h) and the Mediterranean (MED;** i**), comparing EULMDA with instrument-based datasets (CRU and Dai PDSI) and existing statistical and DA-based reconstructions (OWDA, GEDA and PHYDA). Regional domains are shown in Supplementary Fig 1a. For each series, we report its correlation with EULMDA (r) and the standard deviation (std).

EULMDA is extensively validated against multiple instrumental datasets and a wide range of proxy-based reconstructions. It exhibits significant positive correlations with ERA5 for JJA near-surface air temperature, precipitation, sea level pressure and 800-hPa geopotential height (obtained by linear interpolation in pressure) across most of Europe and the Mediterranean during 1940–2000 CE (Fig. 1a–d). The mean squared error skill score further confirms this skill (Supplementary Fig. 2). The median of the correlation coefficient across reconstruction members using the different climate models is comparable to that of the ensemble mean (Supplementary Fig. 3), suggesting limited sensitivity to the choice of ESMs in our reconstructions. Despite known challenges in precipitation reconstruction^37^, EULMDA yields high precipitation correlations across most of Europe (Fig. 1b). Longer-term comparison with the 20CR reanalysis (1850–2000 CE) further supports the reliability of EULMDA (Supplementary Fig. 4). EULMDA-derived Palmer Drought Severity Index (PDSI) agrees well with CRU^38^ and Dai PDSI^39^, with 59% and 71% of land grid cells exhibiting correlations above 0.5 (Fig. 1e, f), and regional correlations exceeding 0.6 for Northern Europe (NE), Western-Central Europe (WCE), and the Mediterranean (MED; Fig. 1g–i; the definition of the three regions is shown in Supplementary Fig. 1a).

Summer large-scale circulation modes are also well captured. The JJA NAO index (see Methods for NAO calculation) correlates at *r* = 0.5* with instrumental observations over 1900–2000 CE (an asterisk indicates significance at *p* < 0.05; this convention is used throughout), and the reconstruction reproduces well the temperature, precipitation and pressure patterns associated to NAO (Supplementary Fig. 5). We also evaluated the JJA SCAND pattern over the European sector (20°–80°N, 40°W–12°E), which is defined here on a domain more centered over western Europe than in the canonical definition^40^, because of the focus of our study (see Methods for SCAND calculation). Despite this difference, the two indices are highly consistent (*r *= 0.74* over 1950–2024; Supplementary Fig. 6a), as both capture the characteristic Scandinavian high-pressure center. The European-sector SCAND index derived from EULMDA also agrees well with observations, showing a correlation of 0.70* with both the NCAR/NCEP- and ERA5-derived indices over 1950–2000 CE, and a correlation of 0.63* with the 20CR-derived indices over 1850–2000 CE. Its associated spatial climate signature is likewise consistent with observations (Supplementary Fig. 6b–m).

Further validation comes from comparisons with existing proxy-based reconstructions over the past millennium. EULMDA aligns closely with the summer temperature reconstruction by Luterbacher, et al. (2016)^41^, with a correlation of 0.80* over 1000–2000 CE (Supplementary Fig. 7a). Comparisons with the Old World Drought Atlas (OWDA^42^) and the Great Eurasian Drought Atlas (GEDA^43^) reveal substantial spatial agreement in hydroclimatic variations (Supplementary Fig. 7b, c) and significant PDSI correlations for the three subregions (NE, WCE and MED; Fig. 1g–i). EULMDA also compares well with two DA-based products, the Modern Era Reanalysis (ModE-RA^44^) and the Paleo Hydrodynamics Data Assimilation product (PHYDA^45^), showing broad spatial coherence for summer temperature, precipitation, pressure, and PDSI across Europe over the common periods (Supplementary Fig. 7d–h and Fig. 1g–i).

To provide additional independent validation, we compare EULMDA with an Alpine reconstruction^46^ that blends early instrumental measurements with documentary evidence. Over 1700–2000 CE, EULMDA temperature and precipitation correlate significantly (*p* < 0.05) with this reconstruction over the whole region covered (Supplementary Fig. 8a–b). Precipitation is compared with the Paris station record^22^ by extracting the nearest EULMDA grid cell (Supplementary Fig. 8c); the correlation over 1700–2000 CE is 0.42*, indicating that EULMDA captures a substantial fraction of interannual variability at this independent site.

Sensitivity tests on the number of proxies used (Supplementary Fig. 1c) suggest that the phase of reconstructed variability remains largely robust to differences in tree-ring coverage (Supplementary Fig. 9). By contrast, the amplitude is more sensitive and becomes increasingly damped in earlier centuries (Supplementary Fig. 10), partly reflecting limited proxy constraints^47^. The reconstruction ensemble mean, therefore, emphasizes the variability that is most robustly constrained by the proxy observations, whereas the ensemble spread in EULMDA quantifies the range of plausible variability (uncertainty).

In summary, although EULMDA tends to underestimate the amplitude of variations, especially in the first part of the reconstruction, it shows strong agreement with multiple extensively validated datasets for surface climate variables and circulation patterns. This consistency, achieved despite differences in methodology, selected observations (with only partial proxy overlap with some reconstructions), and processing approaches, underscores the robustness of the product. More statistical details on the evaluation of the reconstruction are provided in Supplementary Text S1.

### European drought modes over the past millennium

To investigate the spatial structure and the climate drivers of European drought variability over the past millennium, we decomposed the JJA PDSI into spatiotemporal modes of variability using empirical orthogonal function analysis. The first two leading modes account for 42.8% and 24.8% of the total variance, respectively. The first mode features a north–south dipole drought pattern (hereafter referred to as the NS mode), with negative PDSI anomalies (drier conditions) in northern Europe and positive anomalies (wetter conditions) in the south for its positive phase (Supplementary Fig. 11a). The second mode corresponds to a pan-European drought pattern (hereafter referred to as the PAN mode), featuring a nearly uniform wetting or drying signal across most of Europe (Supplementary Fig. 11b). To assess the sensitivity of these modes to period investigated, we repeated the analysis over two subperiods, 1000–1500 and 1000–1850 CE. Both subperiods yield NS- and PAN-like patterns consistent with the full-period results (Supplementary Fig. 11), indicating that the leading modes are temporally robust.

### North–south dipole drought pattern and its drivers

**Fig. 2:**
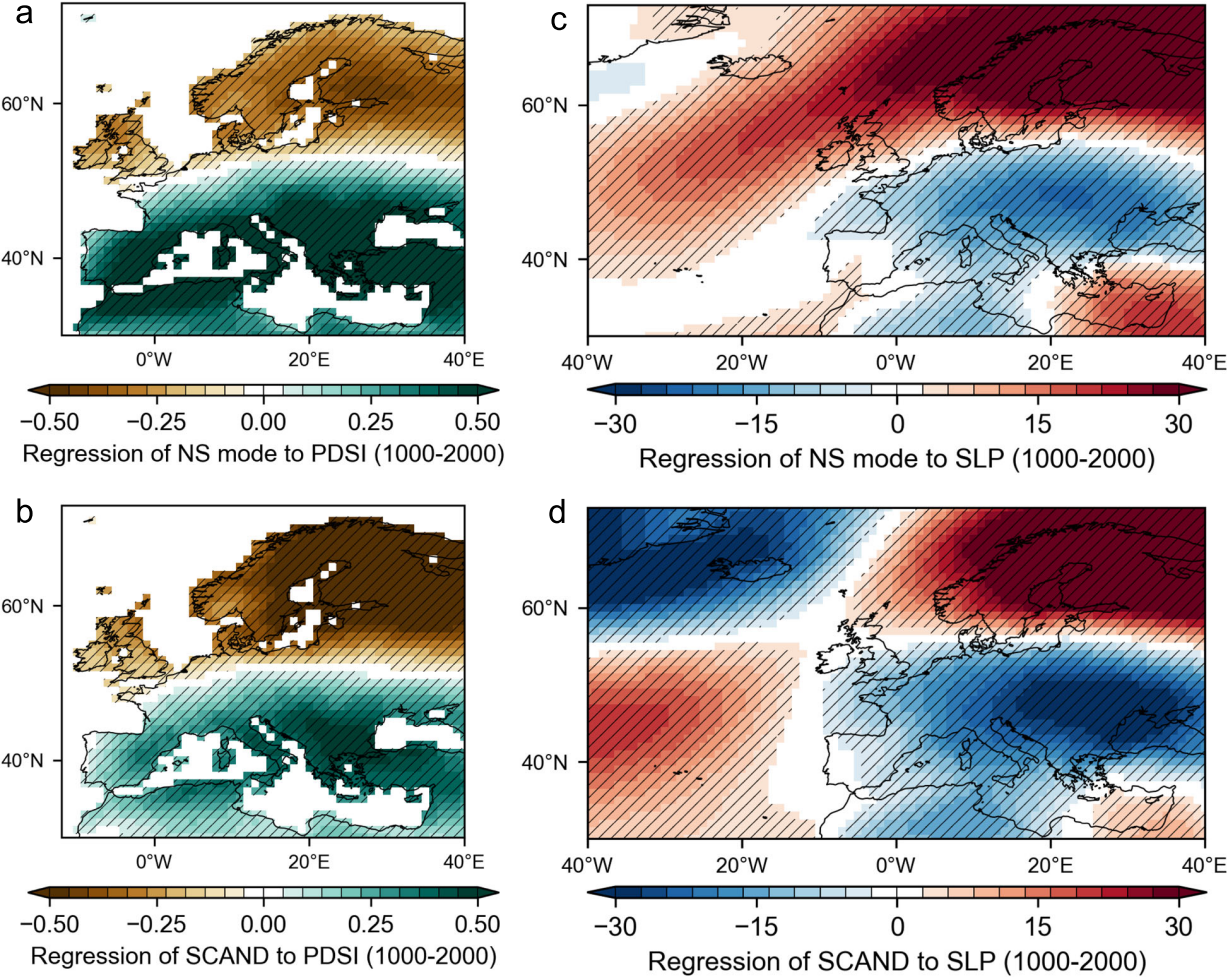
Comparison between the NS mode and JJA Scandinavian pattern (SCAND) over 1000–2000 CE. **a**,** b** Regression of PDSI onto the NS-mode principal component (PC; a) and the SCAND index (b). Positive (negative) values indicate wetter (drier) conditions. **c**,** d** Regression of sea-level pressure (SLP; Pa) onto the NS-mode PC (**c**) and the SCAND index (**d**). Hatched areas indicate statistical significance (*p* < 0.05). All regressions are computed using unfiltered annual JJA anomalies.

The NS mode is closely associated with the SCAND atmospheric circulation pattern over the European sector. Over the past millennium, SCAND exhibits a strong temporal correlation with the NS mode (*r* = 0.78*), and their associated sea-level pressure and PDSI anomaly fields show high spatial correspondence (*r* = 0.85* for pressure and 0.97* for PDSI; Fig. 2). For robustness, we also examined geopotential heights at 800hPa and 500hPa over Europe, which reproduce the same dipole structure as sea level pressure (Supplementary Fig. 12a–d). During positive SCAND phases, a persistent high-pressure anomaly develops over Scandinavia, leading to reduced precipitation and elevated temperatures in northern Europe (Supplementary Fig. 12e–h). These conditions contribute to pronounced negative PDSI anomalies in the north (*r* = – 0.90* between SCAND and regional averaged PDSI of NE), while simultaneously shifting the storm track southward, which favors wetter conditions in the south (*r* = 0.53* between SCAND and regional averaged PDSI of MED).

Because the summer NAO can also induce a north–south dipole in precipitation (e.g., ref. ^48^, Supplementary Fig. 5b, e), we evaluated whether NAO variability contributes to the NS mode. To isolate the dynamical NAO signal (given that the summer NAO covaries with European mean surface temperature), we removed the covariance with summer temperature from the NAO index. The resulting residual NAO yields a dipole-like PDSI response, as shown in the “Pan-European drought pattern and its drivers” section, but correlates only weakly with the NS-mode index (*r* = 0.22*), indicating a comparatively weak dynamical contribution.

The NS pattern is consistent with observed multidecadal hydroclimate changes since the mid-20^th^ century, with prominent wetting and drying over northern and southern Europe^49,50^ (Supplementary Fig. 13). It also broadly aligns with projected European hydroclimate responses to climate change^51,52^. Previous studies have suggested that this regional north-south divergence may be driven by increased precipitation in northern Europe^53,54^ and warming-induced evapotranspiration increases in southern Europe^19^. Our results imply a possible atmospheric dynamical origin behind this pattern, motivating a closer examination of the role of SCAND in European summer hydroclimate variability and trends.

### Role of the Scandinavian pattern in past and present summer European hydroclimates

**Fig. 3:**
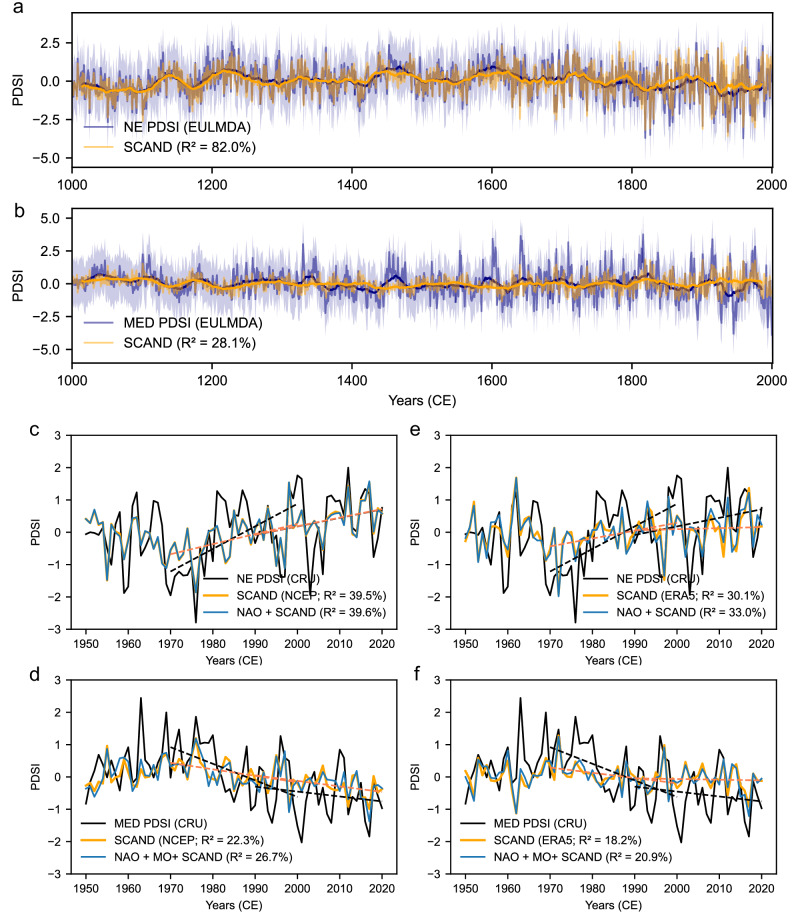
Influence of the SCAND on JJA PDSI in northern Europe and the Mediterranean over the past millennium and recent decades. **a**,** b** EULMDA results for 1000–2000 CE showing reconstructed regional-mean JJA PDSI anomaly (blue; ensemble mean with uncertainty shading) for northern Europe (NE;** a**) and the Mediterranean (MED;** b**), together with PDSI anomaly estimated from linear regression on the SCAND index (orange). Thick curves show 31-year running means, and R² indicates the variance explained. **c**,** d** Instrumental-period results over 1950–2020 CE, comparing CRU regional-mean JJA PDSI anomaly (black) with PDSI anomaly estimated from regression on SCAND alone (orange) and from multiple-index regression (blue) using circulation indices from NCEP/NCAR: SCAND + NAO for NE (c) and SCAND + NAO + MO for MED (d). Dashed lines indicate linear trends for the periods 1970–2000 CE and 1990–2020 CE; corresponding trend estimates and significance are reported in the text. **e**,** f** As in (**c**, **d**), but with the SCAND index derived from ERA5.

Building on the dynamical link between SCAND and NS mode, we quantify SCAND’s imprint on regional drought variability. In EULMDA, SCAND alone explains most of the interannual PDSI variance in northern European (NE; 82.1%) and a substantial fraction in the Mediterranean (MED; 28.1%) over 1000–2000 CE (Fig. 3a, b). SCAND is also significantly correlated with summer precipitation (*r *= – 0.73* for NE; *r* = 0.60* for MED), indicating that the same circulation mode modulates hydroclimate anomalies in both regions.

These reconstruction-based relationships are also evident in the modern observational record. Using NCEP/NCAR reanalysis over 1950–2020 CE, the SCAND index is strongly correlated with summer precipitation (*r *= – 0.63* for NE; *r* = 0.54* for MED). Stepwise regression further shows that SCAND explains 39.5% of northern European PDSI variance and 22.3% of the Mediterranean PDSI variance (Fig. 3c, d). Summer NAO explains less variance than SCAND for northern Europe, and for the Mediterranean, adding the summer Mediterranean Oscillation (MO) alongside the NAO increases explained variance by only 4.4%, underscoring SCAND’s dominant role; Results are consistent when using the ERA5 reanalysis (Fig. 3e, f).

Over the Mediterranean, summer precipitation is relatively limited across large parts of the region, and summer drought has therefore often been linked to cold-season to spring precipitation and associated atmospheric dynamics^18,55^. Consistent with this mechanism, EULMDA shows a significant relationship between spring SCAND variability and subsequent summer PDSI in the Mediterranean (Supplementary Fig. 14). We further examine whether summer SCAND can also modulate Mediterranean summer drought through its imprint on temperature. SCAND is significantly correlated with Mediterranean JJA temperature (*r *= – 0.40* over 1000–2000 CE in EULMDA; *r *= – 0.72* over 1950–2020 CE in NCEP/NCAR). Using partial correlations, we find that in EULMDA (1000–2000 CE), the SCAND–PDSI correlation generally remains significant after controlling for either summer precipitation or temperature, suggesting contributions from both pathways (Supplementary Fig. 15a–c). In instrument-based datasets (1950–2020 CE), temperature mediation is more prominent: the SCAND–PDSI link remains significant when controlling precipitation but weakens substantially when controlling temperature (Supplementary Fig. 15d–i).

A recent observational study, based on the most comprehensive station-based Mediterranean precipitation dataset currently available, has also highlighted the importance of atmospheric circulation for Mediterranean hydroclimate variability^56^. Vicente-Serrano et al. (2025) show that NAO and MO together explain a significant fraction of precipitation variability in other seasons but only ~ 12.8% in summer (1931–2020 CE). Notably, the SCAND index was not included in their analyses, thereby omitting a potentially powerful explanatory factor for summer. Using the same dataset, we find that summer SCAND explains 37.9% of Mediterranean precipitation variance during 1950–2020 CE (Supplementary Fig. 16), suggesting that incorporating SCAND may offer additional insight into understanding summer Mediterranean hydroclimate variability in both the modern period and the past.

SCAND also modulates European multidecadal drought trends. Over the past millennium, the SCAND index and the NS-mode index exhibit significant temporal coherence in 31-year running means (*r* = 0.74*, with significance assessed accounting for reduced degrees of freedom due to smoothing; Supplementary Fig. 17a). Consistently, multidecadal mean PDSI variability in northern Europe and the Mediterranean also covaries with SCAND (*r *= – 0.88* for NE and *r* = 0.43* for MED in 31-year running means; Fig. 3a, b). SCAND and the NS mode also show coherent multidecadal trend variations (*r *= 0.76* for 31-year moving trends; *r* = 0.71* for 51-year moving trends; Supplementary Fig. 17b, c).

Since the 1970s, SCAND exhibits a significant negative trend in EULMDA ( − 0.64 per decade over 1970–2000 CE, *p* < 0.05; Supplementary Fig. 18a). Negative SCAND phases tend to produce lower-than-normal pressure over northern Europe, favoring enhanced rainfall and wetter conditions, while higher pressure over southern Europe suppresses precipitation and increases temperature, thereby enhancing drying (Supplementary Fig. 13). Consistent with this dynamical picture, the NS-mode index and the corresponding north-south precipitation gradient index (Methods) both show significant negative trends over 1970–2000 CE (– 0.87 and – 0.81 standardized units per decade, respectively; both *p* < 0.05; Supplementary Fig. 18b, c), of which SCAND accounts for 70% and 76%. This negative trend corresponds to wetting in northern European and drying in the Mediterranean (Supplementary Fig. 18d–g). In northern Europe, precipitation increases by + 0.09 mm day^−1^ decade^−1^ (*p* < 0.05) and PDSI by + 0.44 units decade^−1^ (*p* < 0.05), with SCAND accounting for 96% and 126% of the respective trends. By contrast, in the Mediterranean, precipitation decreases by − 0.07 mm day^−1^ decade^−1^ (*p* < 0.05) and PDSI by − 0.85 units decade^−1^ (*p* < 0.05), with SCAND-related contributions of 84% and 67%.

Instrument-based data provide a consistent picture over 1970–2000 CE (Supplementary Fig. 18h–l). Furthermore, we also examine changes over the most recent three decades (1990–2020 CE). Compared to 1970–2000 CE, the negative SCAND trend weakens slightly (− 0.38 per decade, *p* < 0.05; Supplementary Fig. 18h). Consistent with this weakening, Mediterranean precipitation and PDSI move back toward their mean state, with trends of + 0.01 mm day^−1^ decade^−1^ (*p* = 0.74) and − 0.17 units decade^−1^ (*p* = 0.36), respectively (Supplementary Fig. 18k, l). The northern European PDSI wetting trend also weakens (+ 0.25 units decade^−1^, *p* = 0.20; Supplementary Fig. 18j). In contrast, northern European precipitation increases more strongly in the regional mean over 1990–2020 ( + 0.15 mm day^−1^ decade^−1^, *p* < 0.05; Supplementary Fig. 18i), largely caused by localized wetting near the English Channel region^21^. Spatial patterns further indicate that northern European wetting is more spatially coherent over 1970–2000 than over 1990–2020 (Supplementary Fig. 13a–d).

Finally, to assess whether the recent SCAND trend and associated north-south divergence are unprecedented, we place these changes in the context of the past millennium. The negative SCAND and NS trends over the recent 30-year interval (1970–2000 CE) appear unusually strong compared to the past millennium, whereas the longer 50-year trends (1950–2000 CE) do not (Supplementary Fig. 17b, c). Together with the weakening of the negative SCAND trend during 1990–2020 (Supplementary Fig. 18h), these results suggest that recent SCAND and NS-mode variations remain within the range of internal variability and explaining them does not require a substantial contribution from anthropogenic forcing. Regionally, northern Europe wetting during 1970–2000 CE falls within the past-millennium range, whereas Mediterranean drying during the same interval appears particularly pronounced in EULMDA (Supplementary Fig. 17b). However, given the underestimation of variance in earlier centuries in our mean reconstruction, this drying may not in fact have been truly unprecedented, and the subsequent weaker drying trend during 1990–2020 CE is even less likely to be exceptional. Consistent with previous studies (e.g., ref. ^56^), these results suggest that recent multidecadal Mediterranean drought trends may largely reflect internal variability, which might have masked the emerging influence of anthropogenic forcing.

### Pan-European drought pattern and its drivers

**Fig. 4:**
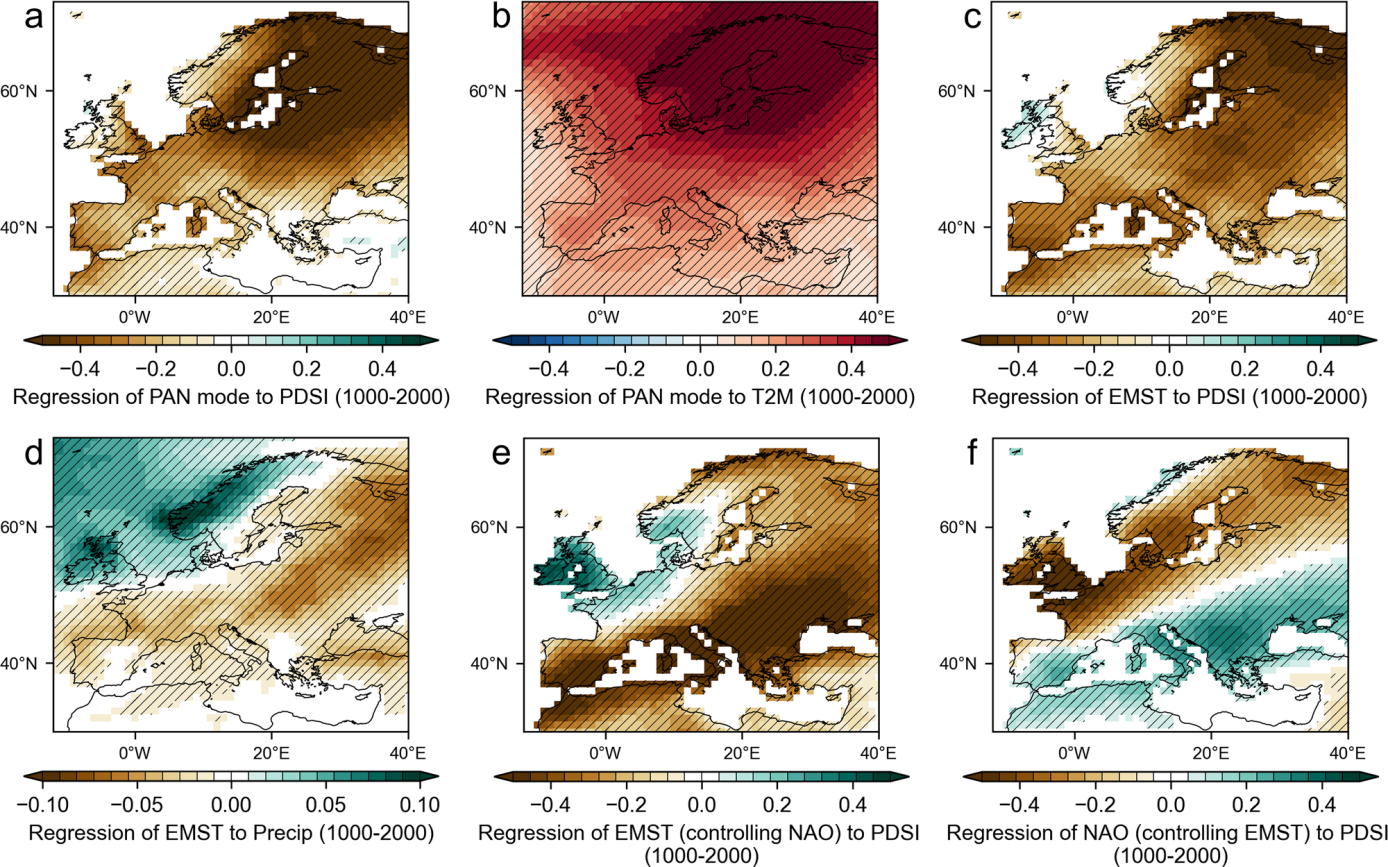
PAN mode and its links to JJA European mean surface temperature (EMST) and NAO over 1000–2000 CE. **a**, **b** Regression of PDSI (**a**) and near-surface temperature (T2M; K; **b**) anomalies onto the PAN-mode principal component. **c**,** d** Regression of PDSI (c) and precipitation (Precip; mm d^-1^; d) anomalies onto EMST. **e** Partial regression of PDSI onto EMST after removing the linear influence of NAO (i.e., the EMST residual). **f** Partial regression of PDSI onto the NAO after removing the linear influence of EMST (i.e., the NAO residual). Hatched areas indicate statistical significance (*p* < 0.05). All regressions are computed using unfiltered annual JJA anomalies.

The PAN mode reflects a continent-wide pattern of drought variability (Fig. 4a) and is mainly driven by summer temperature changes. It shows a strong negative correlation with European Mean Surface Temperature (EMST;* r *= – 0.93*) over 1000–2000 CE, indicating a general drying response to European summer warming. Regressing near-surface temperature onto the PAN-mode index yields spatially coherent temperature anomalies across most of Europe, with the largest amplitudes over northern Europe (Fig. 4b). The PAN mode and EMST are also associated with highly similar geopotential-height anomaly structures at both 800 and 500hPa (spatial *r* = 0.97* and 0.98*; Supplementary Fig. 19).

Regressing PDSI onto EMST reveals a widespread drying signal across Europe, except for a localized wetting anomaly over the northern British Isles (Fig. 4c), caused by the increased precipitation during periods of high EMST there (Fig. 4d). Importantly, the summer NAO also correlates well with EMST, as derived from instrument-based reanalysis (20CR; *r* = 0.44* over 1900–2000 CE) and from the EULMDA reconstruction (*r* = 0.78* over 1000–2000 CE). Additional analyses indicate that removing NAO’s contribution from EMST amplifies the wetting signal over the English Channel region (Fig. 4e). In fact, positive summer NAO alone could lead to reduced rainfall over western Europe, southwestern Scandinavia, and the British Isles, creating a regional drying center around the English Channel region^29^ (Fig. 4f). Summer NAO thus combines with EMST leading to a relatively homogeneous PAN mode throughout Europe. Nevertheless, on average across the European continent, EMST (removing the covariance with NAO) is the dominant driver of the PAN mode, explaining 44% of the variance. In contrast, the NAO (removing the covariance with EMST) adds a secondary and regionally confined influence, accounting for 20% of the variance.

The spatial structure in the EMST-hydroclimate relationship has been noted in previous studies^7,57^. For instance, proxy-based and model studies find that on decadal to multi-decadal timescales, higher temperatures are associated with drier conditions in southern Europe but with wetter conditions in parts of northern Europe^57^. From a thermodynamic perspective, as temperature rises, the atmospheric moisture-holding capacity increases according to the Clausius–Clapeyron relation. However, moisture availability becomes a limiting factor in southern and central Europe, where soil moisture limitations constrain evapotranspiration, favoring drying under warming conditions^19,58^. By contrast, in the English Channel region/northeastern Europe, the persistent availability of moisture from the North Atlantic explains that higher temperatures may favor enhanced precipitation rather than drying^59^.

EMST responds strongly to volcanic eruptions over the past millennium^60^. Consistent with this, the PAN mode shows significant wet anomalies (*p* < 0.05) following Northern Hemisphere and tropical volcanic eruptions^61^ (*n* = 23), in line with post-volcanic cooling and wetter European summers^62^ (Supplementary Fig. 20a, b and Supplementary Table. 2). In contrast, since ~ 1950, the PAN mode exhibits a marked drying trend (– 0.45 units decade^−1^; *p* < 0.05 over 1960–2000 CE), alongside a concurrent increase in EMST (0.57 °C decade^−1^; *p* < 0.05). A linear regression suggests that the EMST trend alone would imply a PAN drying trend of comparable magnitude (∼ 120% of the observed PAN trend), indicating that recent PAN drying is closely linked to warming (Supplementary Fig. 20a, c).

### Relative contributions of large-scale climate drivers

To assess the relative importance of these large-scale climate drivers, we quantified to what extent the reconstructed summer PDSI variability can be explained by SCAND, EMST (after controlling for NAO), and NAO (after controlling for EMST) over the past millennium. A multiple linear regression reveals that these three factors together account for 62.2% of the total PDSI variance across Europe from 1000–2000 CE. Among them, SCAND explains the largest fraction (32.1%), underscoring its critical role in driving European drought variability. Its influence extends across both northern and southern Europe, controlling the spatial contrast between dry and wet anomalies in these two regions (Supplementary Fig. 21a). EMST contributes 28.8% of the variance, with the strongest influence in the Iberian Peninsula, while the EMST-PDSI relationship is weaker in the English Channel region (Supplementary Fig. 21b). In contrast, NAO accounts for 4% of the total variance, showing a more localized impact around western Europe and the British Isles (Supplementary Fig. 21c).

Our results suggest that the weaker EMST–PDSI relationship around the English Channel likely reflects a larger role of dynamic processes (e.g., the NAO) in modulating summer rainfall there. This interpretation is consistent with recent mechanistic studies using climate models^18,63–65^, which suggest that large-scale circulation anomalies have a substantial impact on summer rainfall in northwestern Europe, whereas local thermodynamic factors (e.g., soil moisture feedbacks) contribute more to the drying signal in southern Europe. However, because EULMDA does not directly constrain land-surface states or fluxes, it cannot provide an independent quantification of soil moisture feedback strength.

Overall, these three factors explain most of the PDSI variability at the European scale (Supplementary Fig. 21d), but their contributions are weaker in central Europe (Supplementary Fig. 21e). Central Europe is a transition area in which additional dynamic processes, such as jet stream variability^31^ and land-atmosphere interactions^17,66^, may play a more significant role.

### Insights into the drivers of European summer hydroclimate variability

Our reconstruction (EULMDA) demonstrates that European summer drought variability is mainly organized around two spatial modes whose drivers are physically distinct: a dynamically driven north–south dipole linked to the SCAND pattern, and a thermodynamically driven pan-European mode tied to summer warming. These processes can be interconnected and sometimes coupled^7^, making it challenging to disentangle the explicit role of temperature versus circulation, or to establish links of causality between them, based on only the data assimilation method. Nevertheless, our results imply that the synchronization and competition of these drivers ultimately determine hydroclimatic consequences. In the case of the Mediterranean, both a negative SCAND pattern (dynamic driver) and warming temperatures (thermodynamic driver) push toward drying in the summer over the past century.

Our millennial-length perspective suggests that north–south dipole decadal variability and Mediterranean drying of the late-20^th^ and early-21^st^ centuries, which coincided with a significant negative SCAND trend, are not unprecedented. This implies that in recent decades, anthropogenic forcing has not consistently outweighed the influence of internal variability in shaping regional drought patterns. By contrast, long-term projections of continent-wide drying may reflect the growing dominance of thermodynamic warming, consistent with the expected response to greenhouse gas forcing^51^.

These findings highlight the need to evaluate and project the co-evolution of large-scale circulation patterns, anthropogenic forcings, and hydroclimate in climate models. Improving the representation of SCAND variability and its interaction with warming will be critical for reliable near-term drought predictions and long-term risk assessments for Europe. Such improvements are also relevant for assessing socio-ecological impacts, as hot–dry summers are tightly linked to agricultural drought and to fire-weather conditions conducive to large wildfires across Europe^67,68^.

## Methods

### Paleoclimate data assimilation

Paleoclimate Data Assimilation (DA) optimally combines climate models with proxy observations to estimate historical climate variations^44,45,69^. DA allows spreading the information from the proxy locations into space and variables that are not assimilated, by relying on the covariance between variables provided by the climate model^70^. DA-based reconstructions thus guarantee dynamical consistency among all the reconstructed variables.

Our framework follows previous offline particle-filter implementations^71,72^ and consists of four primary components: (i) climate model outputs used as a prior ensemble that samples possible climate states and their covariances; (ii) proxy observations that provide the temporal information for the reconstruction; (iii) Proxy System Models (PSMs) that relate the model variables to proxy observations, and (iv) a DA algorithm, here a particle filter^73^.

In a Bayesian formulation^73,74^, the prior information is updated by observations to obtain a posterior estimate. In our approach, the prior is the state of the climate system (called particle) ${{\bf{\psi }}}$, provided by the climate model simulations. The conditional probability of proxy observations ${{\bf{d}}}$ given the prior (i.e., the likelihood) is assumed Gaussian:

1 $$p\left({{\bf{d}}} | {{\bf{\psi }}}\right)={K}^{-1}\exp \left[-\frac{1}{2}{\left({{\bf{d}}}-H\left({{\bf{\psi }}}\right)\right)}^{T}{C}^{-1}({{\bf{d}}}-H\left({{\bf{\psi }}}\right))\right]$$

where ${K}^{-1}$ is a normalization constant, $H$ is the forward operator that maps the model variables into proxy observation space (i.e., the Proxy System Models), and $C$ is the observation-error covariance, which is assumed diagonal here.

We apply an offline DA approach, meaning that the same prior ensemble is used at each assimilation step, and the model does not provide temporal information (in contrast to online DA that propagates the prior forward in time using the previous-step posterior). This assumption is appropriate for annual atmospheric variability because of its relatively small autocorrelation in time^75,76^, and has been widely used in reconstructions focusing on historical hydroclimate variability^44,45,72,77,78^. Because we target interannual hydroclimate variability, the assimilation time step is one year: for each reconstructed year, the prior ensemble is updated using the proxy information to obtain the posterior. In our implementation, the state vector contains monthly climate fields within each year, but these fields are updated only in the annual analysis step, using proxy constraints that are defined on annual or seasonal means by the PSMs (see “Proxy System Models” section). As a result, seasonal means of interest (e.g., JJA and MAMJJAS, as well as other seasons) can be derived from the same DA output.

We represent the prior probability density function (pdf) by a discrete (non-Gaussian) ensemble:

2 $$p\left({{\bf{\psi }}}\right)=\,\frac{1}{N}\mathop{\sum }_{i=1}^{N}\delta ({{\bf{\psi }}}-{{{\bf{\psi }}}}_{i}),$$

where ${{{\bf{\psi }}}}_{i}$ is the climate state of particle $i$, $N$ is the number of particles, and $\delta$ denotes a kernel density. Each particle initially has equal weight in the prior. After evaluating the likelihood for each particle, we assign each particle a weight ${w}_{i}$ that is proportional to $p\left({{\bf{d}}},|,{{{\bf{\psi }}}}_{i}\right)$, yielding the posterior distribution:

3 $$p\left({{\bf{\psi }}} | {{\bf{d}}}\right)=\,\mathop{\sum }_{i=1}^{N}{w}_{i}\delta ({{\bf{\psi }}}-{{{\bf{\psi }}}}_{i}),$$

with weights

4 $${w}_{i}=\frac{p\left({{\bf{d}}} | {{{\bf{\psi }}}}_{i}\right)}{{\sum }_{j=1}^{N}p\left({{\bf{d}}} | {{{\bf{\psi }}}}_{j}\right)}.$$

The mean reconstruction, hereafter referred to as the reconstruction, is taken as the posterior weighted mean of the particles, and uncertainty is quantified as the posterior weighted standard deviation. In ensemble-based reconstructions, the posterior mean tends to exhibit reduced variance, reflecting a bias–variance trade-off (e.g., refs. ^47,79^), especially when proxy constraints are sparse or noisy and for less spatially coherent variables such as precipitation. Importantly, this does not imply that variability is absent from the posterior ensemble; rather, variance is retained in individual particles and the posterior spread, while the ensemble mean emphasizes the component of variability that is most robustly constrained by the proxy information.

### Earth System Model simulations

We use simulations performed with five different Earth System Models (ESMs), including varying simulation durations and ensemble member sizes, to help mitigate the potential influence of biases in any one particular ESM (Supplementary Table. 1). The first model is CESM1-LME (Community Earth System Model Version 1-Last Millennium Ensemble), which provides 12 simulations, forced by solar variability, volcanic eruptions, land use changes, greenhouse gases, and orbital changes^80^, spanning the 850–1850 period. Three model simulations are part of the PMIP4-CMIP6 *past1000* simulations^81^, using ACCESS-ESM1-5 (Australian Community Climate and Earth System Simulator-Earth System Model version 1.5), MIROC-ES2L (Model for Interdisciplinary Research on Climate-Earth System version 2 for Long-term simulations), and MRI-ESM2-0 (Meteorological Research Institute Earth System Model version 2.0). Each simulation covers the period from 850 to 1849 CE and provides a single-member simulation, using the default PMIP4-CMIP6 forcings^82^. CESM2-LE (Community Earth System Model version 2-Large Ensemble) simulations covering the historical period (1850–2014) are also included, as they provide a very large number of ensemble members (100 members)^83^. Those simulations follow the historical forcing protocols provided by CMIP6 (Eyring et al., 2016), although with differences in the representation of biomass burning in 50 of the 100 ensemble members. All simulations were linearly interpolated onto a grid of 1° resolution.

We use the climate anomalies from the model simulations (relative to each model’s climatology), thus removing each model’s mean bias. Climate model states (particles) are sampled each year, so the number of particles used in each experiment corresponds to the product of the total simulation length (in years) and the size of the ensemble (Supplementary Table. 1). No pre-assimilation ensemble averaging is applied: each year of each member is included in the prior as an individual particle. All these particles constitute the initial estimate of the distribution of the climate state (prior). We performed separate reconstructions using each model prior, thereby generating a set of multi-model-ensemble reconstructions. The final EULMDA product is computed as the mean of these five members.

### Tree-ring records

We compiled tree-ring records for our DA-based reconstruction due to their annual resolution, demonstrated sensitivity to local climate variability, and broad spatial coverage across Europe^84,85^. These records were sourced from different databases and previous extensive studies, including the Northern Hemisphere Tree-Ring Network Development (N-TREND)^86^, version 2.0.0 of the global PAGES 2k temperature proxy data collection^87^, 45 tree-ring cellulose δ^18^O chronologies across Europe synthetized by ref. ^88^, and some additional records not included in these data bases^89–92^. These records represent a range of variables: ring width, ring density, oxygen isotope and radial-cell-wall thickness (Supplementary Fig. 1a). We retained only records with significant correlations (*p* < 0.05) with observed temperature or precipitation during the annual, summer (JJA) or growing season (MAMJJAS) over the period 1901–2010 CE. Instrumental climate observations used for screening were taken from the Global Meteorological Forcing Dataset for land surface modeling v2 (GMF^93^).

To match model grid and reduce non-climatic noise, we merged selected tree-ring chronologies within grid cells at 1° grid. Before averaging, we required that the candidate records within a cell be positively correlated with each other; when correlations were not significant, we removed the record with the weaker correlation to instrumental data. The final proxy dataset includes 106 merged tree-ring series, each significantly correlated with at least one climate variable at the annual, summer or growing season. Although the number of records begin to decline before 1900, nearly half of the records (*n* = 53) cover the last 400 years, and approximately one-third (*n* = 30) cover the last 700 years (Supplementary Fig. 1c). The spatial distribution of records starting from 1306 CE and 1600 CE onwards is shown in Supplementary Fig. 1d, e.

### Proxy System Models (PSMs)

PSMs are needed for comparing tree-ring records and climate variables simulated in models. Following common practice in DA reconstructions^45,77^, we build a statistical PSM for each proxy series using seasonally aggregated climate predictors. For each record, the regressions are calibrated using climate variables averaged over one of three seasonal windows, annual (ANN), June–August (JJA), or March–September (MAMJJAS). Two types of regression are introduced, one is based on a univariate model considering either temperature or precipitation, the other is a bivariate model considering both temperature and precipitation. To account for different climate sensitivities across tree-ring sites, all the possible combinations of different climate variables and seasons were tested, leading to 15 candidate PSMs (3 temperature-only, 3 precipitation-only, and 9 bivariate models). We used the ordinary least squares method to estimate the parameters of the regression equations.

Calibration and validation were performed over 1901–2000 CE using the GMF data, interpolated to a grid of 1° resolution and excluding the oceanic grid cells. We used 60% of the data for calibration and the remaining 40% for validation. Given that the selection of calibration and validation periods possibly influences the regression results, we repeated the procedure 1000 times with different calibration and validation periods, providing an estimate of the associated uncertainty. Finally, the climate variable(s) represented by each record was identified according to the PSM with the lowest Bayesian information criterion (BIC) value among the 15 PSMs (Schwarz 1978). After this evaluation, most records are associated with summer variables. 33 tree-ring records are interpreted as related to temperature and thus compared to the near-surface temperature in the climate model, 54 records are interpreted as related to precipitation, and 19 records are interpreted as related to both temperature and precipitation (Supplementary Fig. 1b).

In addition, DA requires an observation error for each proxy, which enters the likelihood calculation and determines how strongly each record constrains the prior^94^. Records with low errors provide stronger constraints on model simulations, thus receiving larger weight in the DA compared to records with higher errors. Here, observation errors are determined by PSMs. The PSM error is quantified using the standard deviation of the residuals over the validation periods for 1000 ensemble members, encompassing both representativeness error (from site-grid comparisons) and errors related to the skill of the PSM^72^. The uncertainty due to the choice of calibration and validation periods is considered by calculating the standard deviation of the PSM error across the 1000 ensemble members. Consequently, the observation error of each of the record is equal to the sum of the PSM error and the error related to the uncertainty in calibration and validation. In the DA experiments, to allow potential underestimation of the errors due to factors not directly accounted for in our methodology, we inflated the observational errors by 50% (i.e., multiplied by 1.5), following Dalaiden et al. (2021)^72^.

### Evaluation for EULMDA

We evaluate the EULMDA reconstruction against both instrumental datasets and existing reconstructions. Two instrument-based reanalysis products are used to evaluate near-surface temperature, precipitation, sea-level pressure and 800-hPa geopotential height: ECMWF Reanalysis v5 (ERA5) ^95^ for the period 1940–2000 CE, and NOAA-CIRES-DOE Twentieth Century Reanalysis v3 (20CR) ^96^ over 1850–2000 CE. The PDSI is assessed against two products derived from instrumental observations: Climatic Research Unit gridded Time Series v4.07 (CRU TS) ^97^ over 1901–2000 CE and the Dai PDSI^39^ over 1950–2000 CE. To quantify reconstruction skill, we compute the Pearson correlation coefficient (r) and the Mean Square Error Skill Score (MSESS). MSESS measures the improvement in mean square error relative to a climatological reference forecast^98^, defined here as the mean of each instrument-based product over the evaluation period. By definition, MSESS > 0 indicates better performance than climatology, MSESS = 0 indicates no improvement over climatology, and MSESS = 1 corresponds to a perfect reconstruction. We evaluate the skill over the overlapping period between EULMDA and each instrumental product for JJA and MAMJJAS. Skill is generally higher for JJA; therefore, all main analyses are based on JJA anomalies, unless otherwise noted.

We further compare EULMDA with several existing reconstructions. European mean surface temperature (EMST) is evaluated against a proxy-based European JJA temperature reconstruction since 138 BCE based on Bayesian hierarchical modeling and composite-plus-scaling^41^. Spatial temperature fields are compared with two DA-based reconstructions: the Modern Era Reanalysis (ModE-RA)^44^, which spans 1421–2008 CE and integrates ECHAM6 simulations with natural proxies and documentary data in earlier periods and instrumental data from the 17th century onward; and the Paleo Hydrodynamics Data Assimilation product (PHYDA)^45^, which covers 1000–2000 CE and assimilates multiple paleo-proxy records using CESM-LME. For precipitation and sea-level pressure fields, comparisons are made with ModE-RA. For PDSI, both fields and regional time series are compared with PHYDA as well as with two tree-ring-based reconstructions: Old World Drought Atlas (OWDA)^42^, which provides annual summer wetness and dryness maps over Europe and the Mediterranean during the Common Era using a point-by-point regression method; and the Great Eurasian Drought Atlas (GEDA)^43^, which builds upon and expands previous PDSI reconstructions including the OWDA, the Monsoon Asia Drought Atlas^99^, and the European Russia Drought Atlas^100^.

For an independent validation, we assess reconstructed temperature and precipitation over 1700–2000 CE against the Alpine reconstruction, which blends early instrumental measurements with documentary evidence^46^. We also compare reconstructed JJA precipitation with the Paris station series (48°52’N, 2°20’E) ^22^ using the nearest EULMDA grid cell.

### PDSI calculation

Drought is quantified using the Palmer Drought Severity Index (PDSI), a common dimensionless hydroclimate metric whose negative (positive) values are indicative of moisture deficit (surplus)^39^. To reconstruct PDSI over the past millennium, PDSI values are first calculated from each climate model (prior). Potential evapotranspiration is estimated using the Thornthwaite equation and monthly climate model output of 2-m air temperature. The computations of PDSI were carried out using Python code from the climateindices package^101^, which produces the self-calibrating PDSI. Once PDSI’s prior is calculated utilizing each model prior, it can be included in the data assimilation to calculate proxy-informed PDSI values over the past millennium.

### Scandinavian pattern and North Atlantic Oscillation index

The summer Scandinavian pattern (SCAND) is derived from rotated empirical orthogonal function (REOF) analysis of JJA mean 300-hPa geopotential height (Z300) anomalies over 20°–80°N, 12°W–40°E, encompassing the European sector. REOF is used to isolate statistically independent circulation patterns^34,40^. SCAND is identified by the presence of a distinct high-pressure center over the Scandinavian Peninsula, consistent with its canonical structure. In EULMDA (1000–2000 CE) and ERA5 (1940–2024 CE), SCAND emerges as the third leading mode, explaining 19.4% and 12.8% of the variance, respectively; in NCEP/NCAR (1948–2024 CE), it appears as the second mode, explaining 20.8%; and in 20CR (1806–2015 CE), it appears as the first mode, explaining 35.9%. The associated high-pressure center is clearly evident in the sea-level pressure fields (Supplementary Fig. 6d, g, j, m).

The NAO index is computed from the EULMDA reconstruction as the first EOF mode of JJA sea-level pressure anomalies over the Atlantic-European domain (90°W–40°E, 20°N–80°N), accounting for 44.7% of the variance. This definition and spatial domain follow established conventions^28^. For validation, we use the Hurrell PC-based JJA NAO index as an observational reference.

### Regression and covariance analysis

To relate European hydroclimate fields to large-scale climate indices, we compute linear regression maps of time-dependent spatial fields (PDSI, precipitation, temperature and sea-level pressure) onto scalar indices, including the NS and PAN modes, EMST, and the SCAND and NAO indices (Figs. 2 and 4). All regressions are performed on JJA anomalies. The indices are standardized, so the regression patterns can be interpreted as the mean spatial anomaly associated with a one-standard-deviation change in the index.

Although the maps are obtained by regressing the field onto the index, they can equivalently be interpreted in the inverse sense, i.e., as spatial patterns that can be used to predict the index from the field. This is mathematically equivalent to a one-dimensional maximum covariance (or singular value) analysis between the field and the index^102^. In the special case where the index is a principal component of the field itself (e.g., Figures 2a and 4a), the regression pattern is proportional to the corresponding Empirical Orthogonal Function loading pattern.

Furthermore, we quantify how trends in driver indices contribute to trends in hydroclimate fields (PDSI and precipitation). Specifically, for a given field $X\left(r,t\right)$ and an index $I\left(t\right)$ (e.g., SCAND index), we estimate the regression coefficient $\beta \left(r\right)$ from

5 $$X\left(r,t\right)=\beta \left(r\right)I\left(t\right)+\varepsilon \left(r,t\right),$$

The index-driven trend in $X$ is then

6 $$\Delta {X}_{I}\left(r\right)=\,\beta \left(r\right)\Delta I,$$

where $\Delta I$ is the linear trend of $I\left(t\right)$. The index-driven contribution to the local trend is computed as

7 $${C}_{I}\left(r\right)=\frac{\Delta {X}_{I}\left(r\right)}{\Delta X\left(r\right)}$$

where $\Delta X$ is the observed or reconstructed trend of $X$ at location $r$.

For regional mean series ${X}_{R}\left(t\right)$, i.e., NE/WCE/MED mean PDSI and precipitation, we apply the same one-dimensional approach by regressing ${X}_{R}\left(t\right)$ onto $I\left(t\right)$. For the NS mode, we regress its principal component onto $I\left(t\right)$ and compute the index-driven trend contribution. We also consider an NS precipitation-gradient index, defined as the time expansion coefficient of the regression pattern obtained by regressing precipitation onto the NS-mode time series.

## Supplementary information

Supplementary Information

Transparent Peer Review file

## Data Availability

The EULMDA reconstruction generated in this study has been deposited at Zenodo (10.5281/zenodo.18254604). Source Data underlying main figures are provided with this paper. All tree-ring proxy records used in EULMDA are publicly available. CESM2-LE and CESM1-LME are available at the NCAR Climate Data Gateway (https://www.cesm.ucar.edu/community-projects/lens2; https://www.cesm.ucar.edu/community-projects/lme). CMIP6/PMIP4 data are available via the Earth System Grid Federation (https://esgf-node.ipsl.upmc.fr/search/cmip6-ipsl/). ERA5 reanalysis data are available from the Copernicus Climate Change Service Data Store (https://cds.climate.copernicus.eu/datasets). The 20CR reanalysis data can be accessed from NOAA; The GMF is available at https://hydrology.soton.ac.uk/data/pgf/v2/0.5deg/monthly/. The CRU instrumental PDSI dataset is available at https://crudata.uea.ac.uk/cru/data/drought/#global. The Old World Drought Atlas is available from the NOAA Paleoclimatology website at https://www.ncei.noaa.gov/access/paleo-search/study/19419; Great Eurasian Drought Atlas can be accessed at https://www.dropbox.com/scl/fi/ejnoni5tt3zitxl5qqu48/geda_trim.nc?rlkey=g1edzlkgxkoqoppt9wll080q7&e = 1&dl=0; The European summer temperature reconstruction by Luterbacher et al. (2016) is available at https://www.ncei.noaa.gov/access/paleo-search/study/19600; the PHYDA dataset is available at https://zenodo.org/records/1198817; ModE-RA is available at https://www.wdc-climate.de/ui/entry?acronym=ModE-RA; The instrumental Hurrell PC-based NAO index is available at https://climatedataguide.ucar.edu/climate-data/hurrell-north-atlantic-oscillation-nao-index-pc-based. The instrument-based MO index is available at https://crudata.uea.ac.uk/cru/data/moi/.

## Code availability

The offline DA code utilizing a particle filter is available at Zenodo^103^ and GitHub (https://github.com/dalaiden/DA_offline_PF). The code used to generate the main figures, together with the underlying plotting data (Source Data), is available via Figshare at 10.6084/m9.figshare.31252093.
