# Supplementary material for: Scandinavian pattern and temperature changes shape European summer droughts over the past millennium
Source: Nat Commun. 2026 Apr 27;17:7332. doi: 10.1038/s41467-026-72385-w (PMC13402590; doi:10.1038/s41467-026-72385-w)
Supplement: Supplementary file 1 — Supplementary Information [file 41467_2026_72385_MOESM1_ESM.pdf]

Supplementary Materials

Supplementary Materials for

Scandinavian pattern and temperature changes shape European summer droughts over the past millennium

Huihong Xue[1], Hugues Goosse[1], Quentin Dalaiden[2,3], Kristina Seftigen[4], Fabio Gennaretti[5,6], Feng Shi[7]

1 Earth and Life Institute, Université catholique de Louvain, Louvain-la-Neuve, Belgium
2 Bjerknes Center for Climate Research, Bergen, Norway
3 Nansen Environmental and Remote Sensing Center, Bergen, Norway
4 Gothenburg University Laboratory for Dendrochronology, Department of Earth Sciences, University of Gothenburg, Gothenburg, Sweden
5 Department of Agricultural, Food and Environmental Sciences, Università Politecnica delle Marche, Ancona, Italy
6 Institut de Recherche sur les Forêts, Groupe de Recherche en Écologie de la MRC-Abitibi, Université du Québec en Abitibi-Témiscamingue, Amos, Canada
7 State Key Laboratory of Lithospheric and Environmental Coevolution, Institute of Geology and Geophysics, Chinese Academy of Sciences, Beijing, China

Corresponding author: Huihong Xue (huihong.xue@uclouvain.be)

Contents

- **Supplementary Text**
- **Supplementary References**
- **Supplementary Figures 1–23**
- **Supplementary Tables 1–3**

Supplementary Text | Extended validation of EULMDA climate reconstruction

- **Validation based on instrumental observations over the last decades.**

EULMDA is first validated against instrumental and reanalysis products. EULMDA exhibits significant positive correlations with ERA5 for JJA (June–August) near-surface air temperature, precipitation, sea level pressure, and 800-hPa geopotential height across most of Europe and the Mediterranean during 1940–2000 CE (Fig. 1a–d). Over European land grid cells (10°W–30°E, 40°N–73°N), 91% and 37% of grid cells exhibit correlations above 0.5 for temperature and precipitation, respectively. The mean squared error skill score (MSESS) broadly mirrors the correlation patterns: it is positive over most of Europe, while negative values occur mainly over North Africa for precipitation (Supplementary Fig. 2).

At the regional scale, EULMDA also reproduces observed interannual variability. Regional-mean JJA temperature and precipitation series for northern Europe (NE), western-central Europe (WCE) and the Mediterranean (MED) correlate well with instrument-based products (Supplementary Fig. 22). Using GMF over 1901–2000 CE, correlations for temperature are r = 0.84* (NE), 0.75* (WCE) and 0.71* (MED); and for precipitation r = 0.65* (NE), 0.69* (WCE), and 0.66* (MED). Using ERA5 over 1950–2000 CE, correlations for temperature are r = 0.82* (NE), 0.69* (WCE), and 0.79* (MED), and for precipitation are r = 0.65* (NE), 0.66* (WCE), and 0.65* (MED).

We further assess the EULMDA-derived JJA Palmer Drought Severity Index (PDSI) against two instrument-based datasets: CRU[1] and Dai PDSI[2], both based on Penman-Monteith equation for net evapotranspiration. EULMDA agrees well with both products. For CRU, 58% of land grid cells exhibit correlations above 0.5 (Fig. 1e), and regional correlations over 1901–2000 CE are 0.62* for NE, 0.70* for WCE, and 0.75* for the MED (Supplementary Fig. 22). Comparisons with Dai PDSI show even stronger agreement, with 70% of grid cells exceeding r = 0.5 (Fig. 1f), and regional correlations of 0.75* (NE), 0.69* (WCE), and 0.62* (MED) over 1950–2000 CE. The ranges of correlation and MSESS across European land grid cells for all reconstructed variables are summarized in Supplementary Tab. 3.

In addition to correlation and skill scores, we quantify how well EULMDA reproduces interannual variance during the instrumental period by computing the ratio of the interannual standard deviation in EULMDA to that in instrument-based products at each grid point, as well as for the NE, WCE and MED regional means. For JJA temperature, 81% of land grid cells show

standard-deviation ratios between 0.5 and 1. For PDSI, 47% of land grid cells fall between 0.5 and 1 relative to CRU and 27% relative to the Dai PDSI, indicating moderate but not dramatic variance damping (Supplementary Fig. 23). In contrast, precipitation shows stronger variance reduction, with most land grid cells showing ratios below 0.5. The regional mean series similarly show that EULMDA captures year-to-year variations reasonably well (high correlations with instrumental products), with somewhat reduced amplitude for temperature and PDSI, and more pronounced damping variability for precipitation (Supplementary Fig. 22).

- **Influence of proxy availability on reconstruction skill**

To evaluate the influence of proxy availability, we perform two additional experiments using subsets of 30 and 53 tree-ring records that span the periods CE 1306 and 1600 to the present, respectively (Supplementary Fig. 1d–e). Both experiments yield statistically significant correlations with instrumental products for temperature, precipitation, pressure, and PDSI during 1901–2000 CE (Supplementary Fig. 9). This suggests that the phase of the reconstructed variability remains largely robust to differences in tree-ring coverage. In contrast, the amplitude, as reflected in the variance, is more sensitive, with sparser networks yielding dampened signals (Supplementary Fig. 10). This amplitude reduction before ~1600 CE partly reflects limited proxy constraints during earlier centuries; in the absence of assimilated data at a given location and time, the posterior is equivalent to the prior (see Methods), yielding near-zero variance.

- **Comparison with existing reconstructions during 1000–2000 CE**

EULMDA aligns closely with the summer temperature reconstruction of Luterbacher, et al. (2016)[3], which is based on tree-ring and documentary historical records, with a correlation of 0.80* over 1000–2000 CE (Supplementary Fig. 7a). However, we should note the partial overlap in the underlying proxy networks. Comparisons with both the Old World Drought Atlas (OWDA)[4] and the Great Eurasian Drought Atlas (GEDA)[5] reveal substantial spatial agreement in hydroclimatic variations (Supplementary Fig. 7b–c). OWDA has been widely used and analyzed[4,6], while GEDA is a newer tree-ring based reconstruction of PDSI, including more data from the East European Plain to the Ural Mountains[5]. Over the past millennium, regional-mean PDSI correlations between EULMDA and GEDA/OWDA reach 0.41*/0.49* in NE, 0.48*/0.42* in WCE, and 0.56*/0.53* in MED (Fig. 1g–i).

In addition, EULMDA is compared with two DA-based reconstructions: the Modern Era Reanalysis (ModE-RA) and the Paleo Hydrodynamics Data Assimilation product (PHYDA).

ModE-RA, spanning 1421–2008 CE, is based on ECHAM6 simulations and utilizing natural proxies and documentary data in earlier periods, while incorporating instrumental measurements from the 17th century onward[7]. PHYDA, covering 1000–2000 CE, is based on multiple paleo-proxy records and the CESM-LME[8]. Over their common periods, both products show extensive spatial agreement with EULMDA for JJA temperature, precipitation and pressure over the most of Europe (Supplementary Fig. 7d–g). For PDSI, EULMDA and PHYDA exhibit broadly comparable skill with respect to CRU over 1901–2000 CE, with EULMDA performing slightly better in parts of the Mediterranean and high-latitude northern Europe (Supplementary Fig. 7h–i). In contrast, EULMDA precipitation generally shows weaker agreement with instrumental products than ModE-RA (ModE-RA doesn't provide PDSI; Supplementary Fig. 7j), which is expected because ModE-RA assimilates instrumental station data during the 19th–20th centuries, whereas EULMDA is constrained only by tree-ring. For JJA regional means, correlations with ModE-RA over 1421–2000 CE are 0.81* (NE), 0.67* (WCE) and 0.64* (MED) for temperature; and 0.46* (NE), 0.61* (WCE) and 0.44* (MED) for precipitation. Correlations with PHYDA over 1000–2000 CE are 0.85* (NE), 0.74* (WCE) and 0.68* (MED) for temperature, and 0.54* (NE), 0.46* (WCE), 0.54* (MED) for PDSI. All correlations are computed using JJA regional-mean anomalies.

Note: All correlation coefficients marked with an asterisk are significant at $p < 0.05$. The definitions of the three subregions: Northern Europe (NE), Western–Central Europe (WCE), and the Mediterranean (MED) are provided in Supplementary Fig. 1a.

References

1. van der Schrier, G., Barichivich, J., Briffa, K. & Jones, P. A scPDSI-based global data set of dry and wet spells for 1901–2009. *J. Geophys. Res. Atmos.* **118**, 4025–4048 (2013).
2. Dai, A., Trenberth, K. E. & Qian, T. A global dataset of Palmer Drought Severity Index for 1870–2002: Relationship with soil moisture and effects of surface warming. *J. Hydrometeorol.* **5**, 1117–1130 (2004).
3. Luterbacher, J. et al. European summer temperatures since Roman times. *Environ. Res. Lett.* **11**, 024001 (2016).
4. Cook, E. R. et al. Old World megadroughts and pluvials during the Common Era. *Sci. Adv.* **1**, e1500561 (2015).
5. Cook, B. I., Cook, E. R., Anchukaitis, K. J. & Singh, D. Characterizing the 2010 Russian heatwave-Pakistan flood concurrent extreme over the last millennium using the Great Eurasian Drought Atlas. *J. Clim.* **37**, 4389–4401 (2024).

6. Cook, B. I. et al. Megadroughts in the Common Era and the Anthropocene. *Nat. Rev. Earth Environ.* **3**, 741–757 (2022).
7. Valler, V. et al. ModE-RA: a global monthly paleo-reanalysis of the modern era 1421 to 2008. *Sci. Data* **11**, 36 (2024).
8. Steiger, N. J., Smerdon, J. E., Cook, E. R. & Cook, B. I. A reconstruction of global hydroclimate and dynamical variables over the Common Era. *Sci. Data* **5**, 1–15 (2018).
9. Iturbide, M. et al. An update of IPCC climate reference regions for subcontinental analysis of climate model data: definition and aggregated datasets. *Earth Syst. Sci. Data* **12**, 2959–2970 (2020).

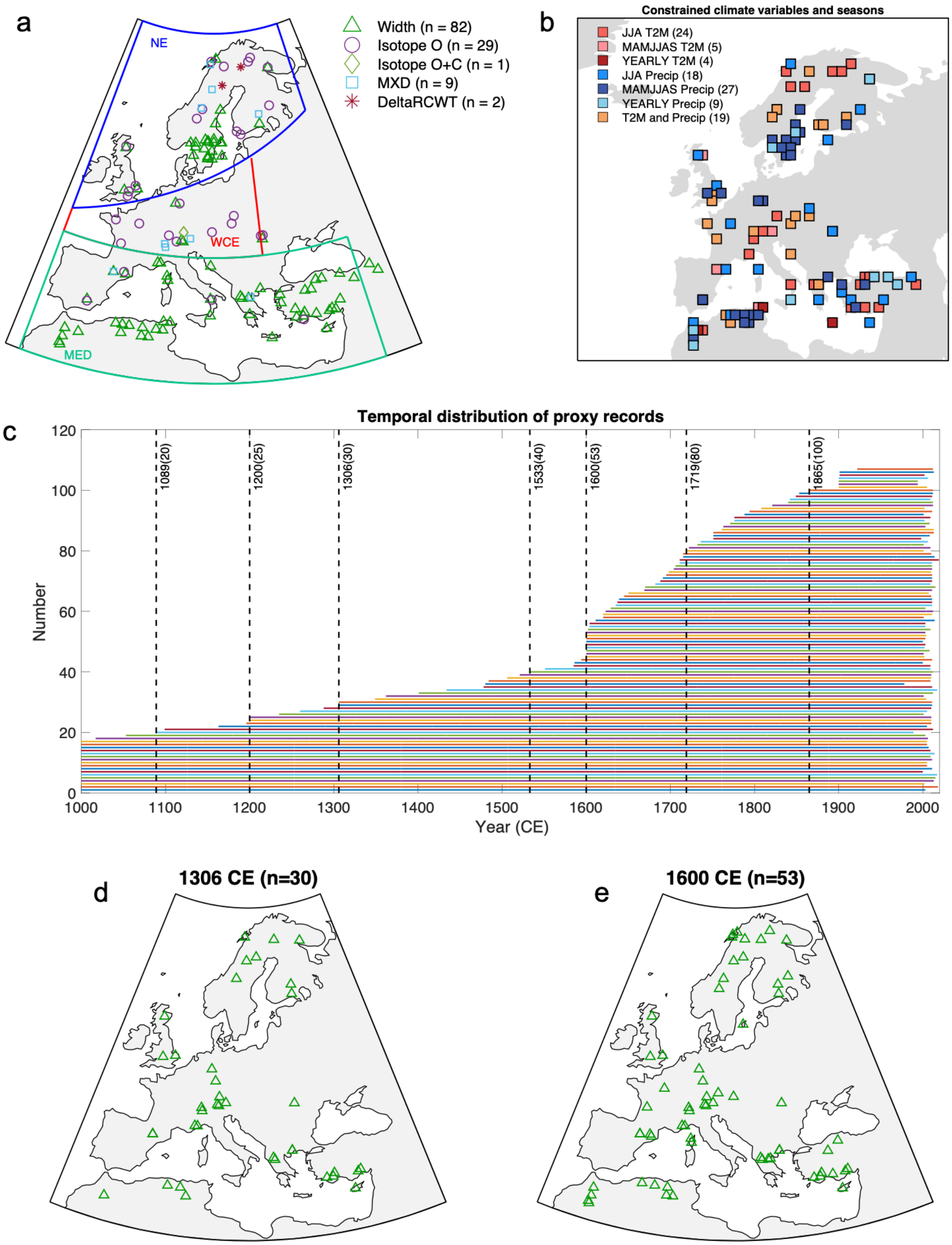

Supplementary Fig. 1 | Spatial-temproal distribution and climate interpretation of the tree-ring network. (**a**) Spatial distribution of tree-ring proxy records across northern Europe (NE),

western-central Europe (WCE), and the Mediterranean (MED), with symbols indicating different types of proxies: tree-ring width, oxygen isotopes (isotope O), carbon and oxygen isotopes (isotope O+C), maximum latewood density (MXD), and the delta radial cell wall thickness (DeltaRCWT). Regional domains follow the IPCC reference regions (ref. [9]), except that the eastern boundary of WCE is set to 25°E (shifted westward relative to the IPCC definition) due to limited record availability in the far-eastern part of the IPCC WCE region. (**b**) The climate interpretation of the tree-ring records. The squares represent tree-ring records, with colors indicating the climate variables represented by the records, as determined by BIC values of PSMs. 'T2M' represents near-surface temperature, and 'Precip' represents precipitation. 'JJA' refers to the June-August summer period, while 'MAMJJAS' refers to the growing season from March to September. The number of records corresponding to each climate variable is noted in parentheses. (**c**) Temporal distribution of tree-ring records from 1000 CE to 2000 CE. Vertical dashed lines are added at selected time points to help illustrate the evolution of record availability through time. (**d–e**) Spatial distribution of tree-ring records for 1306-2000 CE (n=30; d) and 1600-2000 CE (n=53; e). Two experiments (Exp-1306 CE and Exp-1600 CE) are performed using these subsets of tree-ring records, respectively.

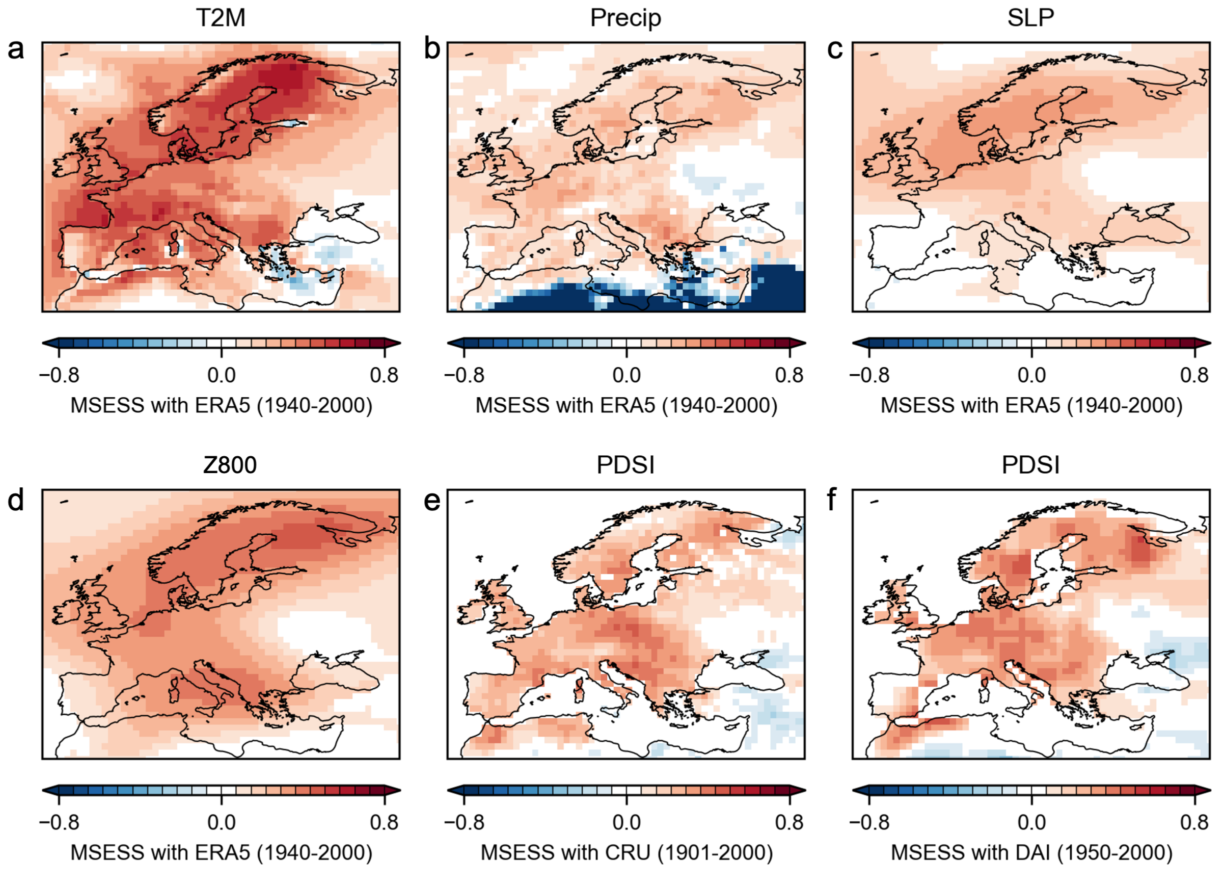

Supplementary Fig. 2 | Mean squared error skill score (MSESS) of EULMDA against instrumental products. Spatial distribution of MSESS for JJA near-surface temperature (T2M; **a**), precipitation (Precip; **b**), sea level pressure (SLP; **c**), and 800-hPa geopotential height (Z800; d) evaluated against ERA5 over 1940–2000 CE. (**e–f**) MSESS for JJA PDSI evaluated against CRU PDSI over 1901–2000 CE (e) and Dai PDSI over 1950–2000 CE (f). Positive MSESS values indicate improved performance (lower mean squared error) compared to the climatology of the evaluated period.

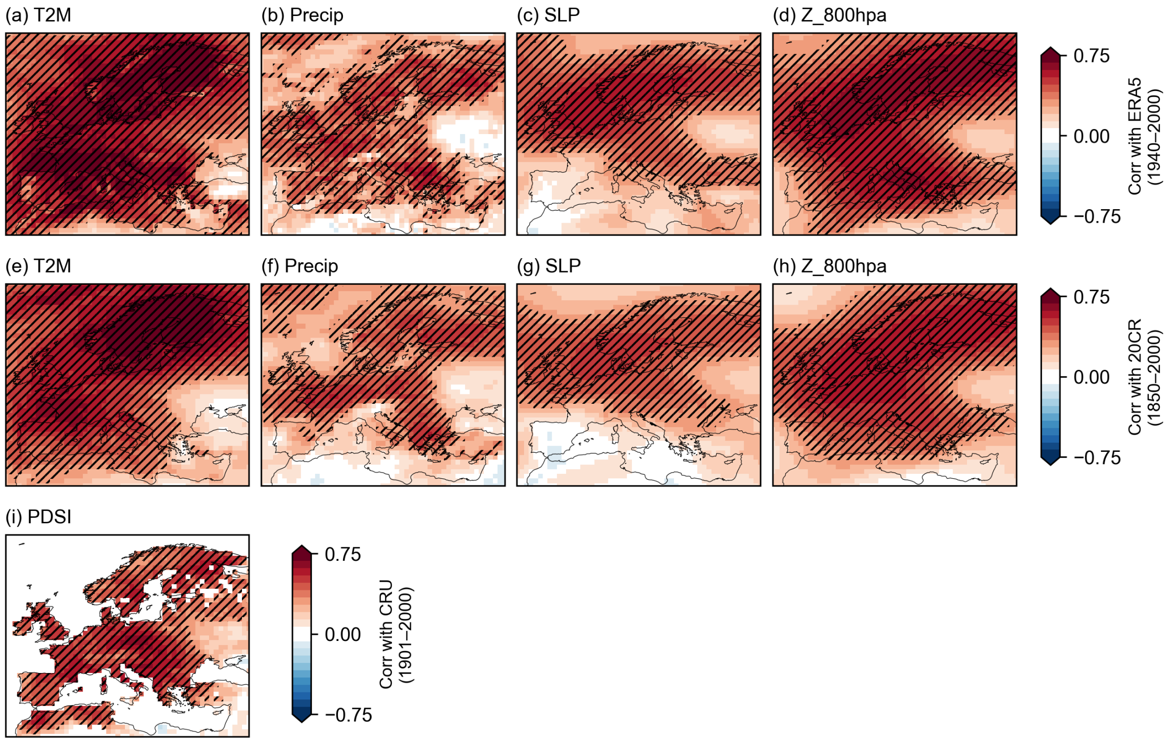

Supplementary Fig. 3 | Median of correlation coefficient for the five reconstruction members based on different model priors. (**a–d**) correlation of JJA near-surface air temperature (T2M; a), precipitation (Precip; b), sea level pressure (SLP; c), and 800-hPa geopotential height (Z800; d) with ERA5 during the period 1940–2000 CE. (**e–h**) correlation of these variables with 20CR over the period 1850–2000 CE. (**i**) correlation of JJA PDSI with CRU during 1901–2000 CE. Stippling indicates statistically significant correlations at the 95% confidence level. If more than three reconstruction members show significance at this level, the correlation is considered significant.

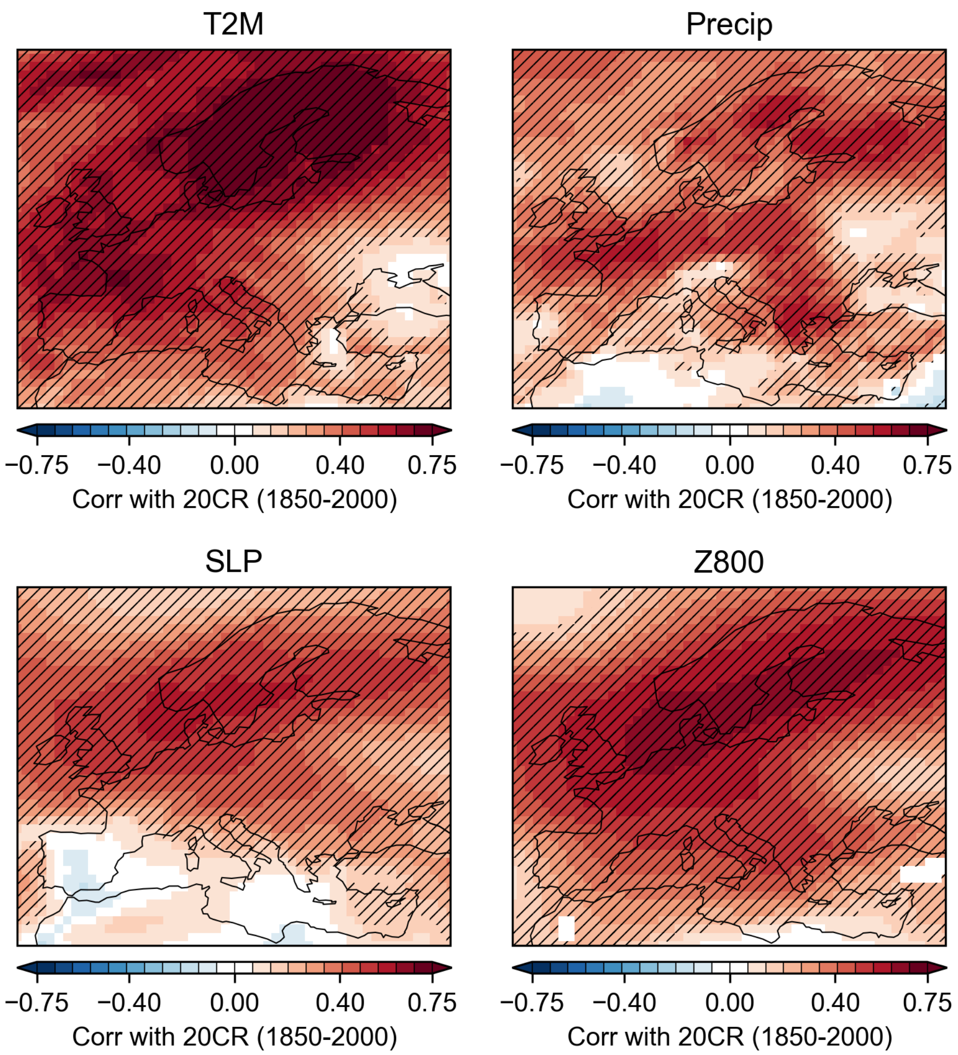

Supplementary Fig. 4 | Validation based on 20CR reanalysis over 1850–2000 CE. Correlations of JJA near-surface temperature (T2M), precipitation (Precip), sea-level pressure (SLP), and 800-hPa geopotential height (Z800) in EULMDA with 20CR reanalysis over 1850–2000 CE. Hatching indicates regions with statistically significant correlations ($p < 0.05$).

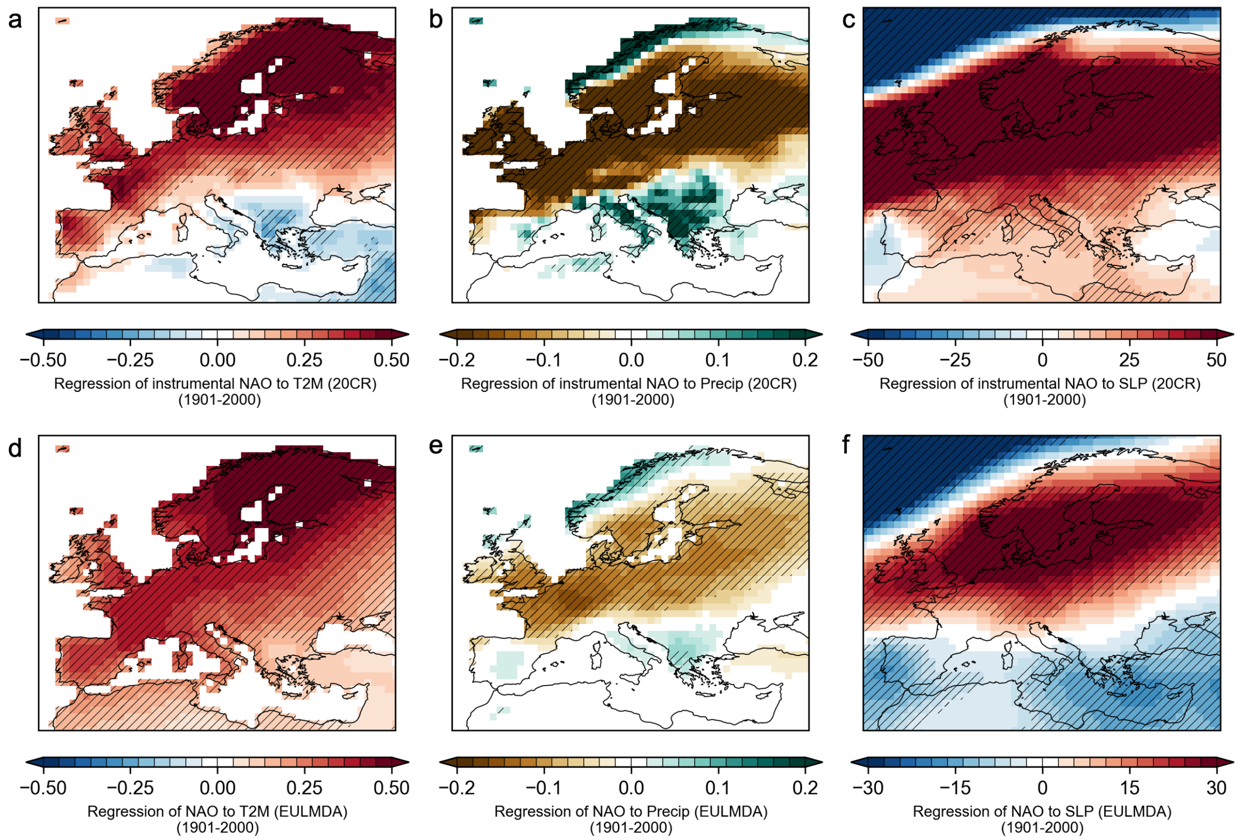

Supplementary Fig. 5 | Comparisons of climate fields associated with JJA NAO between instrument-based products and EULMDA. (**a–c**) Regression between Hurrell NAO Index (PC-based) and 20CR reanalysis data for near-surface temperature (T2M; K; a), precipitation (Precip; mm d^{-1}; b), and sea-level pressure (SLP; Pa; c) anomalies during 1901–2000 CE. (**d–f**) Regression between NAO and T2M (d), Precip (e), and SLP (f) anomalies in EULMDA during 1901–2000 CE. Hatched areas indicate statistical significance ($p < 0.05$). The consistent spatial patterns between instrumental and reconstructed regressions support the validity of EULMDA in capturing NAO-related climate variability.

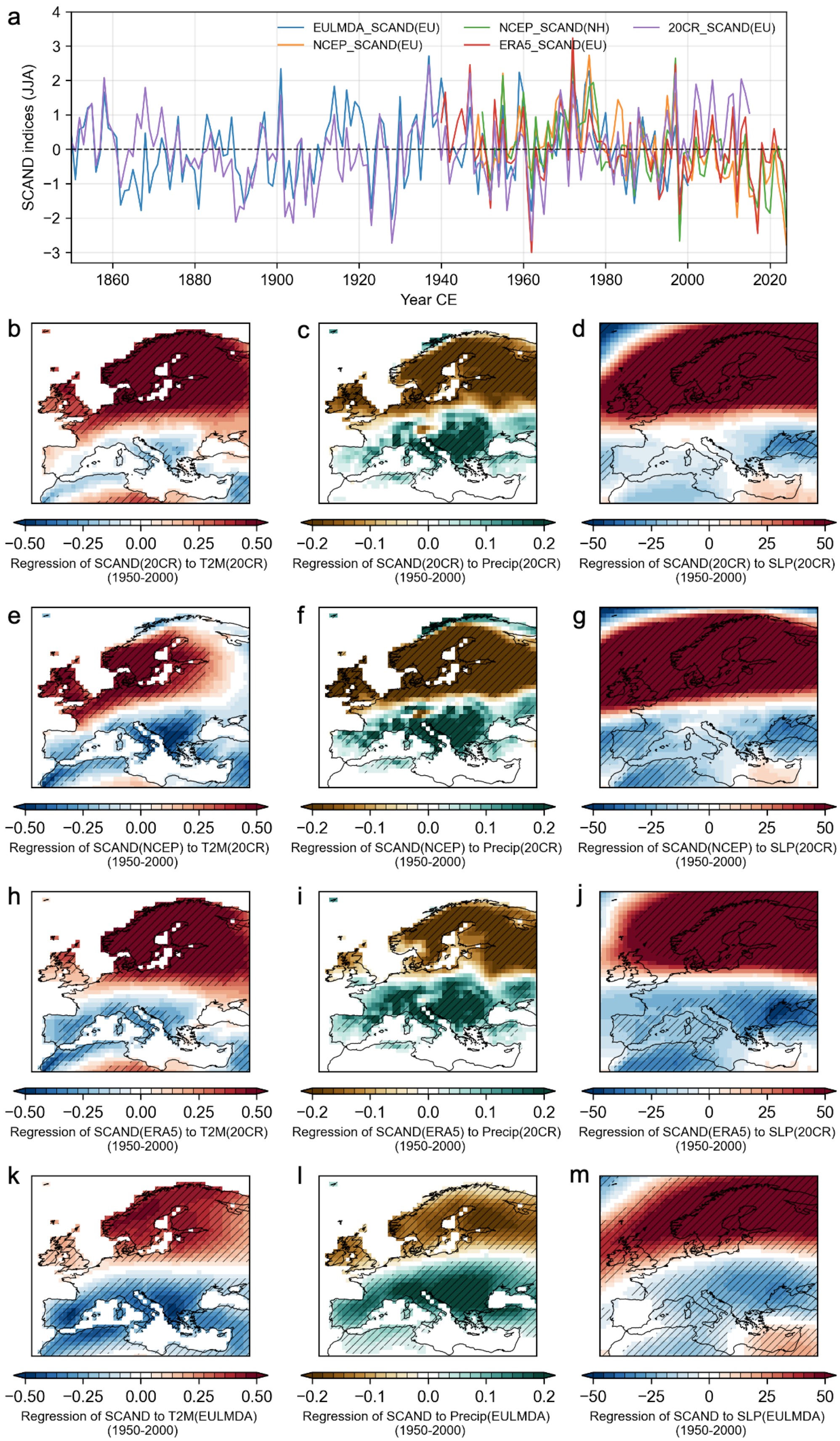

a
SCAND indices (JJA)
EULMDA_SCAND(EU)
NCEP_SCAND(EU)
NCEP_SCAND(NH)
ERA5_SCAND(EU)
20CR_SCAND(EU)
Year CE
b
c
d
e
f
g
h
i
j
k
l
m
Regression of SCAND(20CR) to T2M(20CR) (1950-2000)
Regression of SCAND(20CR) to Precip(20CR) (1950-2000)
Regression of SCAND(20CR) to SLP(20CR) (1950-2000)
Regression of SCAND(NCEP) to T2M(20CR) (1950-2000)
Regression of SCAND(NCEP) to Precip(20CR) (1950-2000)
Regression of SCAND(NCEP) to SLP(20CR) (1950-2000)
Regression of SCAND(ERA5) to T2M(20CR) (1950-2000)
Regression of SCAND(ERA5) to Precip(20CR) (1950-2000)
Regression of SCAND(ERA5) to SLP(20CR) (1950-2000)
Regression of SCAND to T2M(EULMDA) (1950-2000)
Regression of SCAND to Precip(EULMDA) (1950-2000)
Regression of SCAND to SLP(EULMDA) (1950-2000)

Supplementary Fig. 6 | Comparison of JJA SCAND variability and its climate signature in instrument-based products and EULMDA. (a) JJA SCAND index defined over the European sector (EU; 20°–80°N, 40°W–12°E) from EULMDA (blue), ERA5 (red), NCAR/NCEP (orange) and 20CR (purple), together with the canonical hemispheric SCAND index (NH; green) downloaded from https://psl.noaa.gov/data/timeseries/month/SCAND/. All indices are standardized to unit variance. The EU-sector and canonical definitions are highly consistent: r = 0.74* between NCEP_SCAND(EU) and NCEP_SCAND(NH) over 1950–2024 CE, and r = 0.61* between EULMDA_SCAND(EU) and NCEP_SCAND(NH) over 1950–2000 CE (asterisk denotes $p < 0.05$). **(b–d)** Regression of 20CR JJA anomalies in near-surface temperature (T2M; K; b), precipitation rate (Precip; mm d^{-1}; c) and sea-level pressure (SLP; Pa; d) against the 20CR-derived SCAND(EU) index during 1950–2000 CE. **(e–g)** As in (b–d), but regressing 20CR fields against the NCEP/NCAR-derived SCAND(EU) index. **(h–j)** As in (b–d), but regressing 20CR fields against the ERA5-derived SCAND(EU) index. **(k–m)** As in (b–d), but regressing EULMDA fields against the EULMDA-derived SCAND(EU) index. Hatched areas indicate statistical significance ($p < 0.05$). The consistent spatial patterns between instrument-based and reconstructed regressions support the validity of EULMDA in capturing SCAND-related climate variability.

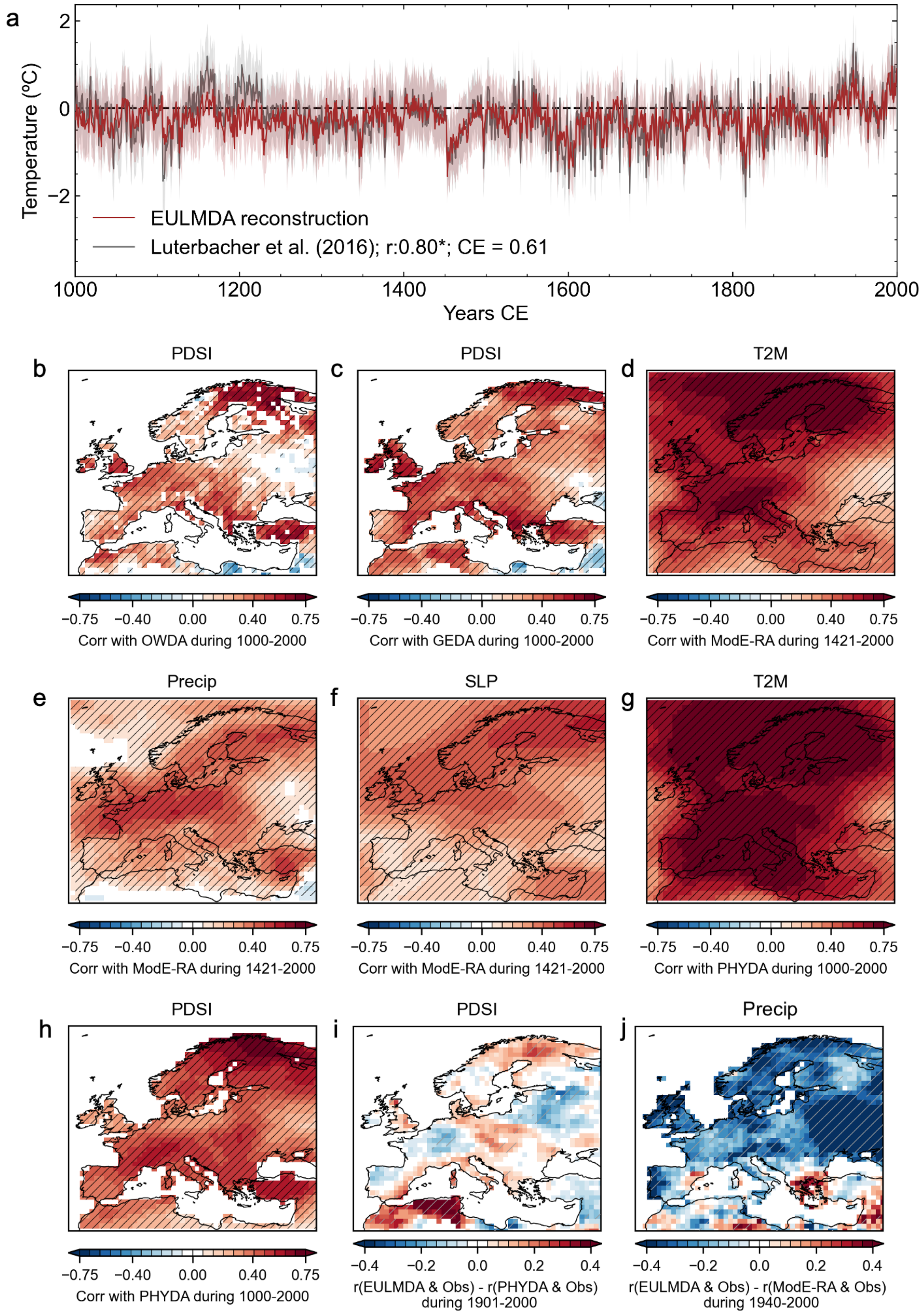

a
Temperature (°C)
2
0
−2
EULMDA reconstruction
Luterbacher et al. (2016); r:0.80*; CE = 0.61
1000
1200
1400
1600
1800
2000
Years CE
b
PDSI
Corr with OWDA during 1000-2000
c
PDSI
Corr with GEDA during 1000-2000
d
T2M
Corr with ModE-RA during 1421-2000
e
Precip
Corr with ModE-RA during 1421-2000
f
SLP
Corr with ModE-RA during 1421-2000
g
T2M
Corr with PHYDA during 1000-2000
h
PDSI
Corr with PHYDA during 1000-2000
−0.75 −0.40 0.00 0.40 0.75
i
PDSI
r(EULMDA & Obs) - r(PHYDA & Obs) during 1901-2000
j
Precip
r(EULMDA & Obs) - r(ModE-RA & Obs) during 1940-2000
−0.4 −0.2 0.0 0.2 0.4

Supplementary Fig. 7 | Comparison of EULMDA with existing reconstructions. (**a**) JJA European mean surface temperature from EULMDA (ensemble mean with spread) compared with Luterbacher et al. (2016), which is based on tree-ring and documentary historical records, over 1000–2000 CE. (**b–c**) Correlations of JJA PDSI from EULMDA with tree-ring based reconstructions of OWDA and GEDA for 1000–2000 CE. (**d–f**) Correlations of JJA temperature (T2M; d), precipitation (Precip; e) and sea level pressure (SLP; f) from EULMDA with ModE-RA for 1421–2000 CE. (**g–h**) Correlations of T2M and PDSI between EULMDA and PHYDA for 1000–2000 CE. (**i**) Difference in correlation with CRU PDSI between EULMDA and PHYDA over 1901–2000 CE, computed as *r*(EULMDA, CRU) – *r*(PHYDA, CRU). **(j)** Difference in correlation with ERA5 precipitation between EULMDA and ModE-RA over 1940–2000 CE, computed as *r*(EULMDA, ERA5) – *r*(ModE-RA, ERA5). Hatched areas indicate statistically significant correlations ($p < 0.05$).

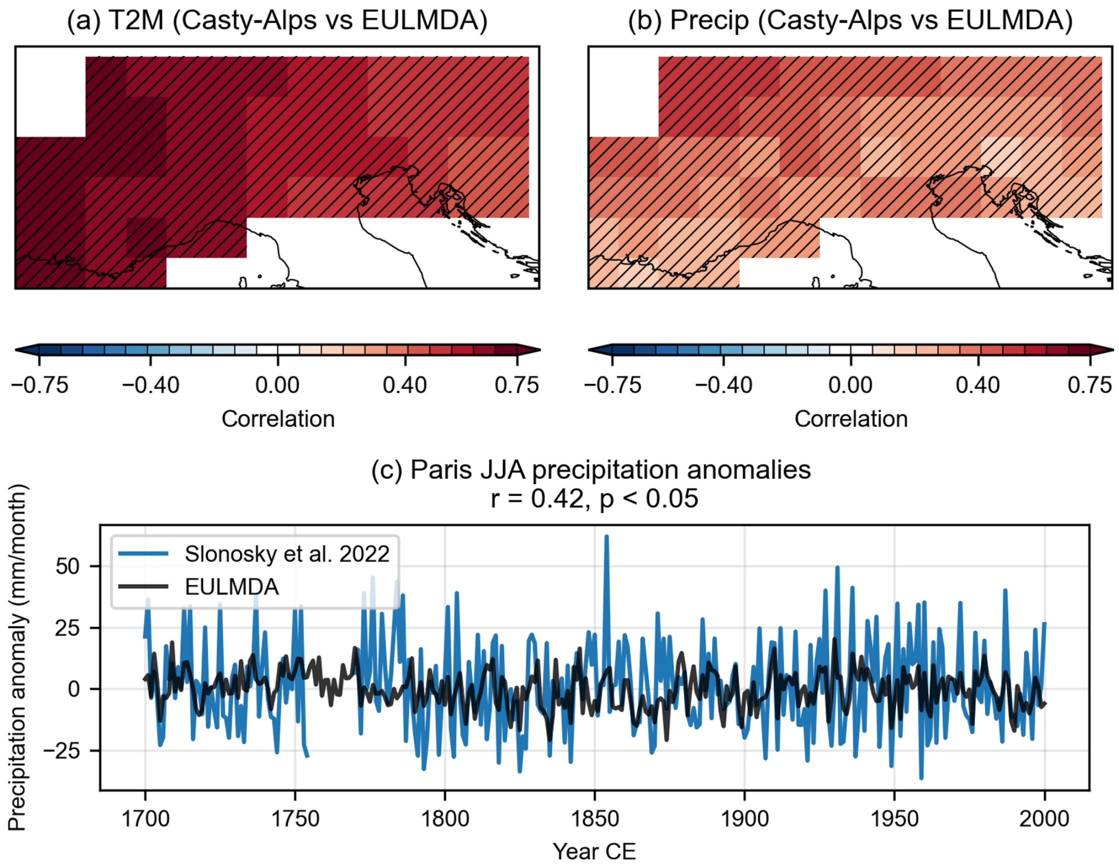

Supplementary Fig. 8 | Independent validation of EULMDA using early instrumental–documentary and station-based records. (**a**–**b**) Correlations between EULMDA and the Casty et al. (2005) Alpine reconstruction for JJA near-surface air temperature (T2M; a) and precipitation (Precip; b) over 1700–2000 CE. (**c**) Comparison of JJA precipitation anomalies (mm month^{-1}) from the Paris station record (Slonosky, 2002; blue) and the nearest EULMDA grid cell (black) over 1700–2000 CE. Hatched areas in (a–b) indicate correlations significant at $p < 0.05$.

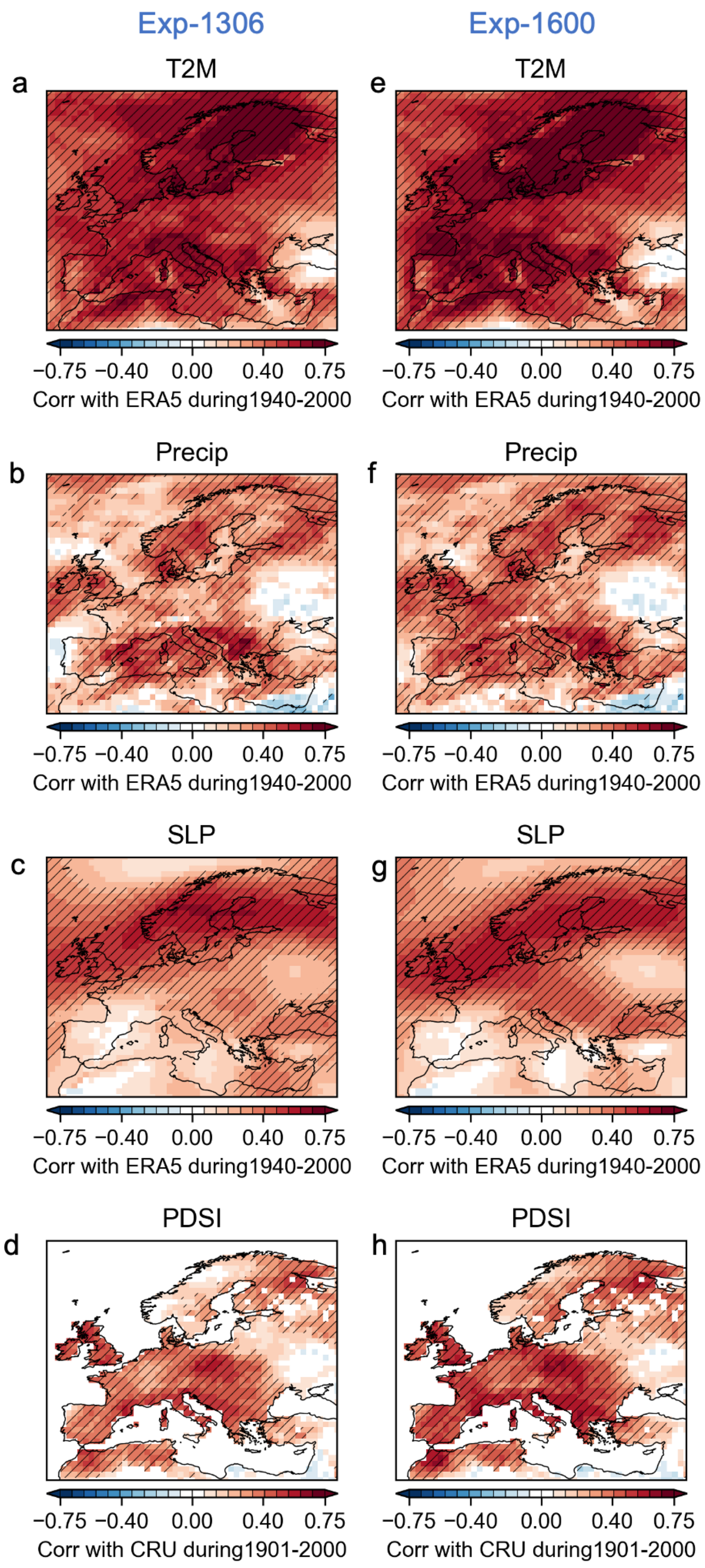

Supplementary Fig. 9 | Comparison of Exp-1306 and Exp-1600 reconstructions with instrument-based datasets. Exp-1306 (**a–d**) and Exp-1600 (**e–h**) were reconstructed using 30

and 53 tree-ring records, respectively, covering the periods 1306–2000 CE and 1600–2000 CE (the spatial distributions of records are shown in Supplementary Fig. 1d–e). Both experiments employed CESM1-LME as the prior ensemble. (a–c) and (e–g) show correlation maps between reconstructed and ERA5-based fields for JJA near surface temperature (T2M; a, e), sea-level pressure (SLP; b, f), and precipitation (Precip; c, g) over 1940–2000 CE. (d) and (h) show correlations between reconstructed PDSI and CRU observations over 1901–2000 CE. Hatching indicates statistically significant correlations ($p < 0.05$). Both experiments yield statistically significant correlations with instrumental data across most of Europe, suggesting that the phase of the reconstructed variability remains largely robust to differences in tree-ring coverage.

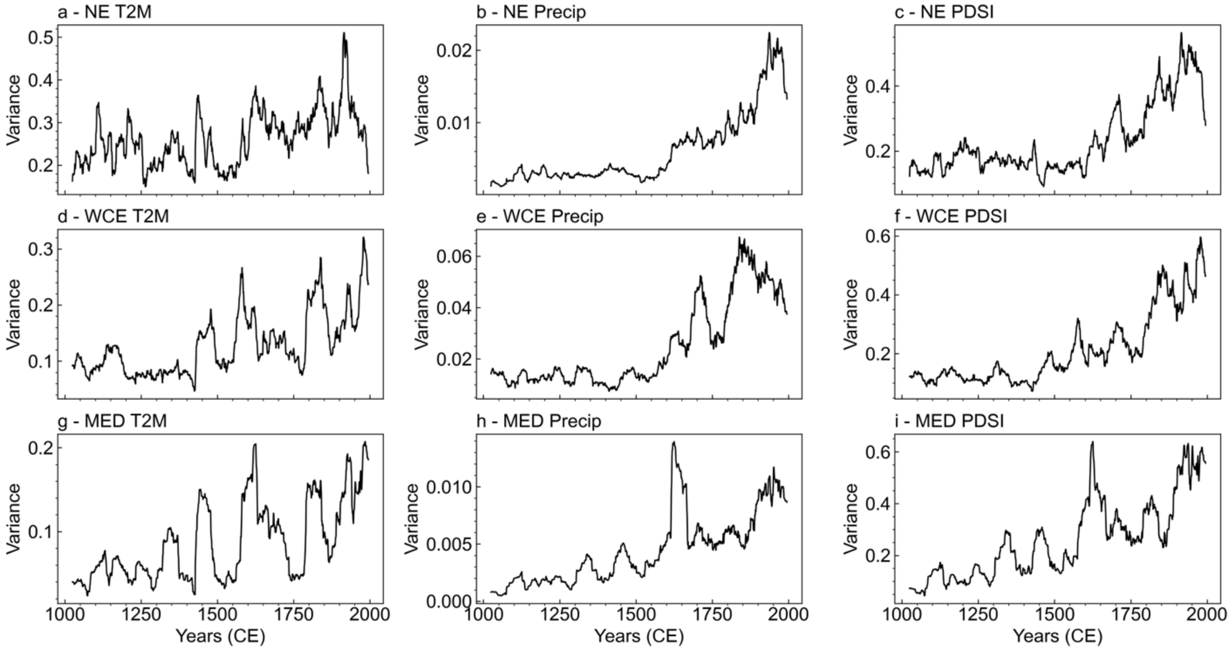

Supplementary Fig. 10 | Amplitude changes in regional mean climate variability over the past millennium. The amplitude is reflected using variance. 51-year running variance of JJA temperature (T2M; K), precipitation (Precip; mm d^{-1}), and PDSI anomalies for northern Europe (NE; a–c), west-central Europe (WCE; d–f), and the Mediterranean (MED; g–i). This amplitude reduction before ~1600 CE partly reflects limited proxy constraints during earlier centuries; in the absence of assimilated data at a given location and time, the posterior is equivalent to the prior, yielding near-zero variance of the mean reconstruction.

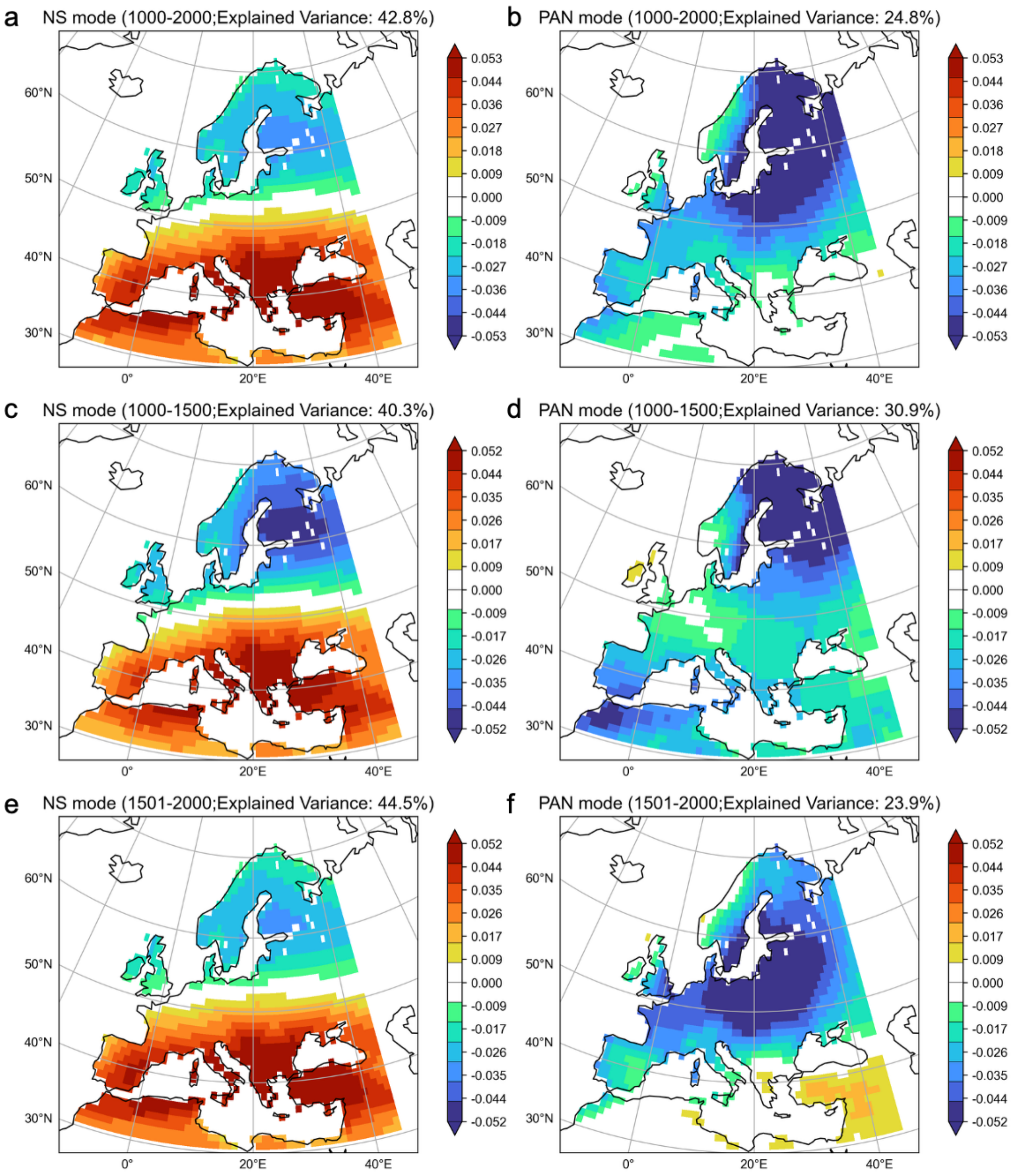

Supplementary Fig. 11 | Robustness of the leading European summer drought modes across subperiods. Spatial loadings of the EOF-derived drought modes computed from JJA PDSI for the full period 1000–2000 CE (NS mode; a, and PAN mode; b), and for two subperiods 1000–1500 CE (NS mode; c, and PAN mode; d) and 1501–2000 CE (NS mode; e, and PAN mode; f).

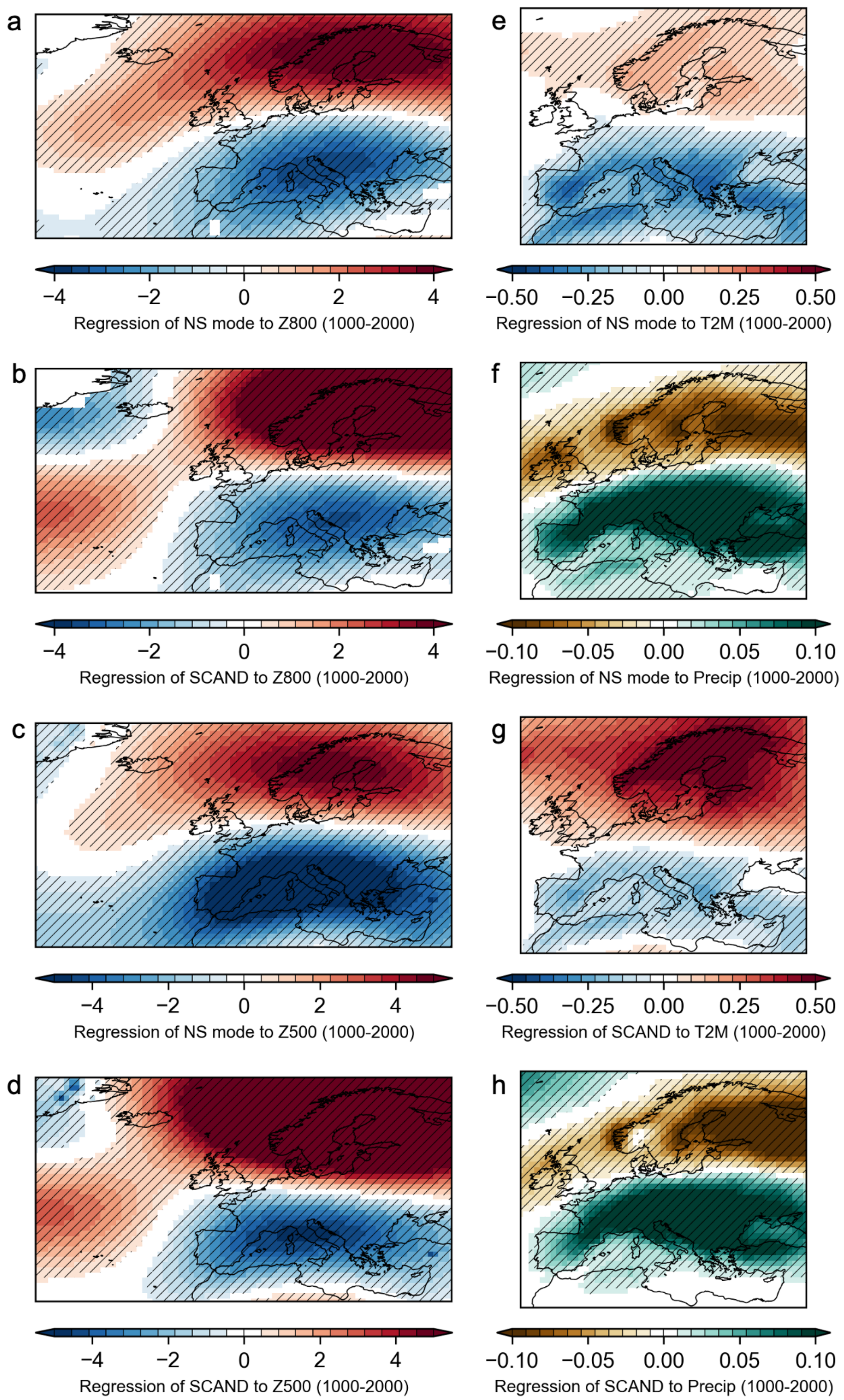

Supplementary Fig. 12 | Vertical structure of the NS/SCAND circulation signal and associated surface climate anomalies. Regression of JJA geopotential height anomalies (m) at

800 hPa (Z800; a–b) and 500 hPa (Z500; c–d), and of near-surface temperature (T2M; K; e, g) and precipitation (Precip; mm d^{-1}; f, h) anomalies, against the NS-mode index (a, c, e, f) and the European-sector SCAND index (b, d, g, h) over 1000–2000 CE. Hatching indicates statistically significant regression coefficients ($p < 0.05$).

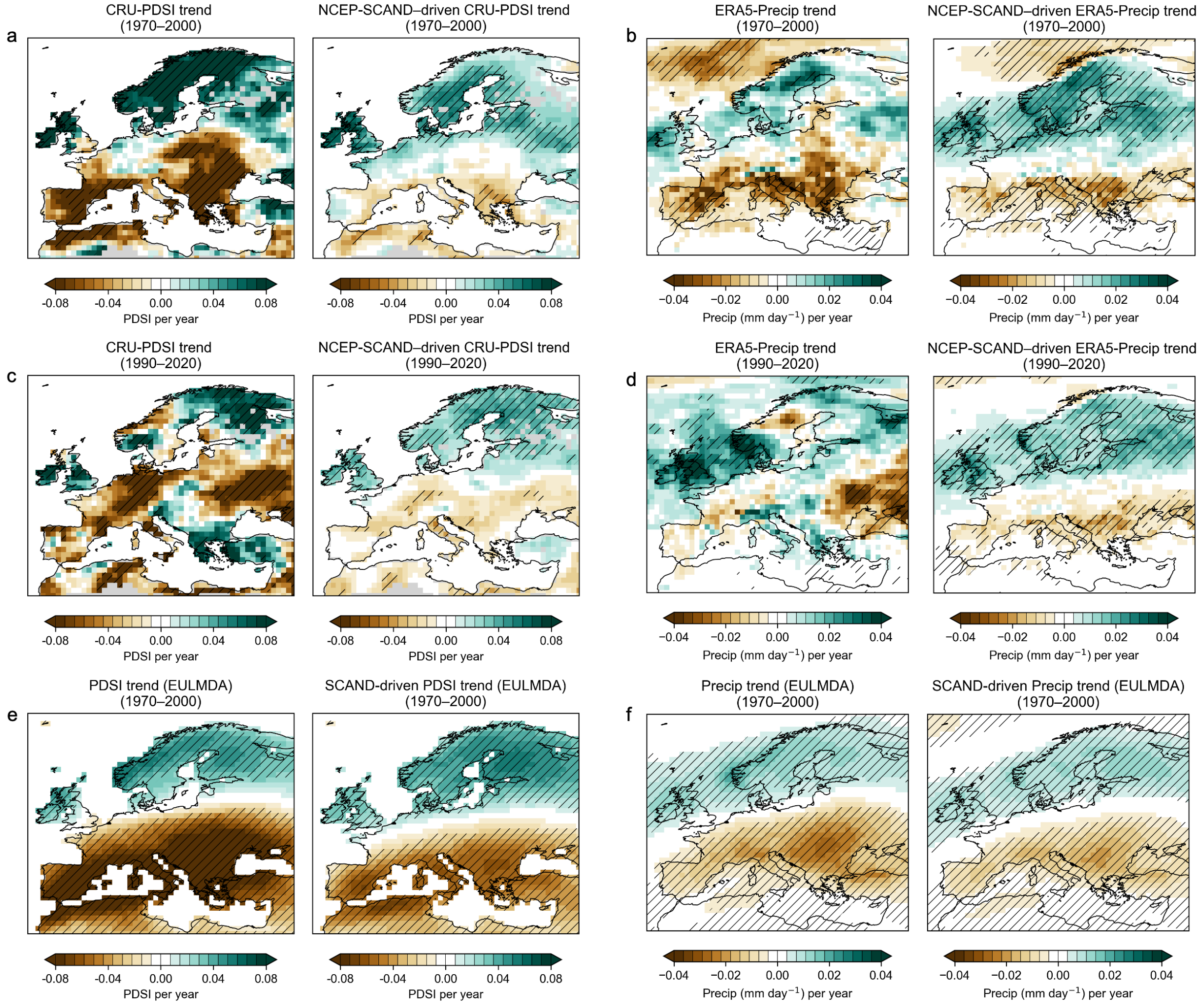

Supplementary Fig. 13 | Observed and reconstructed summer hydroclimate trends and their SCAND-related contribution. Spatial trends in JJA PDSI (left two columns) and precipitation (Precip; right two columns) from instrument-based reanalysis and EULMDA, together with the corresponding trend contributions from SCAND index. (**a,c,e**) JJA PDSI trends from CRU PDSI over 1970–2000 CE (a), and 1990–2020 CE (c), and from EULMDA over 1970–2000 CE (e). (**b,d,f**) JJA Precip (mm day^{-1}) trends from ERA5 over 1970–2000 CE (b),

and 1990–2020 CE (d), and from EULMDA over 1970–2000 CE (f). For each dataset and period, the adjacent panel shows the SCAND-driven contribution. Hatching indicates areas where trends are statistically significant ($p < 0.05$).

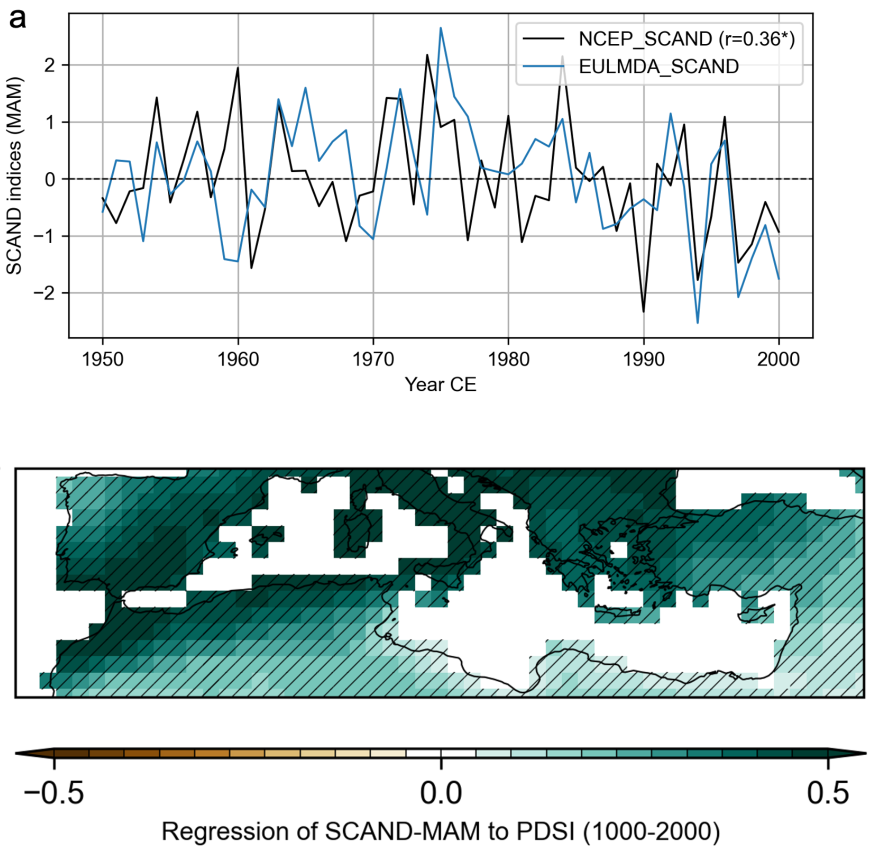

Supplementary Fig. 14 | Link between spring SCAND variability and subsequent summer drought in EULMDA. (**a**) Spring (MAM) SCAND indices from EULMDA and NCEP/NCAR over the overlapping period (shown for reference). (**b**) Regression of JJA PDSI onto the MAM SCAND index in EULMDA over 1000–2000 CE. Hatching indicates regions where the regression is statistically significant at the 95% confidence level ($p < 0.05$).

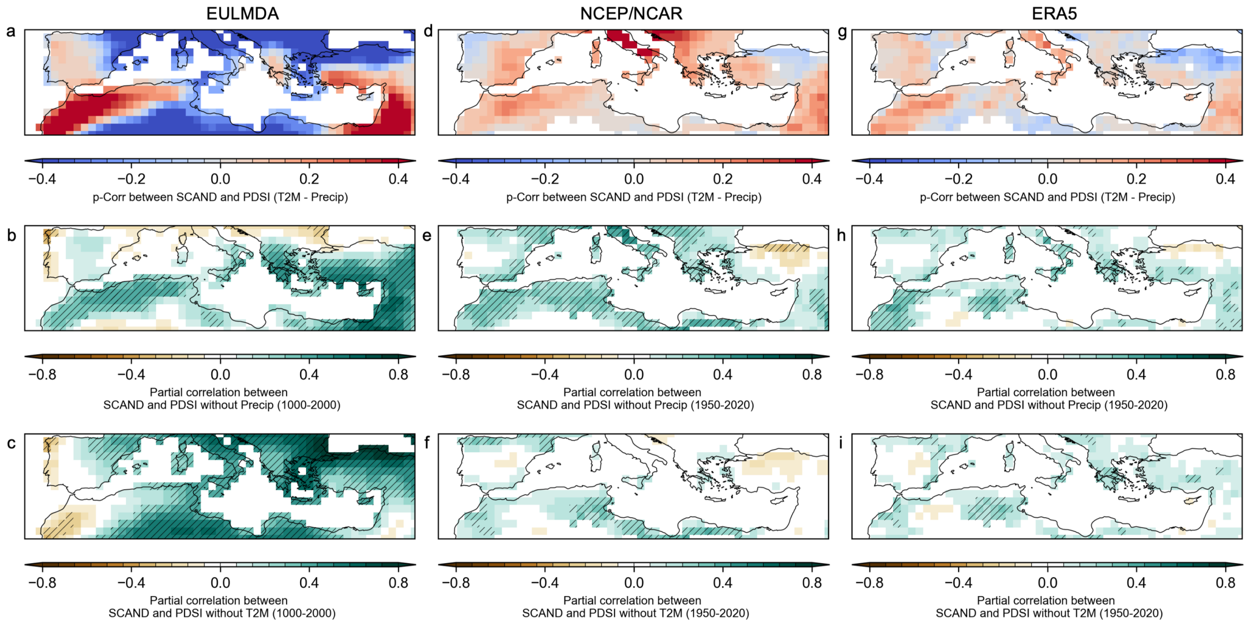

Supplementary Fig. 15 | Partial correlations between the summer SCAND index and Mediterranean JJA PDSI. (a–c) Results based on the EULMDA for 1000–2000 CE. (a) The difference in partial correlation coefficients of SCAND–PDSI between controlling the influence of precipitation (Precip, b) versus temperature (T2M, c), computed as (b minus c). (b–c) Partial correlation between the SCAND and PDSI after controlling for Precip (b) and T2M (c). **(d–f)** As in (a–c), but based on instrumental products over 1950–2020 CE with SCAND, T2M and Precip from NCEP/NCAR and PDSI from CRU. **(g–i)** As in (d–f), but based on SCAND, T2M and Precip from ERA5 (with CRU PDSI). Hatching indicates significance at the 95% confidence level.

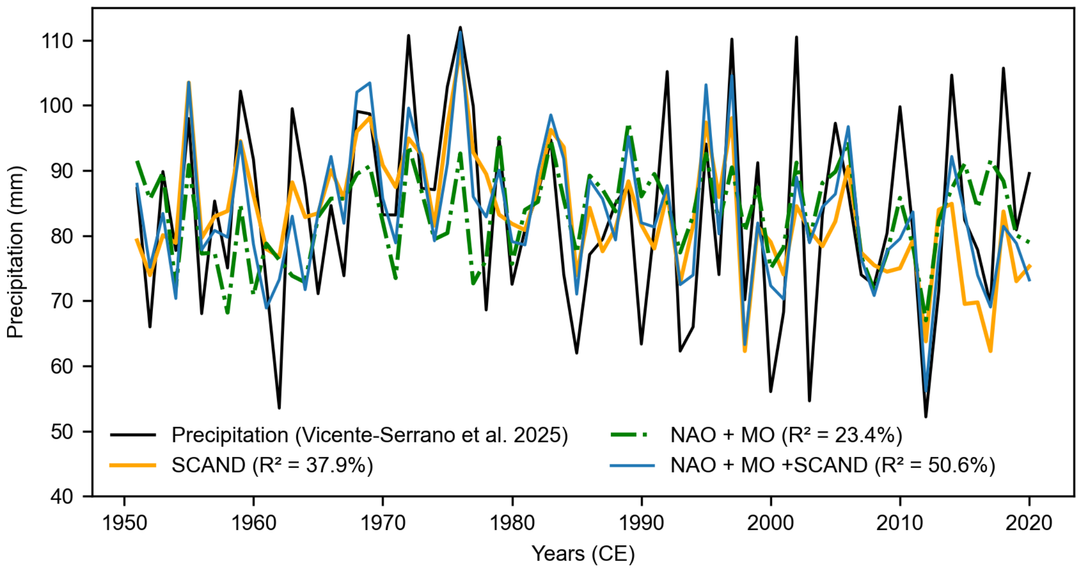

Supplementary Fig. 16 | Regression analyses of JJA Mediterranean precipitation against large-scale circulation indices (1951–2020 CE). Observed summer (JJA) precipitation (mm; black; from Vicente-Serrano et al., 2025) is compared with regression-based estimates using (i) the SCAND index (from NCAR/NCEP) alone (orange), (ii) NAO and MO index (green), and (iii) NAO, MO, and SCAND index (blue). The explained variances (R^2) are reported. Correlations between precipitation and the individual index are 0.62* for SCAND, 0.27* for NAO, and –0.41* for MO, highlighting the strong influence of SCAND relative to the other modes.

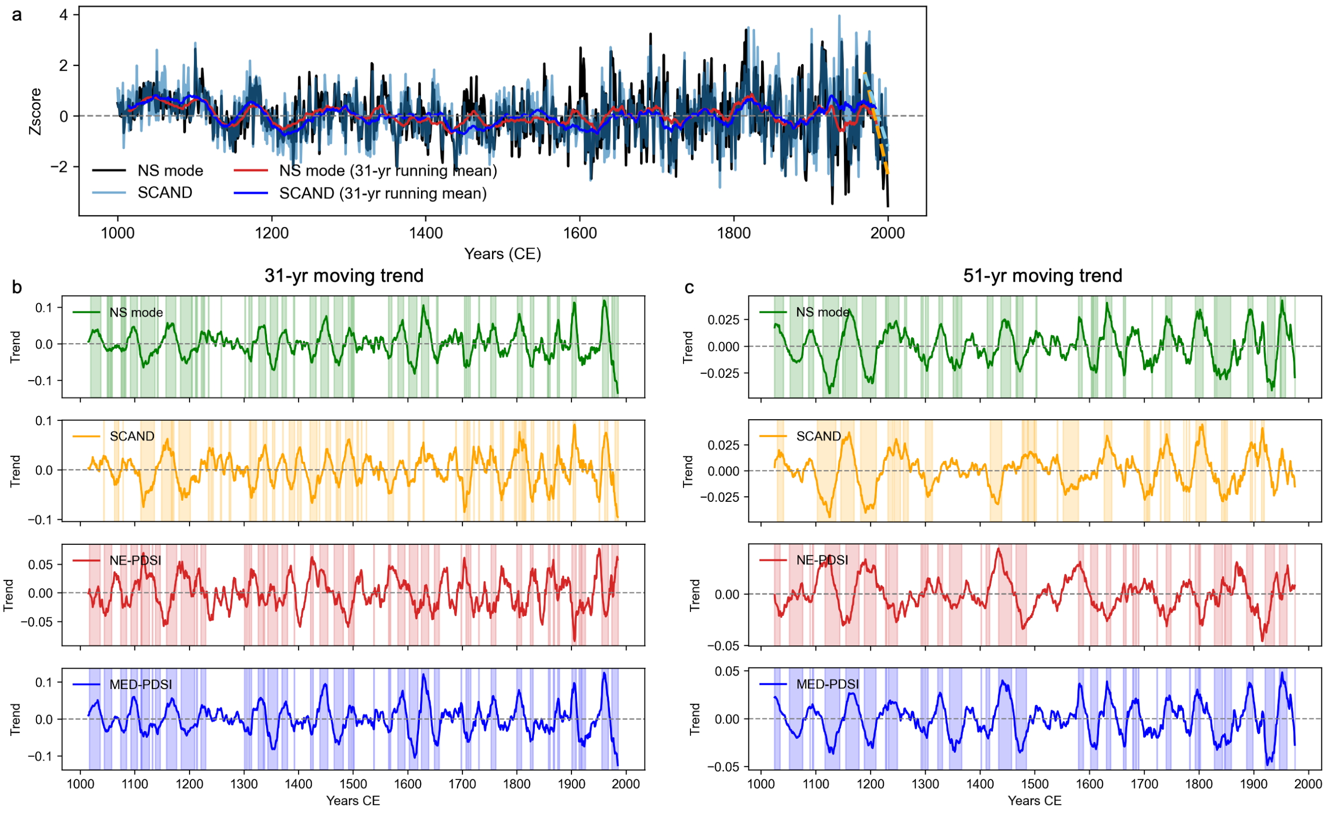

Supplementary Fig. 17 | Multidecadal coherence between SCAND and the north–south hydroclimate divergence over the past millennium. (**a**) Time series of the NS-mode index and the SCAND index in EULMDA over 1000–2000 CE, shown at annual resolution together with 31-year running means. (**b**) Moving 31-year trends for JJA SCAND index, NS-mode index, and regional mean PDSI for northern Europe (NE) and the Mediterranean (MED). (**c**) As in (b), but for moving 51-year trends. Shaded intervals highlight periods when the calculated trends are statistically significant at the 95% confidence level.

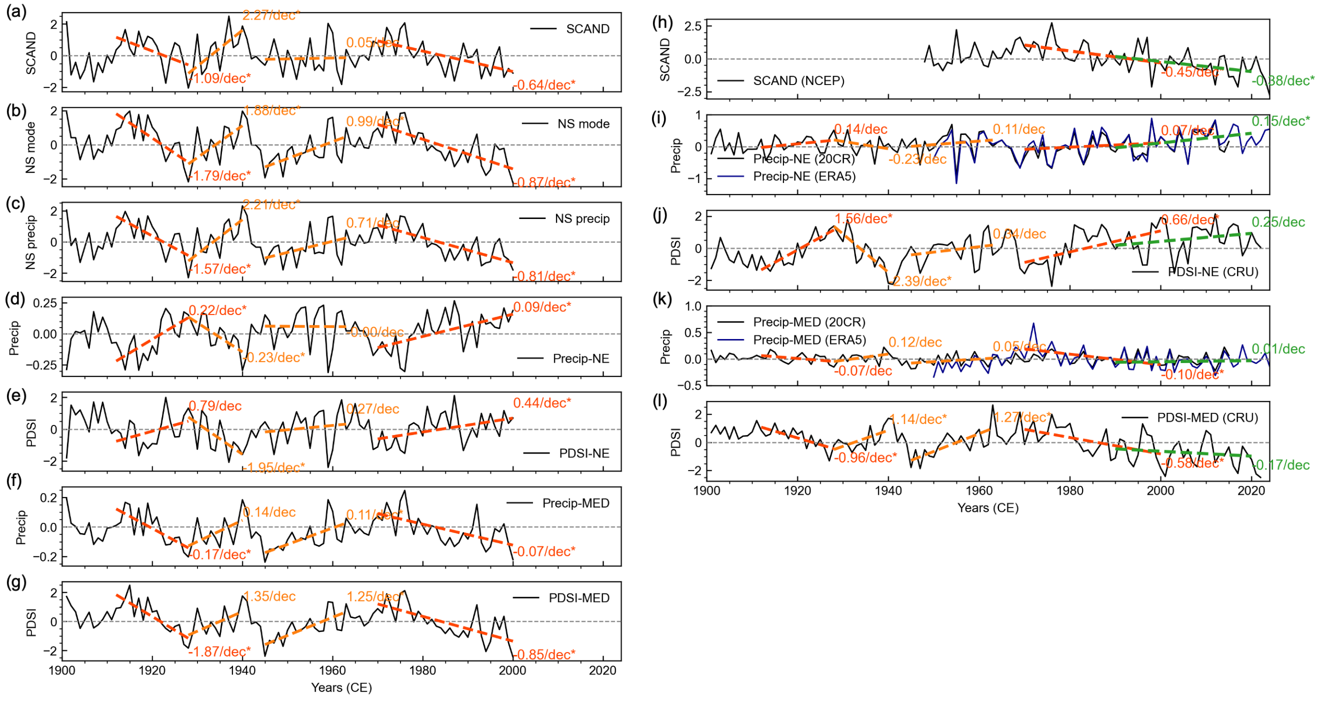

Supplementary Fig. 18 | Recent trends in SCAND and the north–south hydroclimate divergence. (a–g) Results based on EULMDA over 1901–2000 CE for the JJA SCAND index (a), NS-mode index (b), the north–south precipitation-gradient index (c), and regional mean precipitation (mm day^{-1}) and PDSI for northern Europe (NE; d–e) and the Mediterranean (MED; f–g). **(h–l)** Results based on instrument-based datasets over 1901–2024 CE, including SCAND from NCEP/NCAR over 1948–2024 CE (h), precipitation (mm day^{-1}) from ERA5 (1950–2024) and 20CR (1901–2015) for NE (i) and MED (k), and PDSI from CRU over 1901–2023 CE for NE (j) and MED (l). Black lines denote annual JJA anomalies relative to the 1960–1990 mean. Dashed lines indicate linear trends for 1912–1928 CE, 1928–1940 CE, 1945–1963 CE, 1970–2000 CE and 1990–2020 CE, with slope values reported in units per decade (asterisks denote $p < 0.05$). For precipitation in the instrumental datasets, trends over 1970–2000 CE and 1990–2020 CE are computed from ERA5, whereas trends for the other intervals are computed from 20CR.

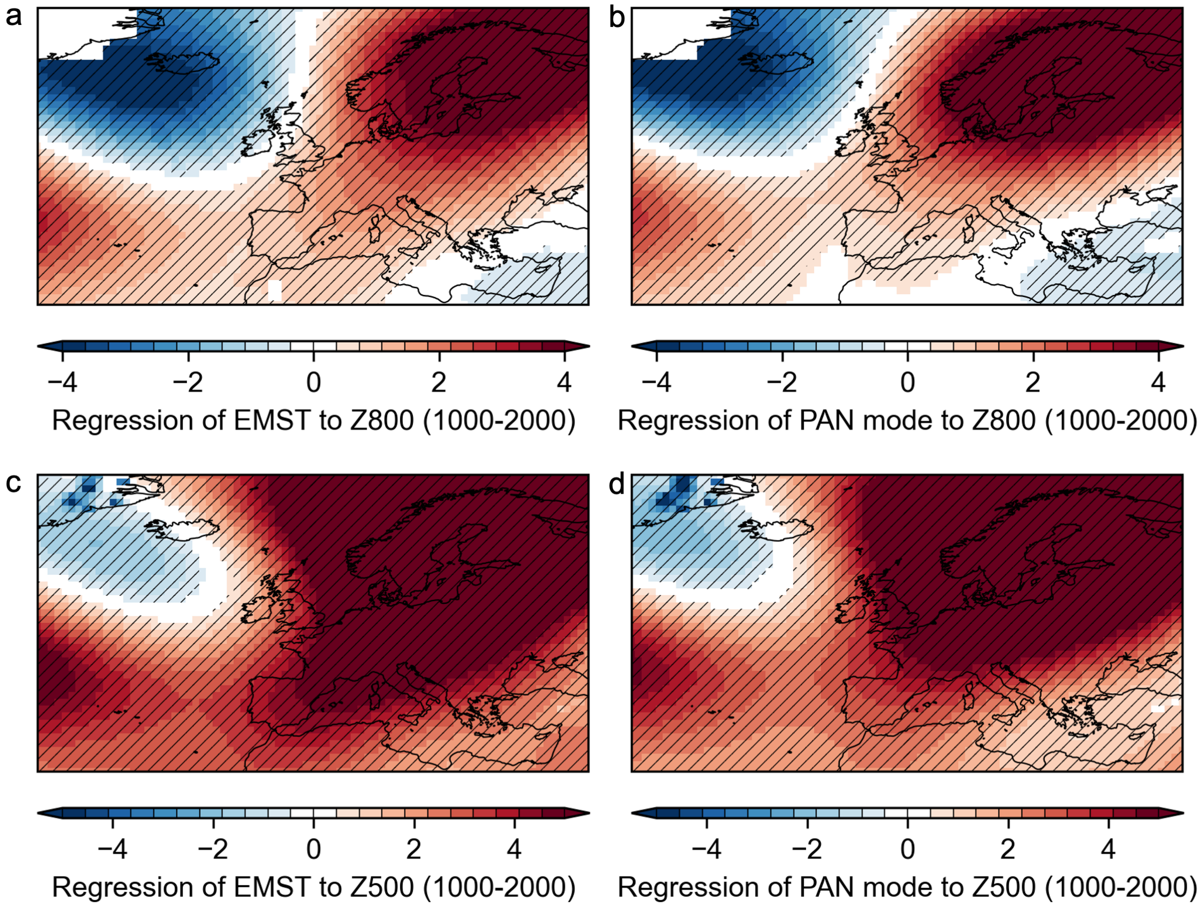

Supplementary Fig. 19 | Similar circulation signatures associated with EMST and the PAN mode. Regression of JJA geopotential height anomalies (m) at 800 hPa (Z800; a–b) and 500 hPa (Z500; c–d) onto European Mean Surface Temperature (EMST; a, c) and the PAN-mode index (b, d) in EULMDA over 1000–2000 CE. Hatching denotes areas where regression coefficients are statistically significant ($p < 0.05$).

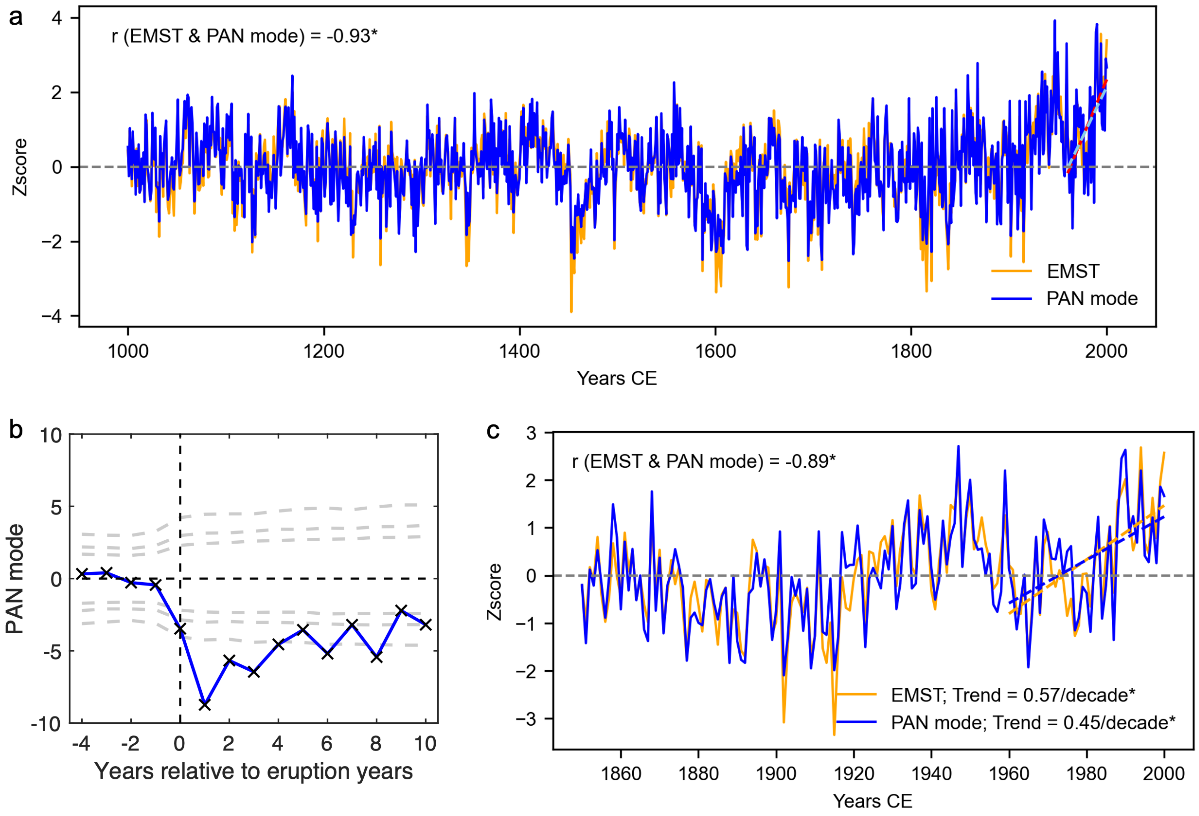

Supplementary Fig. 20 | PAN-mode variability, volcanic forcing, and recent warming. (**a**) Standardized time series of JJA European mean surface temperature (EMST; orange) and the PAN-mode index (blue) in EULMDA over 1000–2000 CE; the two indices are strongly anti-correlated ($r = -0.93$*). (**b**) Superposed epoch analysis of the PAN mode response to Northern Hemisphere and tropical volcanic eruptions (eruption year = 0; n = 23; eruptions listed in Supplementary Table 2). Solid line shows the composite mean, and dashed lines denote the 90% , 95% and 99% confidence envelope. (**c**) As in (a) but for 1900–2000 CE; linear trends over 1960–2000 CE are shown (trend values in the legend; asterisks denote $p < 0.05$).

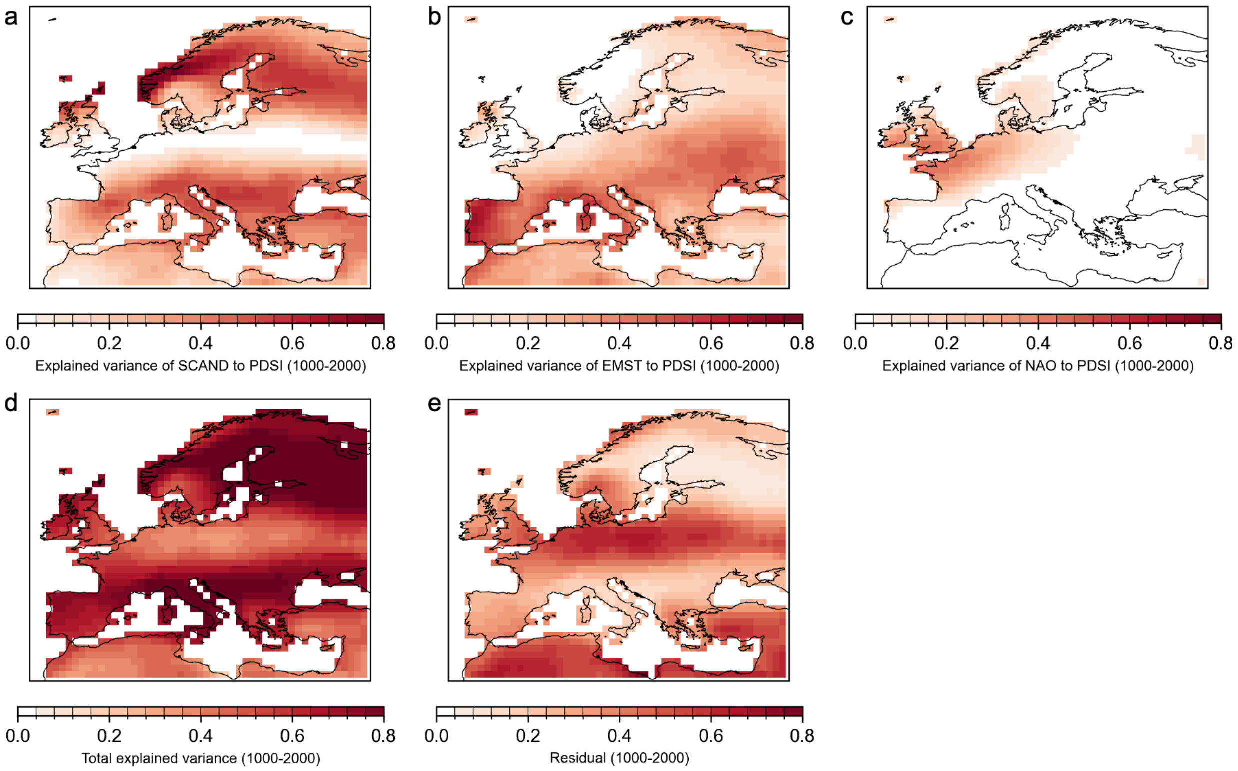

Supplementary Fig. 21 | Variance in European JJA PDSI explained by major large-scale drivers in EULMDA. Maps show the fraction of JJA PDSI variance explained over 1000–2000 CE by SCAND (a), European mean surface temperature (EMST, b), and the summer NAO (c). (d) shows the total variance explained by the three predictors combined, and (e) shows the residual variance, representing the unexplained portion of PDSI variability.

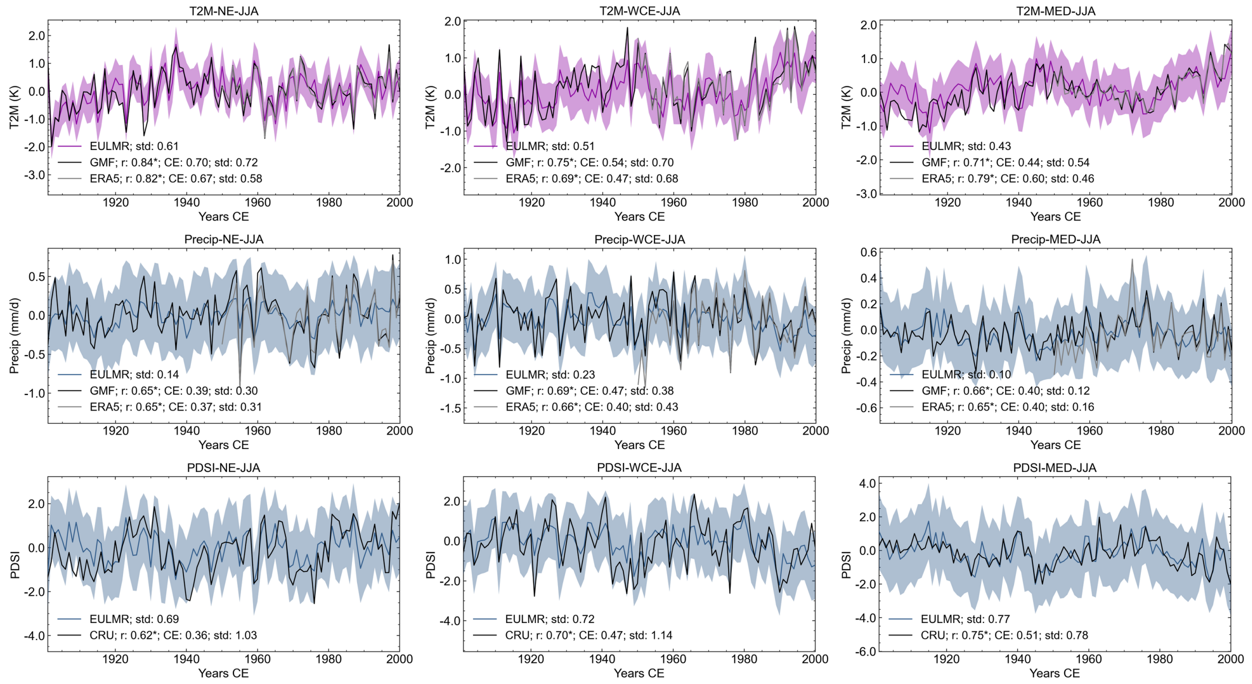

Supplementary Fig. 22 | Regional-mean validation of EULMDA against instrumental products. Regional-mean JJA anomalies of near-surface temperature (T2M; top row), precipitation (Precip; middle row), and PDSI (bottom row) for northern Europe (NE; left column), western–central Europe (WCE; middle column), and the Mediterranean (MED; right column). EULMDA is shown as the reconstruction ensemble mean with the ensemble spread (shading), and is compared with instrument-based reanalysis over 1901–2000 CE, except for ERA5 for the period 1950–2000 CE (GMF and ERA5 for T2M and Precip; CRU and Dai for PDSI, as labeled). Correlation coefficients (r), coefficient of efficience (CE) and standard deviations (std) for each reanalysis comparison are reported.

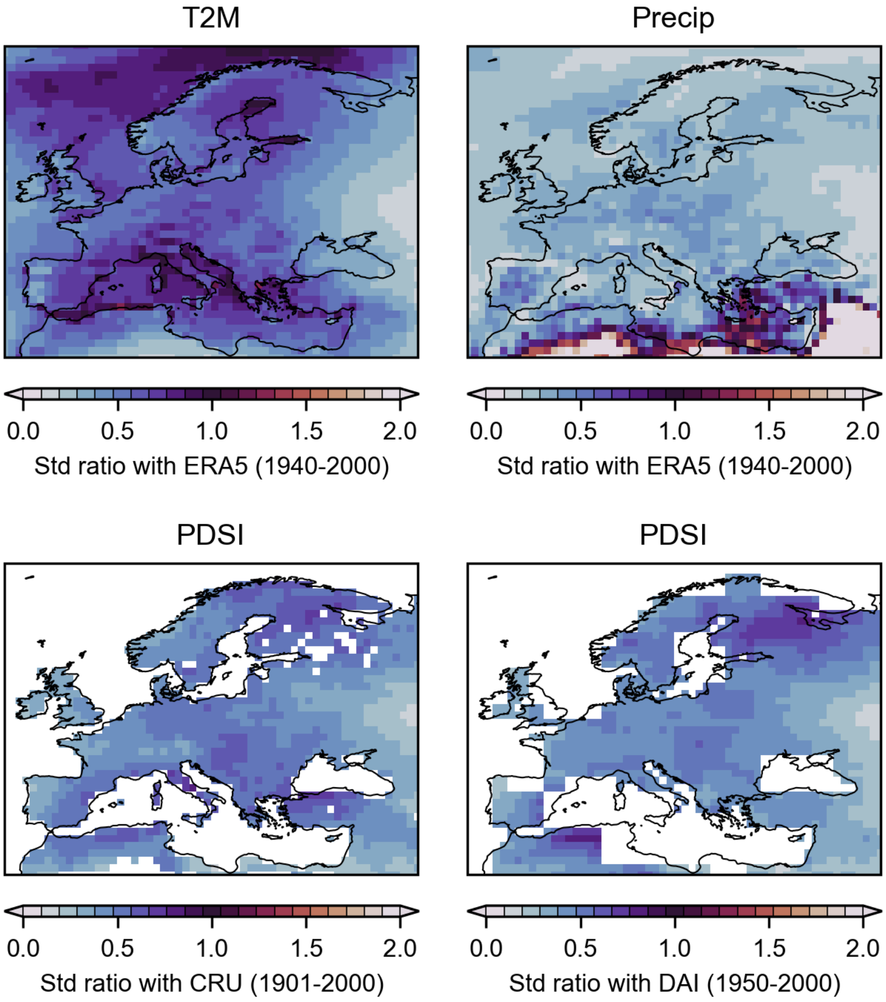

Supplementary Fig. 23 | Ratios of standard deviation in EULMDA to that in instrumental products. Maps show the ratio of the interannual standard deviation in EULMDA to that in instrument-based datasets for JJA near-surface temperature (T2M), precipitation (Precip) over 1940–2000 CE (against ERA5), and PDSI (against CRU for 1901–2000 CE and Dai for 1950–2000 CE). Values below 1 indicate reduced interannual variance in EULMDA relative to the reference dataset (variance damping), whereas values above 1 indicate larger variance in EULMDA.

Supplementary Tab. 1 | Earth system model simulations used as the prior ensemble in EULMDA. In our offline DA, particles are defined as all available simulated states (simulated years × ensemble members) from each model.

Prior	Number of Particles	Number of members	Simulated period
CESM-LME	12012	12	850–1850
ACCESS-ESM1	1000	1	850–1849
MIROC-ES2L	1000	1	850–1849
MRI-ESM2-0	1000	1	850–1849
CESM2	16500	100	1850–2014

Supplementary Tab. 2 | European volcanic eruptions used in Supplementary Fig. 20b. We include only tropical and Northern Hemisphere eruptions (latitude ≥ 30ºS) with stratospheric sulfur injection (SSI; Tg S) exceeding 6 Tg.

Eruptive years (CE)	SSI
**1883**	9.34
**1835**	9.48
**1831**	12.98
**1815**	28.08
**1809**	19.26
**1783**	20.81
**1695**	15.74
**1640**	18.68
**1600**	18.95
**1595**	8.87
**1585**	8.51
**1458**	32.98
**1453**	9.97
**1345**	15.11
**1286**	15.06
**1276**	11.53
**1257**	59.42

**1230**	23.78
**1191**	8.53
**1182**	10.05
**1171**	18.05
**1108**	19.16
**1028**	7.78

Supplementary Tab. 3 | Evaluation metrics. The ranges of metrics over all European land grid cells (10ºW–30ºE, 40ºN–73ºN; for SLP, the surrounding sea area is also included), together with the fraction of grid cells exceeding selected thresholds.

**Variable**	**Reference**	**r range**	**% grid r > 0.5**	**MSESS range**	**% grid MSESS > 0.3**
SLP	ERA5	0.07–0.65	36%	-0.04–0.32	10%
T2M	ERA5	0.3–0.8	91%	0.03–0.62	78%
Precip	ERA5	-0.07–0.72	37%	-0.13–0.38	3%
PDSI	CRU	0.17–0.71	59%	-0.06–0.48	32%
PDSI	Dai index	0.15–0.73	71%	-0.07-0.50	47%
