# Supplementary material for: Scandinavian pattern and temperature changes shape European summer droughts over the past millennium
Source: Nat Commun. 2026 Apr 27;17:7332. doi: 10.1038/s41467-026-72385-w (PMC13402590; doi:10.1038/s41467-026-72385-w)
Supplement: Supplementary file 2 — Transparent Peer Review file [file 41467_2026_72385_MOESM2_ESM.pdf]

nature portfolio

Peer Review File

Scandinavian pattern and temperature changes shape European summer droughts over the past millennium

Corresponding Author: Dr Huihong Xue

This file contains all reviewer reports in order by version, followed by all author rebuttals in order by version.

Attachments originally included by the reviewers as part of their assessment can be found at the end of this file.

Version 0:

Reviewer comments:

Reviewer #1

(Remarks to the Author)
The paper describes an offline data assimilation approach of proxy data into a set of climate model simulations, providing a climate data set for Europe for the last millennium. In this data set the authors analyse hydroclimatic variability in Europe and relate this to atmospheric circulation variability such as the SCAND mode, which describes Scandinavian blocking and manifests as a pressure dipole. They further partition drought variability and address dynamic and thermodynamic effects. The paper is interesting and relevant, scientifically sound and generally well written. However, there are some comments that the authors should take into account.
Methods: The core of the paper, the data set, is not described well enough. It would be good to have a bit more information already in the main part. But even when reading the method section, I miss a precise description of the method. How were the weights calculated? How are model and observation error covariances (diagonal?) taken into account? Are there assumptions of gaussianity somewhere in the procedure? How do you deal with model biases (other than using five ESMs), etc.? For the particle filter, the reader is referred to a paper by Dalaiden et al. (2021) which mainly refers to a paper by Dubinkina et al. I doubt that many readers would go all the way.
Data set: Likewise, I would appreciate a bit more information on the state vector. I see that the focus is on June-August averages, but then also spring is mentioned. The proxy system models requires monthly data, so arguably the output is monthly?
Evaluation: The data set seems well evaluated and it is compared against many different product. However, it is not clear to what extent these data sets are independent. This concerns both the assimilated observations (N-TREND and PAGES-2k are used by many) and the priors used. Could you comment? Perhaps independent evaluations could be performed. Could you evaluate the data set in the proxy space using independent proxy and PSMs? Since you mention spring, it would be interesting to also comment on the evaluation for spring.
The evaluations are almost exclusively quantified using correlations (and standard deviations are given), but correlations can be deceiving. Could you also use other standard measures (MSESS, RMSE, bias, etc.)?
Interpretations: I generally agree with the interpretation. The authors also mention soil moisture feedbacks and mention that these could affect southern Europe. It would be interesting whether the regions affected by soil moisture feedbacks change over the course of the millennium. Would the construction of the data set and the PSMs even allow this or is this sort of stationarity built in?
The paper remains climatological. A sentence on impacts on society or ecosystems (fire) might be interesting.
Minor:
L. 30: Is this referring to spatio-temporal variability? Or the temporal variability of continentally averaged drought?
L. 60: This seems to be the definition of summer in this paper, but this sentence seems to discuss literature. Perhaps state the season definition more explicitly.
L. 83: Here some information in the seasonality of the signal in proxies might be useful, as well as a time series figure on the number. I know this is all in the supplement, and the paper should not be too long. But perhaps consider moving a sentence or two here.
L. 103: Need to refer to IPCC regions?
L. 116: And how about 20CRv3?
L. 83: These are 32 years, not 30.
L. 246: Southern or more southeastern?

L. 593ff: Four closing brackets are missing in tis para
Fig. 1: What is the shading?
Generally there are quite many acronyms (EMST, PAN etc.). Could they be reduced?

Reviewer #2

(Remarks to the Author)
see attached review

Reviewer #3

(Remarks to the Author)
In this study, Xue et al. investigate the spatiotemporal variability and associated atmospheric drivers of European summer hydroclimate by developing a new data assimilation–based reconstruction, the European Last Millennium Data Assimilation (EULMDA). The authors systematically evaluate the performance of EULMDA against instrumental observations, reanalysis datasets, and other DA-based products. Using empirical orthogonal function analysis, they identify two dominant modes of summer PDSI variability: a north–south dipole mode linked to the Scandinavian pattern, and a continent-wide mode associated with surface warming. The study further explores the implications of these two modes for recent European hydroclimate changes, particularly over the Mediterranean region. Overall, the manuscript is clearly organized and comprehensive in scope, offering some new insights. However, several major concerns remain that should be carefully addressed before the manuscript can be considered further.

Major comments
1. The study presents a new data assimilation–based reconstruction of European hydroclimate, which has been evaluated against instrumental observations, reanalysis, and other DA products, as shown in Fig. 1, Extended Data Figs. 2–4, and Supplementary Figs. 1–2. However, these evaluations mainly demonstrate significant correlations between EULMDA and other datasets. I recommend that the authors further clarify the added value and necessity of developing this new DA product — for instance, does it outperform the prior (i.e., CESM2 simulations here) and other DA reconstructions during the instrumental period?

2. The reconstruction exhibits a notable increase in climate variability (Supplementary Fig. 5). This raises a concern that the identified PDSI modes and their associated atmospheric circulation patterns may be dominated by the more recent centuries, particularly after 1600 CE or even 1850 CE. I suggest that the authors provide the principal component time series of the two leading modes (are Extended Data Figs. 6b and 6c the PC series of the NS mode?), which would help to assess whether these patterns are period dependent. Moreover, the role of these two modes in recent European hydroclimate variability could be more directly identified from the PC series.

3. The reconstruction also exhibits a smaller overall amplitude compared to previous paleoclimate data assimilation products, particularly over the NE and WCE regions. This may suggest a systematic bias that should not be overlooked — especially if the variability during the instrumental period is also underestimated. I recommend that the authors further investigate this issue.

4. For the first mode (i.e., the NS mode), the authors attribute it primarily to the SCAND pattern. However, according to Fig. 3e and previous studies (e.g., Bladé et al., 2012), the summer NAO can also produce a north–south dipole–like effect on European precipitation. The manuscript only considers the influence of the NAO on the second, pan-European mode, but its potential contribution to the NS mode also deserves further consideration.
Reference: Bladé, I., Liebmann, B., Fortuny, D. et al. Observed and simulated impacts of the summer NAO in Europe: implications for projected drying in the Mediterranean region. Climate Dynamics, 39, 709–727 (2012).

5. For the second mode (i.e., the PAN mode), the authors attribute it mainly to the European Mean Surface Temperature (EMST), but the corresponding surface temperature field is not shown. Is the temperature anomaly also continent-wide, and how does the EMST drive or relate to the associated SLP pattern?

6. Correlation is a key diagnostic metric in this study. However, when comparing Fig. 2d (regression of SCAND to SLP during 1000–2000) and Supplementary Fig. 8b (regression of PAN mode to SLP during 1000–2000), the two patterns appear quite similar. This raises the question of whether their correlation might even exceed that between Fig. 2d (regression of SCAND to SLP during 1000–2000) and Fig. 2c (regression of NS mode to SLP during 1000–2000). Could the authors quantify or comment on this potential similarity?

Minor comments:
1. On what timescale are the correlation coefficients and explained variances calculated? Specifically, were the values shown in the relevant figures and texts derived from filtered or unfiltered data? Please specify.
2. For the related figures (i.e., Figs. 1–3, Extended Data Figs. 2-5, 9, Supplementary Figs. 1-2, 4, 7-9), please add more color bar values to improve clarity and facilitate interpretation.
3. Please specify the regional partitions shown in Extended Data Fig. 1.
4. Lines 224: In Extended Data Fig. 7a, please indicate how many volcanic eruptions were included in the calculation and list which ones were used.

5. Lines 227: In Extended Data Fig. 7b, the authors attribute the increase in EMST during 1960–2020 to anthropogenic forcing. However, its amplitude appears comparable to that of the 1910–1950 period.

Version 1:

Reviewer comments:

Reviewer #1

(Remarks to the Author)
This is the revised version of a paper that I have reviewed previously. Although I had many comments, most of them were about clarifications of the methodology, which was poorly described and only few comments about the scientific findings and their interpretations. I am pleased by the authors' detailed responses to my comments, and I confirm that they are also reflected in changes in the manuscript. There are no new points I would like to raise, I am therefore happy with the manuscript. It is a relevant addition to the literature both with respect to climate reconstructions and with respect to atmospheric circulation variability and drought processes.

Reviewer #2

(Remarks to the Author)
My comments have been addressed well. I recommend publication of the manuscript. I congratulate the authors on this high-quality study.

Reviewer #3

(Remarks to the Author)
I would like to commend the authors for their thorough and diligent revisions. The new analyses strengthen the overall results and provide valuable insights that will be of broad interest to the journal’s readership. However, there are several comments that would benefit from further clarification, including concerns regarding the choice of model simulations and data processing details.

Specific comments:
1. For the period 1850–2014, it is unclear why models that provide continuous coverage from 850 to 1850 simulations (e.g., ACCESS-ESM1, MIROC-ES2L, and MRI-ESM2-0) were not used, while CESM2—without 850–1850 simulations—was selected instead.

2. For the first response, time coverage of CESM1-LME is given as 1850–2000, while it’s described as 850–1850 in the Methods. Is this a typographical error?

3. For the second response (i.e., Supplementary Fig. 11), it may be helpful to use 1000–1500 and 1501–2000 as two subperiods for the EOF analysis. The results are unlikely to differ substantially, but this division may be more intuitive to interpret.

4. For the large-ensemble simulations (i.e., CESM-LME and CESM2), were the ensemble members averaged before or after data assimilation? Please clarify. If they were averaged prior to assimilation, the resulting field would primarily represent externally forced variability, which may contribute to the variance damping issue noted in the third response.

5. In the revised manuscript, the authors added geopotential height as a variable. I was wondering whether the 800 hPa level is obtained through interpolation, as model outputs are typically available at standard levels such as 850 or 700 hPa.

Version 2:

Reviewer comments:

Reviewer #3

(Remarks to the Author)
The authors have satisfactorily addressed my comments. I have no further concerns.

Open Access This Peer Review File is licensed under a Creative Commons Attribution 4.0 International License, which permits use, sharing, adaptation, distribution and reproduction in any medium or format, as long as you give appropriate credit to the original author(s) and the source, provide a link to the Creative Commons license, and indicate if changes were made.
In cases where reviewers are anonymous, credit should be given to 'Anonymous Referee' and the source.
The images or other third party material in this Peer Review File are included in the article's Creative Commons license, unless indicated otherwise in a credit line to the material. If material is not included in the article's Creative Commons license and your intended use is not permitted by statutory regulation or exceeds the permitted use, you will need to obtain permission directly from the copyright holder.
To view a copy of this license, visit https://creativecommons.org/licenses/by/4.0/

Response to Reviewer's comments

The authors thank the reviewers for their very helpful comments and suggestions. We here provide a point-by-point response to all reviewers. The reviewers' comments are given in black, and our responses are given in blue. We refer to line numbers in our revised manuscript.

REVIEWER COMMENTS

Reviewer #1 (Remarks to the Author):

The paper describes an offline data assimilation approach of proxy data into a set of climate model simulations, providing a climate data set for Europe for the last millennium. In this data set the authors analyse hydroclimatic variability in Europe and relate this to atmospheric circulation variability such as the SCAND mode, which describes Scandinavian blocking and manifests as a pressure dipole. They further partition drought variability and address dynamic and thermodynamic effects. The paper is interesting and relevant, scientifically sound and generally well written. However, there are some comments that the authors should take into account.

We thank the reviewer for the positive evaluation of our manuscript. We have considered all comments and revised the manuscript accordingly; detailed responses are provided below.

Methods: The core of the paper, the data set, is not described well enough. It would be good to have a bit more information already in the main part. But even when reading the method section, I miss a precise description of the method. How were the weights calculated? How are model and observation error covariances (diagonal?) taken into account? Are there assumptions of gaussianity somewhere in the procedure? How do you deal with model biases (other than using five ESMs), etc.? For the particle filter, the reader is referred to a paper by Dalaiden et al. (2021) which mainly refers to a paper by Dubinkina et al. I doubt that many readers would go all the way.

We thank the reviewer for these comments. We have expanded the description of the data set in the main text (Lines 76–87) and added a detailed description of our DA implementations in the

“Paleoclimate data assimilation” section of the Methods. Below we answer the specific questions raised by the reviewer.

(1) How were the particle weights calculated?

At each assimilation step (annual), the weight of particle i is given by a Gaussian likelihood based on the misfit between the proxy observations and the corresponding model pseudo-observations (i.e. the estimate of the variable measured derived from model results):

$$p(d|\psi) = K^{-1}exp\left[-\frac{1}{2}\big(d - H(\psi)\big)^T C^{-1}\big(d - H(\psi)\big)\right]$$

where K^{-1} is a normalization constant, d is the proxy observation, ψ is model state, H is the observational operator, which maps the prior to the observational phase space, $H(\psi)$ is the model pseudo-observation, and C is the observation-error covariance which is assumed here to be diagonal.

(2) How are model and observation error covariances (diagonal?) taken into account?

The observation-error covariance matrix is taken to be diagonal. We assume that the instrumental observation used to calibrate the proxy record is unbiased, so the observation-error in our case includes PSM-related error and representativeness error. For each proxy, this error is estimated by taking the standard deviation of the residuals from the linear regression-based PSMs; this includes representativeness error because the PSMs are calibrated using the climate data on the 1° grid rather than the original grid.

Our methodology does not require explicitly specifying a model error covariance matrix; instead, model error is represented by the prior ensemble (particles) spread.

(3) Are there assumptions of gaussianity somewhere in the procedure?

Gaussian is only assumed in the likelihood and in the specification of observation errors. However, the particle filter does not assume the distribution of climate variables (states) as Gaussian. The prior is given by a discrete ensemble, and each particle follows the fully nonlinear model dynamics, derived from the Earth System Models.

(4) How do you deal with model biases (other than using five ESMs), etc.?

In addition to using five different ESMs to reduce dependence on any single model’s performance, we perform the assimilation in anomaly space, removing the mean bias of the models.

Data set: Likewise, I would appreciate a bit more information on the state vector. I see that the focus is on June-August averages, but then also spring is mentioned. The proxy system models requires monthly data, so arguably the output is monthly?

In our implementation, the state vector is monthly, whereas the assimilation is performed at an annual time step, i.e., the prior ensemble is updated once per year. Proxy information therefore enters the assimilation annually, and the yearly update is applied to the full monthly state through the cross-variable and cross-month relationships represented in the prior ensemble. More specifically, our proxy system models (PSMs) do not use monthly proxy–climate relationships. Instead, each tree-ring record is calibrated against temperature and/or precipitation averaged over one of three candidate windows: annual (ANN), June–August (JJA), or March–September (MAMJJAS; i.e., the growing season of trees). The optimal window is selected using the Bayesian information criterion. As a result, some records primarily constrain summer variability, whereas others constrain the growing season or the annual mean.

Although the vector state is monthly, the effective observational constraint is strongest for warm-season averages. Consequently, reconstruction skill is highest for JJA means, while skill for individual months or for seasons outside the proxy-sensitive windows is generally weaker and may not always be statistically significant.

We have added descriptions of the state vector in the data assimilation section (Lines 400–404) and clarified the PSM input data (Lines 468–469).

Evaluation: The data set seems well evaluated and it is compared against many different product. However, it is not clear to what extent these data sets are independent. This concerns both the assimilated observations (N-TREND and PAGES-2k are used by many) and the priors used. Could you comment?

Perhaps independent evaluations could be performed. Could you evaluate the data set in the proxy space using independent proxy and PSMs?

Since you mention spring, it would be interesting to also comment on the evaluation for spring.

The evaluations are almost exclusively quantified using correlations (and standard deviations are given), but correlations can be deceiving. Could you also use other standard measures (MSESS, RMSE, bias, etc.)?

We thank the reviewer for these constructive suggestions. We address each point below.

(1) Independence of the evaluation datasets

Our main evaluation compares the EULMDA fields with instrument-based gridded datasets (ERA5, 20CR, CRU and Dai PDSI). These datasets do not contain the assimilated tree-ring records. Apart from the exact grid cells where instrumental data were used to calibrate the proxy system models (PSMs), the comparison between EULMDA and these gridded observational fields is independent of the assimilated proxy network.

Regarding the prior, the ModE-RA product used for comparison is based on an ensemble of atmospheric simulations (i.e., ECHAM6), whereas the EULMDA prior is constructed from five different ESMs and does not include ECHAM6. Therefore, the ModE-RA prior is independent of the prior information used in EULMDA.

We agree that evaluation in proxy space using independent proxies and PSMs would be ideal. However, obtaining a sufficiently large and fully independent set of long tree-ring chronologies is difficult, because most publicly available long series are already included in PAGES2k or N-TREND. As an alternative, we added evaluations using long-term meteorological and documentary data that are entirely independent of the assimilated tree-ring network. Specifically, we now evaluate temperature and precipitation over 1700–2000 CE using the Alpine reconstruction of Casty et al., (2005), which blends early instrumental measurements with documentary information. EULMDA shows significantly positive correlations ($p < 0.05$) with Casty et al. (2005) across the Alpine region for both temperature and precipitation (Supplementary Fig 8a–b). In addition, we compare reconstructed JJA precipitation with the Paris station (48º52'N, 2º20'E) series (Slonosky, 2002) by extracting the nearest EULMDA grid cell. The correlation over 1700–2000 CE is $r = 0.42$ ($p < 0.05$), indicating that EULMDA captures a substantial fraction of interannual variability at this independent site (Supplementary Fig 8c). These new evaluations are described in "Validation of EULMDA climate reconstruction" (Lines 133–139).

(2) Evaluation for spring

Spring (MAM) is not the target season of the main reconstruction; it is used only in additional analysis testing cross-seasonal influences of the spring SCAND pattern on summer PDSI. The reconstructed spring SCAND index is significantly correlated with the instrument-based index ($r = 0.36$, $p < 0.05$; Supplementary Fig 14a), but less than for summer ($r = 0.70$, $p < 0.05$).

As additional information, we provide a broader spring evaluation here by comparing reconstructed MAM temperature, precipitation, sea-level pressure (SLP) and PDSI with ERA5 (temperature, precipitation, SLP) and CRU/Dai PDSI (PDSI; Fig R1). Spring correlations are generally positive over large parts of Europe, though somewhat weaker and more spatially patchy than for JJA, suggesting that large-scale spring circulation and hydroclimate anomalies are reasonably captured but with reduced skill relative to summer.

(3) Additional evaluation metrics beyond correlation

As suggested, we now compute the Mean Square Error Skill Score (MSESS) for all evaluated variables. MSESS broadly mirrors the correlation patterns: it is positive over most of Europe, with negative values mainly over North Africa (Supplementary Fig. 2). These results have been mentioned in main text (Lines 97–98) and described in the Supplementary Text (Lines S37–S39). The mean bias and root-mean-square error (RMSE) of absolute values are not directly comparable in our framework because the assimilation is performed in anomaly space.

References

Casty, C., Wanner, H., Luterbacher, J., Esper, J., & Böhm, R. (2005). Temperature and precipitation variability in the European Alps since 1500. *Int. J. Climatol.*, *25*(14), 1855–1880.

Slonosky, V. C. (2002). Wet winters, dry summers? Three centuries of precipitation data from Paris. *Geophys. Res. Lett.*, *29*(19), 1895. doi:10.1029/2001GL014302

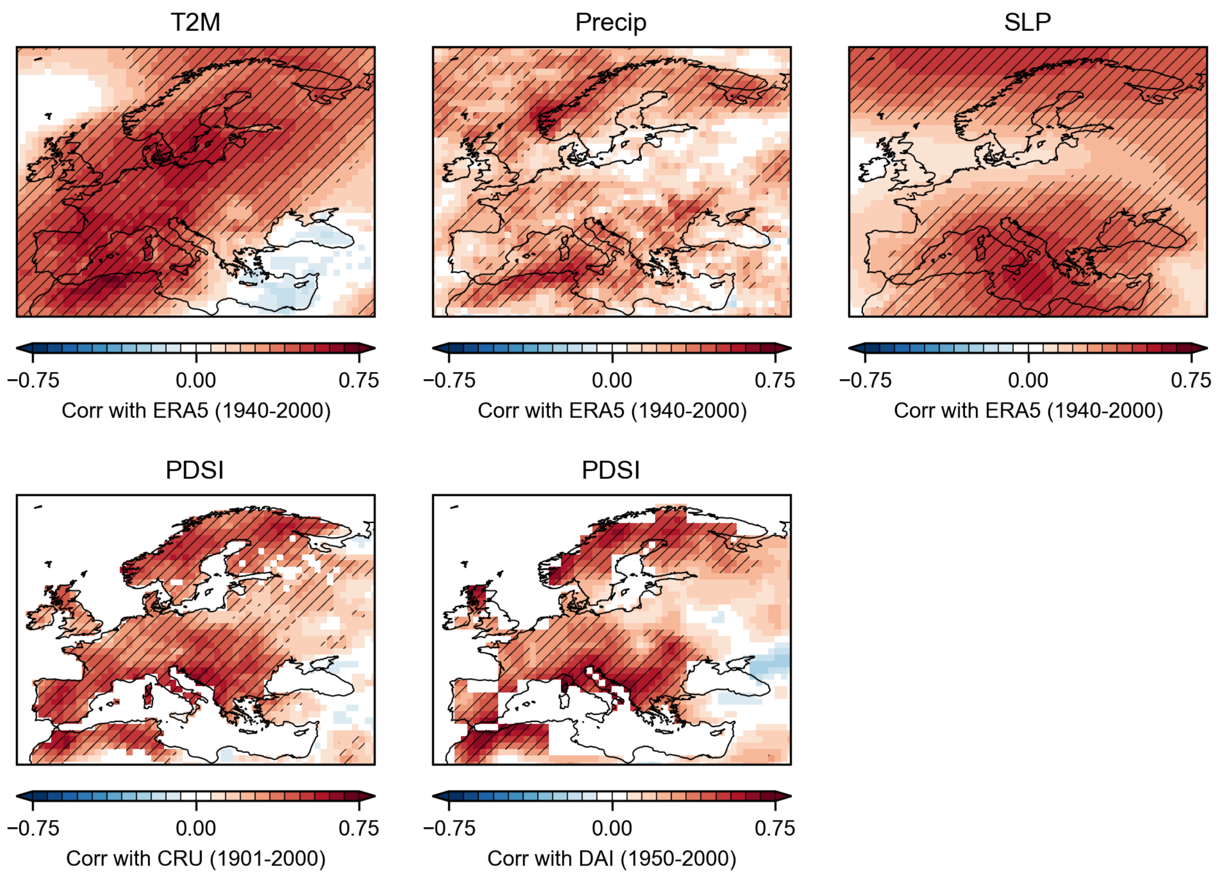

Figure R1. Evaluation of the spring (MAM) EULMDA reconstruction. Spatial correlations between EULMDA and reference datasets for near-surface temperature (T2M), precipitation, and sea-level pressure (SLP) against ERA5 (1940–2000), and for PDSI against CRU (1901–2000) and Dai PDSI (1950–2000). Hatched areas denote correlations significant at $p < 0.05$.

Interpretations: I generally agree with the interpretation. The authors also mention soil moisture feedbacks and mention that these could affect southern Europe. It would be interesting whether the regions affected by soil moisture feedbacks change over the course of the millennium. Would the construction of the data set and the PSMs even allow this or is this sort of stationarity built in?

Soil–moisture (SM) feedbacks can indeed be an important mechanism for European summer hydroclimate variability. However, a robust quantification of SM–temperature and SM–precipitation feedbacks typically requires land-surface and boundary-layer variables (e.g., soil moisture, evapotranspiration/latent and sensible heat fluxes, radiation, and convective

diagnostics) (Dirmeyer, 2011; Miralles et al., 2012) and/or controlled model experiments, e.g., global land–atmosphere coupling experiment (GLACE)-type ensembles (Santanello Jr et al., 2011; Schär et al., 1999).

While our DA framework could in theory be extended to include land-surface variables, we do not expect such variables to be robustly constrained by our proxy network. Tree-ring records primarily constrain seasonal hydroclimate variability through their sensitivity to temperature and moisture availability. They do not directly observe net radiation or turbulent fluxes. Although tree-ring–based PSMs can, to some extent, be used in inverse mode with simple big-leaf models to infer aspects of carbon and water exchange (e.g., Boucher et al., 2014), implementing such an approach in practice is complex, and the resulting constraints on land–atmosphere coupling remain indirect. As a result, posterior updates for variables such as evapotranspiration or sensible heat flux would rely mainly on prior cross-covariances with temperature or precipitation, which are model-dependent. Thus, even if EULMDA shows good skill for reconstructed variables, it is difficult to disentangle what portion of any apparent feedback patterns reflects earth system model parameterizations versus the real-world land–atmosphere coupling. We therefore clarify in the revised manuscript that assessing the soil-moisture feedback is beyond the scope of EULMDA (Lines 343–345).

References

Boucher, É., Guiot, J., Hatté, C., Daux, V., Danis, P.-A., and Dussouillez, P. (2014). An inverse modeling approach for tree-ring-based climate reconstructions under changing atmospheric CO_2 concentrations, *Biogeosciences*, *11*, 3245–3258.

Dirmeyer, P. A. (2011). The terrestrial segment of soil moisture–climate coupling. *Geophys. Res. Lett.*, *38*(16). https://doi.org/10.1029/2011GL048268

Miralles, D. G., van den Berg, M. J., Teuling, A. J., & de Jeu, R. A. M. (2012). Soil moisture-temperature coupling: A multiscale observational analysis. *Geophys. Res. Lett.*, *39*(21). https://doi.org/10.1029/2012GL053703

Santanello Jr, J. A., Peters-Lidard, C. D., & Kumar, S. V. (2011). Diagnosing the sensitivity of local land–atmosphere coupling via the soil moisture–boundary layer interaction. *J. Hydrometeorol.*, *12*(5), 766–786.

Schär, C., Lüthi, D., Beyerle, U., & Heise, E. (1999). The soil–precipitation feedback: A process study with a regional climate model. *J. Clim.*, *12*(3), 722–741.

The paper remains climatological. A sentence on impacts on society or ecosystems (fire) might be interesting.

We have added a brief sentence in the final paragraph of the section "Insights into the drivers of European summer hydroclimate variability" to highlight socio-ecological implications of hot and dry summers (Lines 371–373).

Minor:

L. 30: Is this referring to spatio-temporal variability? Or the temporal variability of continentally averaged drought?

We refer to European spatiotemporal drought variability. We have clarified this.

L. 60: This seems to be the definition of summer in this paper, but this sentence seems to discuss literature. Perhaps state the season definition more explicitly.

We now state the season definition explicitly (Line 85)

L. 83: Here some information in the seasonality of the signal in proxies might be useful, as well as a time series figure on the number. I know this is all in the supplement, and the paper should not be too long. But perhaps consider moving a sentence or two here.

We now add a brief sentence summarizing the seasonality of proxies in the main text (Lines 81–83).

L. 103: Need to refer to IPCC regions?

IPCC regions are now referenced (Line 107), and the regional definitions are indicated in the caption of Supplementary Fig. 1a.

L. 116: And how about 20CRv3?

We now include a comparison with 20CRv3. The EULMDA-derived SCAND index correlates with the 20CRv3-derived SCAND index at $r = 0.63$ ($p < 0.05$) over 1850–2000 CE (Supplementary Fig. 6a–d).

L. 183: These are 32 years, not 30.

Corrected.

L. 246: Southern or more southeastern?

We intended southern (Line 343).

L. 593ff: Four closing brackets are missing in tis para

Corrected.

Fig. 1: What is the shading?

We now explain the shading explicitly in the caption. The Shading denotes the correlation coefficient.

Generally there are quite many acronyms (EMST, PAN etc.). Could they be reduced?

We have reduced acronym usage where possible, while keeping key terms that are repeatedly used.

Reviewer #2 (Remarks to the Author):

Review of

'Scandinavian pattern and temperature changes shape European summer droughts over the past millennium'

by Huihong Xue, Hugues Goosse, Quentin Dalaiden, Kristina Seftigen, Fabio Gennaretti, and Feng Shi

Recommendation: minor revisions

This manuscript presents the European Last Millennial Data Assimilation (EULMDA), a new reconstruction of European hydroclimate and its main drivers for the past millennium. It is based on off-line data assimilation with a particle filter that combines several hundred moisture and

temperature-sensitive tree-ring records with simulations from 5 climate models. This data assimilation approach is well established, and a comprehensive evaluation of the specific new implementation against instrumental data, which is part of the manuscript, has demonstrated good performance.

After providing a review of the current understanding of thermodynamic and dynamic processes contributing to European droughts, the study uses EULMDA to address open questions related to the variability and causes of European summer droughts. The main finding is that natural variability of the Scandinavian pattern, which is one of the dominant large-scale atmospheric pressure variability modes in the European-Atlantic sector, is a key driver for soil moisture north-south gradients across Europe and for soil moisture variability in the Mediterranean region, which acts in addition to are anthropogenically forced, temperature-dependent thermodynamic processes.

The study provides substantial new findings that are of interest for a wide group of readers, and it is methodologically sound and well written. I therefore recommend publication after the specific points listed below have been addressed.

We appreciate the reviewer's positive feedback and insightful comments on improving the manuscript. We provide point-by-point responses in the following.

Specific comments

1) It should be said in the introduction, in the evaluation section, and in the Fig.1 caption what the temporal resolution of EULMDA is, and whether the correlations in Fig. 1 are based on summer or annual means. These details become clear in the method section, but the information should also be given in the main text.

We have now stated in the Introduction that EULMDA is an annually resolved reconstruction targeting JJA conditions (Lines 77, 85), and we clarified in the evaluation sections and Fig. 1 caption that all correlations are computed using JJA-mean anomalies.

2) Lines 121-122: The first part of this sentence is not correctly constructed.

We have corrected the sentence to read “By contrast, the amplitude is more sensitive and becomes increasingly damped in earlier centuries (Supplementary Fig. 10), partly reflecting limited proxy constraints[47]”.

3) The regression maps in Figs. 2 and 3 are very informative. It should be noted that the regression of a time-dependent spatial field onto a time series, which is what these maps are, has a straightforward interpretation when the time series is used as a predictor for the spatial field. However, for instance in the case of the SLP regression maps, the conceptual understanding is that the field anomalies are the predictors for the time series. It is not immediately obvious how the regression maps are linked to this estimation problem.

It has been shown by Widmann (2005) that using the time expansion coefficient of the regression maps as the predictor for the time series (which is presumably what one has in mind when interpreting the regression maps) is equivalent to a 1-dimensional maximum covariance analysis (also known as Singular Value Analysis). It would be good to cite Widmann (2005) and clarify that using regression maps in the context of predicting the time series from the field has a sound theoretical foundation.

Please also note that the regression of a time-dependent spatial field onto Principal Component Scores calculated from this field (as in Figs. 2a and 3a) is proportional to the Principal Components Loadings (aka EOF patterns) (this is a special case of 1-dimensional Maximum Covariance Analysis).

Widmann, M., 2005. One-dimensional CCA and SVD, and their relationship to regression maps. Journal of Climate, 18(14), pp.2785-2792.

We thank the reviewer for this helpful clarification. Following this suggestion, we now add a new Methods subsection, "Regression and covariance analysis", to describe our regression set-up and clarify how the regression patterns are interpreted (Lines 563–575). We now cite Widmann (2005) and note that using the regression map's time expansion coefficient as the predictor is mathematically equivalent to a one-dimensional maximum covariance (or singular value) analysis between the field and the index, which provides a clear statistical basis for interpreting the regression maps in this predictive sense. We also clarify that when the index is a principal component of the field itself (as in revised Figs. 2a and 4a), the regression pattern is proportional to the corresponding EOF loading pattern.

4) Lines 171-174: The contribution of the SCAND trend 1970–2000 (and potentially also for periods with more recent end dates) to regional PDSI trends, and to the trend in the north-south

gradient of the PDSI should be quantified. For the regional trends this can be done by multiplying the SCAND trend during the reference period with the local PDSI on SCAND regression coefficients (i.e. with the regression maps) and dividing the result, which are the SCAND-driven local trends, by the actual local trends. For the north-south gradient this can be done by regressing the PDSI NS time series (i.e. the associated Principal Component Score) onto the SCAND time series, then multiplying with the SCAND trend, and dividing the result by the actual PDSI NS trend.

The contribution of the PAN trends could be quantified in the same way.

The authors could also consider applying this approach to the regional trends in precipitation, and to the trends in north-south precipitation gradients. How to define the latter requires some thought. Options are Principal Component Score time series, or using the time expansion coefficient of the regression map of precipitation on the PDSI PAN and NS timeseries (which is getting a bit convoluted).

Thank you for this suggestion. Following the reviewer's proposed approach, we now quantify the contribution of the SCAND trend over 1970–2000 CE and 1990–2020 CE to (i) regional PDSI trends, (ii) regional precipitation trends, and (iii) the trends in the north–south (NS) mode and (iv) corresponding NS precipitation-gradient index. These results are now reported in the section "Role of the Scandinavian pattern in past and present summer European hydroclimates" and shown in Supplementary Figs 13 and 18.

In addition, we also quantify the contribution of the European mean surface temperature (EMST) trend to the PAN-mode trend using the same regression-based approach, and report this in Lines 322–325.

The underlying methodology is described in the new Methods subsection "Regression and covariance analysis" (Lines 576–587).

5) Line 254: 'where has undergone' should be replaced by 'which had undergone'.

Corrected.

6) Line 256: Wrong citation format.

Corrected.

7) Lines 271-282: It is good that it is checked whether the relationships found in EULMDA are also present in instrumental data and reanalyses. However, it would be informative to do this not only for the Mediterranean region but also for the main relationships for the whole European region, most importantly for the link between the SCAN pressure and NS soil moisture patterns.
We thank the reviewer for this suggestion. We have now extended the instrument/reanalysis-based analysis to both northern Europe and the Mediterranean, which are most relevant to the SCAND-driven north–south dipole.

Instrument-based datasets broadly support the relationships found in EULMDA, with SCAND-related circulation anomalies corresponding to opposite-signed hydroclimate responses between the north and south (PDSI and precipitation). These results are now included in the section "Role of the Scandinavian pattern in past and present summer European hydroclimates" (revised Fig. 3 and Supplementary Fig. 18).

Reviewer #3 (Remarks to the Author):

In this study, Xue et al. investigate the spatiotemporal variability and associated atmospheric drivers of European summer hydroclimate by developing a new data assimilation–based reconstruction, the European Last Millennium Data Assimilation (EULMDA). The authors systematically evaluate the performance of EULMDA against instrumental observations, reanalysis datasets, and other DA-based products. Using empirical orthogonal function analysis, they identify two dominant modes of summer PDSI variability: a north–south dipole mode linked to the Scandinavian pattern, and a continent-wide mode associated with surface warming. The study further explores the implications of these two modes for recent European hydroclimate changes, particularly over the Mediterranean region. Overall, the manuscript is clearly organized and comprehensive in scope, offering some new insights. However, several major concerns remain that should be carefully addressed before the manuscript can be considered further.
We thank the reviewer for this constructive assessment. We have carefully addressed the major concerns raised and provide detailed point-by-point responses below.

Major comments

1. The study presents a new data assimilation–based reconstruction of European hydroclimate, which has been evaluated against instrumental observations, reanalysis, and other DA products, as shown in Fig. 1, Extended Data Figs. 2–4, and Supplementary Figs. 1–2. However, these evaluations mainly demonstrate significant correlations between EULMDA and other datasets. I recommend that the authors further clarify the added value and necessity of developing this new DA product — for instance, does it outperform the prior (i.e., CESM2 simulations here) and other DA reconstructions during the instrumental period?

We thank the reviewer for this comment.

First, we compare summer temperature from CESM1-LME and CESM2-LE with 20CR over 1850–2000 CE, and we compare the other three prior simulations (ACCESS-ESM1-5, MIROC-ES2L, MRI-ESM2-0) with EULMDA over 1000–1849 CE. As expected for free-running climate model simulations, correlations between the priors and observation-based datasets are generally weak and often not significant (Fig. R2), reflecting the fact that the models do not reproduce the observed internal variability with the correct phase.

Second, we compare hydroclimate variables from EULMDA with PHYDA and ModE-RA. Last Millennium Reanalysis (LMR) is not included here because it mainly provides annual-mean fields, which makes a direct comparison with our JJA reconstruction difficult. Relative to CRU PDSI over 1901–2000 CE, EULMDA and PHYDA (which does not provide precipitation) show broadly comparable skill, with EULMDA performing slightly better in parts of the Mediterranean and high-latitude northern Europe (Supplementary Fig. 7i). For precipitation, we compare EULMDA with ModE-RA (which does not provide PDSI): ModE-RA shows higher instrumental-period skill, consistent with its assimilation of instrumental station observations in the 19th–20th centuries, whereas EULMDA is constrained only by tree-ring information (Supplementary Fig. 7j; Supplementary Text Lines S97–103).

Importantly, the main value of EULMDA does not lie in uniformly outperforming previous DA products for all variables, but in providing a hydroclimate-focused, dynamically consistent reconstruction tailored to European summer drought. EULMDA jointly reconstructs summer temperature, precipitation, PDSI and atmospheric circulation within a single framework, ensuring physical consistency among circulation, moisture and drought through time. In addition,

EULMDA provides observation-validated precipitation and PDSI fields back to 1000 CE. Among existing DA products, PHYDA does not provide precipitation fields and ModE-RA does not provide PDSI fields (ModE-RA also covers a shorter period). By contrast, EULMDA delivers a millennium-long reconstruction of summer precipitation and PDSI over Europe based on a particle filter, which does not rely on Gaussian assumptions and is therefore well suited to non-Gaussian variables such as precipitation. This dataset is therefore particularly suitable for studying millennial European hydroclimate variability and its mechanisms, which is the goal of our study.

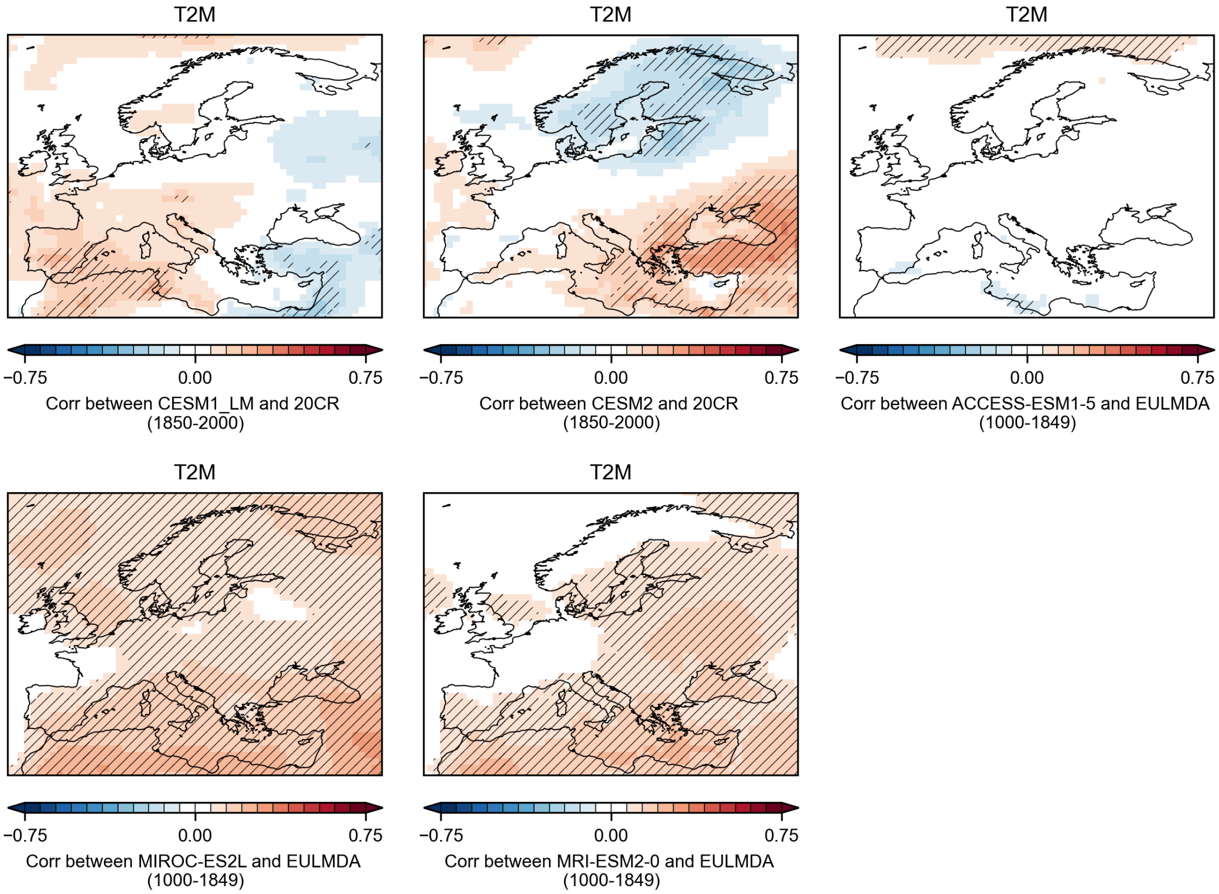

Figure R2. Temperature comparison between the prior model simulations and observation-based datasets. Maps show the correlation coefficient (r) of JJA near-surface temperature (T2M) over Europe between CESM1-LME and 20CR reanalysis (1850–2000 CE), CESM2-LE and 20CR reanalysis (1850–2000 CE), and between three additional prior simulations (ACCESS-ESM1-5,

MIROC-ES2L, MRI-ESM2-0) and EULMDA (1000–1849 CE). Shading denotes r; hatched areas indicate statistically significant correlations ($p < 0.05$).

2. The reconstruction exhibits a notable increase in climate variability (Supplementary Fig. 5). This raises a concern that the identified PDSI modes and their associated atmospheric circulation patterns may be dominated by the more recent centuries, particularly after 1600 CE or even 1850 CE. I suggest that the authors provide the principal component time series of the two leading modes (are Extended Data Figs. 6b and 6c the PC series of the NS mode?), which would help to assess whether these patterns are period dependent. Moreover, the role of these two modes in recent European hydroclimate variability could be more directly identified from the PC series.
We now provide the principal component (PC) time series of the two loading PDSI modes, with NS-mode PC shown in Supplementary Fig. 17a and PAN-mode PC shown in Supplementary Fig. 20a, which allow their temporal evolution and recent behaviour to be assessed directly.

To test whether the modes are dominated by the higher-variance recent period, we repeated the EOF analysis over two subperiods (1000–1500 and 1000–1850 CE). In both cases, NS- and PAN-like spatial patterns emerge, with spatial loadings very similar to those obtained for the full 1000–2000 CE period (Supplementary Fig. 11; Lines 163–166). This confirms that the NS and PAN modes are temporally robust features of European hydroclimate variability, rather than characteristics of some specific period only (in particular the last few centuries).

3. The reconstruction also exhibits a smaller overall amplitude compared to previous paleoclimate data assimilation products, particularly over the NE and WCE regions. This may suggest a systematic bias that should not be overlooked — especially if the variability during the instrumental period is also underestimated. I recommend that the authors further investigate this issue.
We thank the reviewer for this comment. We agree that variance damping is a limitation of paleoclimate data assimilation products and investigate it further here for EULMDA.

First, we quantify the amplitude of the EULMDA during the instrumental period by computing the ratio of the interannual standard deviation in EULMDA to that in instrument-

based products at each grid point, as well as for the NE, WCE and MED regional means (Supplementary Fig. 23). For JJA temperature, 81% of land grid cells show standard-deviation ratios between 0.5 and 1 relative to ERA5. For PDSI, 47% of land grid cells fall between 0.5 and 1 relative to CRU, and 27% relative to the Dai drought index, indicating moderate but not dramatic damping. In contrast, precipitation shows stronger variance reduction, with most land grid cells exhibiting ratios below 0.5. The regional mean series confirm that EULMDA reproduces year-to-year variations reasonably well (high correlations with instrumental products), but with somewhat reduced amplitude for temperature and PDSI, and more pronounced damping for precipitation (Supplementary Fig. 22). We now describe these results in the revised Supplementary Text (Lines S57–S67).

Second, we clarify why such damping can arise in our reconstruction. EULMDA is an ensemble-based data assimilation (DA) reconstruction that uses five climate models as priors, including some large ensembles, thereby providing a wide range of possible climate states. In an offline DA framework, the posterior at each time step is obtained by updating a static prior with the available observational (proxy) information. In this setting, **larger ensembles can reduce noise and improve skill, yet the posterior ensemble mean tends to damp variance** (e.g., Sjolte & Tao, 2025). In our case, the ensemble mean of the reconstruction does not reach the full variance of instrumental observations, but it represents the component of variability that is robustly constrained by the proxies, whereas the ensemble spread in EULMDA quantifies the range of plausible variability.

Moreover, the variance that can be expressed in the posterior mean strongly depends on the density and quality of proxy information. Where the proxy network is dense and reliable, the posterior distribution is relatively tight, and the temporal variability of the ensemble mean is better constrained; where observational constraints are sparse or noisy, the posterior distribution is broader but the ensemble mean can become relatively "flat" in time, even though individual ensemble members exhibit much larger variance (Emile-Geay et al., 2025). **The uneven temporal variance in EULMDA therefore mainly reflects the limited proxy constraints during earlier centuries**: when the network is sparse, the posterior reverts toward the prior and the variance of the ensemble mean of the reconstruction decreases due to noise cancellation across weakly constrained ensemble members. Similar behaviour is also seen in other DA reconstructions (e.g., Hakim et al., 2016).

Finally, although variance "inflation" approaches (e.g., rescaling to match an instrumental target) can visually increase amplitude, they do not add any new information and may simply introduce additional noise if not independently constrained (Sjolte & Tao, 2025). We therefore prefer not to impose an ad hoc variance correction. Instead, we discuss the variance damping in the main text (Lines 140–146) and in the Methods (Lines 414–419).

References

Emile-Geay, J., Hakim, G. J., Viens, F., Zhu, F., & Amrhein, D. E. (2025). Temporal comparisons involving paleoclimate data assimilation: challenges and remedies. *J. Clim.*, *38*(5), 1365–1385.

Hakim, G. J., Emile-Geay, J., Steig, E. J., Noone, D., Anderson, D. M., Tardif, R., et al. (2016). The last millennium climate reanalysis project: Framework and first results. *J. Geophys. Res. Atmos.*, *121*(12), 6745–6764.

Sjolte, J., & Tao, Q. (2025). Climate field reconstructions for the North Atlantic region of annual, seasonal and monthly resolution spanning CE 1241-1970. *EGUsphere*, *2025*, 1–23.

4. For the first mode (i.e., the NS mode), the authors attribute it primarily to the SCAND pattern. However, according to Fig. 3e and previous studies (e.g., Bladé et al., 2012), the summer NAO can also produce a north–south dipole–like effect on European precipitation. The manuscript only considers the influence of the NAO on the second, pan-European mode, but its potential contribution to the NS mode also deserves further consideration.

Reference: Bladé, I., Liebmann, B., Fortuny, D. et al. Observed and simulated impacts of the summer NAO in Europe: implications for projected drying in the Mediterranean region. Climate Dynamics, 39, 709–727 (2012).

We agree that the summer NAO can produce a northwest–southeast dipole-like pattern in European **precipitation**, consistent with Bladé et al. (2012) and our own results (Fig. R3a–b, f). Because our NS mode is defined from PDSI, we further assess the influence of the summer NAO on **PDSI** variability and its potential contribution to the NS mode.

First, summer NAO-related sea-level pressure (SLP) anomalies are largely confined to western–northern Europe, with weak expression over the Mediterranean (Fig. R3c–d). This spatial structure differs from the NS-mode pressure pattern shown in Fig. 2c, which features a clear dipole between northern and southern Europe.

Second, regressing PDSI onto the NAO index yields a monopole-like response over most of Europe (Fig. R3e). Consistently, the NAO index is nearly uncorrelated with the NS-mode principal component (PC; $r = –0.08$), whereas it correlates strongly with the PAN-mode PC ($r = –0.82$, $p < 0.05$).

Third, to isolate the NAO's dynamical contribution to the NS mode, we remove its co-variability with European mean surface temperature (EMST) by regressing the NAO index onto EMST and using the residual ("temperature-removed" NAO). Regressing PDSI onto this residual NAO yields a northwest–southeast dipole-like pattern (Fig. R3g), and the correlation with the NS-mode PC increases only modestly to $r = 0.22$ ($p < 0.05$). This suggests that the dynamical NAO contribution explains only a small fraction of NS-mode variability.

Taken together, these results suggest that although the summer NAO can induce a northwest–southeast contrast in precipitation, its contribution to NS-mode variability is weak. We have added a brief discussion in the revised manuscript (Lines 180–186).

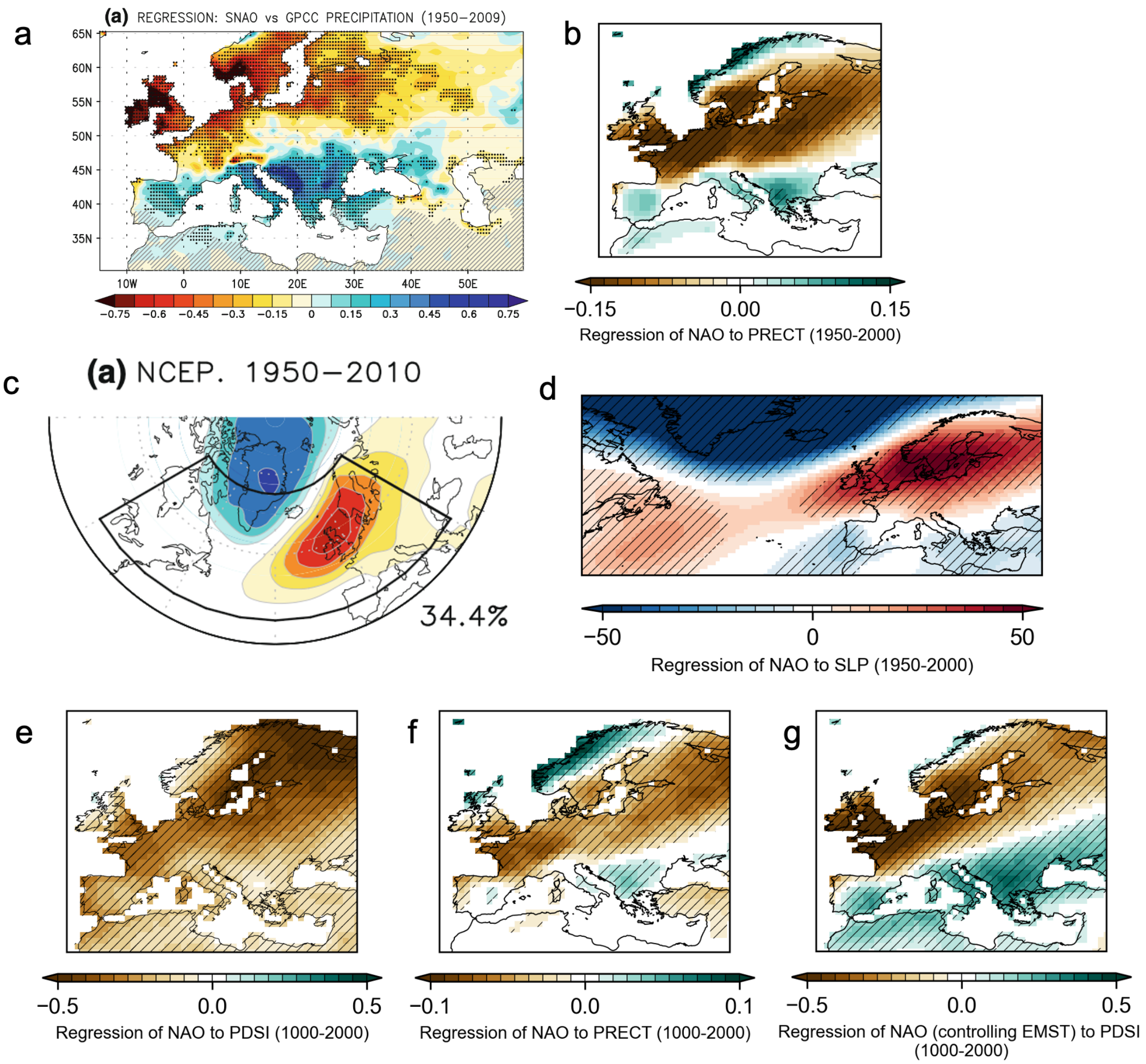

Figure R3. Summer NAO patterns and hydroclimate imprint. (a) Regression of GPCC precipitation onto the standardized summer NAO index defined for July–August (JA) for 1950–2009 (reproduced from Fig. 6a of Bladé et al. 2012). (b) Regression of JJA precipitation onto the JJA NAO index in EULMDA for 1950–2000 CE. (c) JA NAO sea-level pressure (SLP) pattern for 1950–2010 (reproduced from Fig. 1c of Bladé et al. 2012). (d) Regression of JJA SLP onto the JJA NAO index in EULMDA for 1950–2000. (e–f) Regression of JJA PDSI (e) and precipitation (f) onto the JJA NAO index in EULMDA over 1000–2000 CE. (g) Regression of JJA PDSI onto the residual NAO index after removing the covariance with EMST, over 1000–2000 CE.

5. For the second mode (i.e., the PAN mode), the authors attribute it mainly to the European Mean Surface Temperature (EMST), but the corresponding surface temperature field is not shown. Is the temperature anomaly also continent-wide, and how does the EMST drive or relate to the associated SLP pattern?

Yes, the temperature anomalies associated with both the PAN mode and EMST are also largely continent-wide. We now show the regression of JJA near-surface temperature onto the PAN-mode PC (revised Fig. 4b, Lines 288–290), which confirms widespread warming across Europe with the largest amplitudes toward northern Europe, supporting our interpretation that the PAN mode is primarily linked to temperature variability.

Regarding the associated SLP pattern, the PAN/EMST regressions show positive SLP anomalies over northern Europe that co-locate with the strongest warming (Fig. R4a–b), consistent with ridge-like conditions that co-vary with large-scale summer warming. The negative SLP anomalies over the Mediterranean are more complex and may involve additional low-level flow and associated advection.

To further clarify the linkage between the PAN mode and EMST and the large-scale circulation, we also examined regressions onto 800- and 500-hPa geopotential height (Z800 and Z500). Both EMST and the PAN mode show spatially coherent positive height anomalies over much of Europe at 800 and 500 hPa (Fig. R4c–f), consistent with a broad ridge associated with widespread summer warming. We therefore emphasize Z800/Z500 when discussing the PAN/EMST–circulation relationship (Lines 290–292; Supplementary Fig. 19).

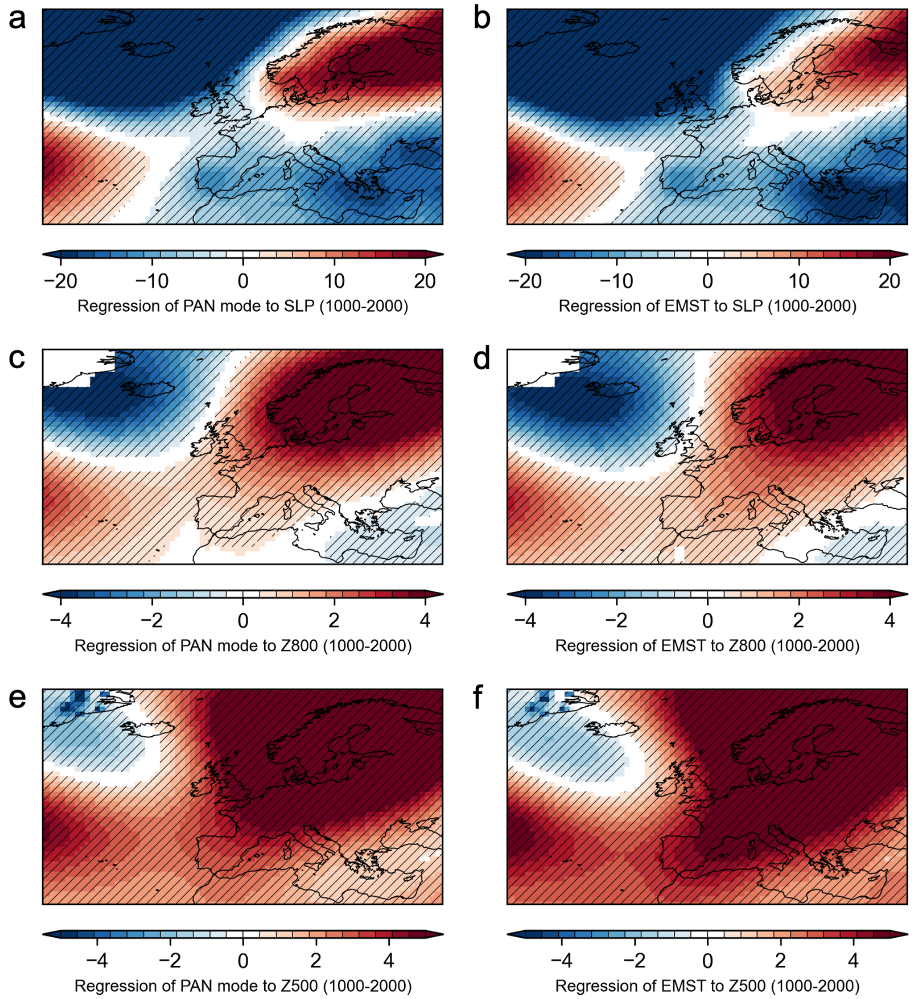

Figure R4. Large-scale circulation patterns associated with the PAN mode and EMST in EULMDA. (a–b) Regressions of JJA SLP onto the PAN-mode index (a) and EMST (b) over 1000–2000 CE. (c–d) As in (a–b) but for Z800. (e–f) As in (a–b) but for Z500. Hatched areas indicate statistical significance ($p < 0.05$).

6. Correlation is a key diagnostic metric in this study. However, when comparing Fig. 2d (regression of SCAND to SLP during 1000–2000) and Supplementary Fig. 8b (regression of PAN mode to SLP during 1000–2000), the two patterns appear quite similar. This raises the question of whether their correlation might even exceed that between Fig. 2d (regression of SCAND to SLP during 1000–2000) and Fig. 2c (regression of NS mode to SLP during 1000–2000). Could the authors quantify or comment on this potential similarity?

We thank the reviewer for raising this question. We now quantify these relationships and clarify the interpretation.

First, we computed both spatial and temporal correlations among the relevant modes. Over the Atlantic-European sector (40°W–40°E, 30–73°N), the spatial correlation between the SCAND–SLP and NS–SLP regression patterns is 0.84 ($p < 0.05$), whereas the correlation between SCAND–SLP and PAN–SLP is lower (0.64, $p < 0.05$). Consistently, the SCAND index correlates more strongly with the NS-mode PC ($r = 0.78$, $p < 0.05$) than with the PAN-mode PC ($r = 0.46$, $p < 0.05$). These results suggest that SCAND shares more variance with the NS mode than with the PAN mode.

Second, we confirm that the NS and PAN modes are strictly orthogonal by construction, with an inner product ≈ 0.

Third, because drought variability is influenced by moisture transport and near-surface flow captures only part of the column-integrated atmospheric transport, we also examine circulation patterns above the near-surface layer. At both 800 and 500 hPa, the SCAND-related (and NS-related) regressions retain the characteristic north–south dipole of the Scandinavian pattern (Supplementary Fig. 12a–d), whereas the PAN-related regressions form a spatially coherent pattern over most of Europe and closely resemble the EMST-related height anomalies (Supplementary Fig. 19). This supports that PAN/EMST and NS/SCAND correspond to different circulation regimes: the former is primarily associated with continent-wide warming and a broad ridge in the mid–upper troposphere, whereas the latter reflects a distinct dipole teleconnection pattern.

Therefore, while the SCAND–SLP and PAN–SLP regressions share some superficial similarities, the PAN mode is not another manifestation of SCAND; it is orthogonal to the NS mode and dynamically distinct from the SCAND pattern.

Minor comments:

1. On what timescale are the correlation coefficients and explained variances calculated? Specifically, were the values shown in the relevant figures and texts derived from filtered or unfiltered data? Please specify.

We have clarified in the relevant figure captions and texts that all correlation coefficients and explained-variance values are computed from annual anomalies, using unfiltered data.

2. For the related figures (i.e., Figs. 1–3, Extended Data Figs. 2-5, 9, Supplementary Figs. 1-2, 4, 7-9), please add more color bar values to improve clarity and facilitate interpretation.

We have revised the color bars in related figures to include more color bar labels.

3. Please specify the regional partitions shown in Extended Data Fig. 1.

We have now specified the regional partitions in revised caption of Supplementary Fig. 1a.

4. Lines 224: In Extended Data Fig. 7a, please indicate how many volcanic eruptions were included in the calculation and list which ones were used.

We now indicate the number of volcanic eruptions in Line 319, and list the eruptions used in the composite in Supplementary Tab. 2, and we cite this list in the revised Supplementary Fig. 20b.

5. Lines 227: In Extended Data Fig. 7b, the authors attribute the increase in EMST during 1960–2020 to anthropogenic forcing. However, its amplitude appears comparable to that of the 1910–1950 period.

We agree that Extended Data Fig. 7b (now revised as Supplementary Fig. 20c) shows two multi-decadal warming episodes in EMST during the twentieth century. Our intention was not to make an attribution of EMST based on this figure. To avoid any misleading impression, we have removed the original wording and now describe the co-variability between EMST and the PAN mode without assigning an attribution (Lines 320–325).

REVIEWER COMMENTS

Reviewer #1 (Remarks to the Author):

This is the revised version of a paper that I have reviewed previously. Although I had many comments, most of them were about clarifications of the methodology, which was poorly described and only few comments about the scientific findings and their interpretations. I am pleased by the authors' detailed responses to my comments, and I confirm that they are also reflected in changes in the manuscript. There are no new points I would like to raise, I am therefore happy with the manuscript. It is a relevant addition to the literature both with respect to climate reconstructions and with respect to atmospheric circulation variability and drought processes.

Thank you for your positive evaluation; we appreciate your feedback and are glad the revisions addressed your concerns.

Reviewer #2 (Remarks to the Author):

My comments have been addressed well. I recommend publication of the manuscript. I congratulate the authors on this high-quality study.

Thank you for your supportive comments and recommendation.

Reviewer #3 (Remarks to the Author):

I would like to commend the authors for their thorough and diligent revisions. The new analyses strengthen the overall results and provide valuable insights that will be of broad interest to the journal's readership. However, there are several comments that would benefit from further clarification, including concerns regarding the choice of model simulations and data processing details.

Thank you for your constructive comments. We provide point-by-point responses below.

Specific comments:

1. For the period 1850–2014, it is unclear why models that provide continuous coverage from 850 to 1850 simulations (e.g., ACCESS-ESM1, MIROC-ES2L, and MRI-ESM2-0) were not used, while CESM2—without 850–1850 simulations—was selected instead.

Our reconstruction window is 1000–2000 CE, and we use an offline DA framework in which the prior consists of a fixed pool of model states (i.e., simulated years are not aligned with reconstruction years). For each reconstruction based on a single model prior, we construct the prior pool using all available simulated states from that model (i.e., all simulated years × all members). The same prior pool is reused at each assimilation year; therefore, the prior does not provide temporal information on the reconstruction. The year-to-year variations in the posterior solely arise from the year-specific proxy constraints. CESM2-LE is included as one of the priors because it provides a large ensemble (100 members), which offers a broad sampling of climate variability. Our goal is to test whether the reconstruction is sensitive to the choice of the model ensemble as prior, and results using the five ensembles selected (see Supplementary Tab. 1) show that this is not the case.

2. For the first response, time coverage of CESM1-LME is given as 1850–2000, while it's described as 850–1850 in the Methods. Is this a typographical error?
We used, for this specific point in our response (first-round rebuttal), the simulated years 1850–2000 only, to compare CESM1-LME with 20CR and illustrate that free-running climate model simulations do not reproduce the observed internal variability with the correct phase. In the DA experiments, we use the simulated years 850–1850 from CESM1-LME to construct its prior.

3. For the second response (i.e., Supplementary Fig. 11), it may be helpful to use 1000–1500 and 1501–2000 as two subperiods for the EOF analysis. The results are unlikely to differ substantially, but this division may be more intuitive to interpret.
We agree that splitting the millennium into 1000–1500 and 1501–2000 is more intuitive. We repeated the EOF analysis using this division and found no substantive changes in the leading EOF patterns. We have updated Supplementary Fig. 11 accordingly.

4. For the large-ensemble simulations (i.e., CESM-LME and CESM2), were the ensemble members averaged before or after data assimilation? Please clarify. If they were averaged prior to assimilation, the resulting field would primarily represent externally forced variability, which may contribute to the variance damping issue noted in the third response.

Ensemble members are not averaged before assimilation. In our offline DA setup, each model year from each member is included in the prior as one particle and is weighted according to its likelihood knowing the observations. We further clarify this implementation in the Methods (Lines 441–443).

5. In the revised manuscript, the authors added geopotential height as a variable. I was wondering whether the 800 hPa level is obtained through interpolation, as model outputs are typically available at standard levels such as 850 or 700 hPa.

Yes, we interpolated geopotential height to 800 hPa using linear interpolation in pressure. We have clarified this at the first occurrence in the text (Line 96).

Review of

'Scandinavian pattern and temperature changes shape European summer droughts over the past millennium'

by Huihong Xue, Hugues Goosse, Quentin Dalaiden, Kristina Seftigen, Fabio Gennaretti, and Feng Shi

Recommendation: minor revisions

This manuscript presents the European Last Millennial Data Assimilation (EULMDA), a new reconstruction of European hydroclimate and its main drivers for the past millennium. It is based on off-line data assimilation with a particle filter that combines several hundred moisture and temperature-sensitive tree-ring records with simulations from 5 climate models. This data assimilation approach is well established, and a comprehensive evaluation of the specific new implementation against instrumental data, which is part of the manuscript, has demonstrated good performance.

After providing a review of the current understanding of thermodynamic and dynamic processes contributing to European droughts, the study uses EULMDA to address open questions related to the variability and causes of European summer droughts. The main finding is that natural variability of the Scandinavian pattern, which is one of the dominant large-scale atmospheric pressure variability modes in the European-Atlantic sector, is a key driver for soil moisture north-south gradients across Europe and for soil moisture variability in the Mediterranean region, which acts in addition to are anthropogenically forced, temperature-dependent thermodynamic processes.

The study provides substantial new findings that are of interest for a wide group of readers, and it is methodologically sound and well written. I therefore recommend publication after the specific points listed below have been addressed.

Specific comments

1)
It should be said in the introduction, in the evaluation section, and in the Fig.1 caption what the temporal resolution of EULMDA is, and whether the correlations in Fig. 1 are based on summer or annual means. These details become clear in the method section, but the information should also be given in the main text.

2)
Lines 121-122: The first part of this sentence is not correctly constructed.

3)
The regression maps in Figs. 2 and 3 are very informative. It should be noted that the regression of a time-dependent spatial field onto a time series, which is what these maps are, has a straightforward

interpretation when the time series is used as a predictor for the spatial field. However, for instance in the case of the SLP regression maps, the conceptual understanding is that the field anomalies are the predictors for the time series. It is not immediately obvious how the regression maps are linked to this estimation problem. It has been shown by Widmann (2005) that using the time expansion coefficient of the regression maps as the predictor for the time series (which is presumably what one has in mind when interpreting the regression maps) is equivalent to a 1-dimensional maximum covariance analysis (also known as Singular Value Analysis). It would be good to cite Widmann (2005) and clarify that using regression maps in the context of predicting the time series from the field has a sound theoretical foundation. Please also note that the regression of a time-dependent spatial field onto Principal Component Scores calculated from this field (as in Figs. 2a and 3a) is proportional to the Principal Components Loadings (aka EOF patterns) (this is a special case of 1-dimensional Maximum Covariance Analysis).

Widmann, M., 2005. One-dimensional CCA and SVD, and their relationship to regression maps. Journal of Climate, 18(14), pp.2785-2792.

4)
Lines 171-174: The contribution of the SCAND trend 1970 – 2000 (and potentially also for periods with more recent end dates) to regional PDSI trends, and to the trend in the north-south gradient of the PDSI should be quantified. For the regional trends this can be done by multiplying the SCAND trend during the reference period with the local PDSI on SCAND regression coefficients (i.e. with the regression maps) and dividing the result, which are the SCAND-driven local trends, by the actual local trends. For the north-south gradient this can be done by regressing the PDSI NS time series (i.e. the associated Principal Component Score) onto the SCAND time series, then multiplying with the SCAND trend, and dividing the result by the actual PDSI NS trend.

The contribution of the PAN trends could be quantified in the same way. The authors could also consider applying this approach to the regional trends in precipitation, and to the trends in north-south precipitation gradients. How to define the latter requires some thought. Options are Principal Component Score time series, or using the time expansion coefficient of the regression map of precipitation on the PDSI PAN and NS timeseries (which is getting a bit convoluted).

5)
Line 254: ‘where has undergone’ should be replaced by ‘which had undergone’.

6)
Line 256: Wrong citation format.

7)
Lines 271-282:
It is good that it is checked whether the relationships found in EULMDA are also present in instrumental data and reanalyses. However, it would be informative to do this not only for the Mediterranean region but also for the main relationships for the whole European region, most importantly for the link between the SCAN pressure and NS soil moisture patterns.
